# What factors influence the uptake of bowel, breast and cervical cancer screening? An overview of international research

**DOI:** 10.1093/eurpub/ckae073

**Published:** 2024-05-03

**Authors:** Sarah R Prowse, Miriam Brazzelli, Shaun Treweek

**Affiliations:** Health Services Research Unit, University of Aberdeen, Aberdeen, United Kingdom; Health Services Research Unit, University of Aberdeen, Aberdeen, United Kingdom; Health Services Research Unit, University of Aberdeen, Aberdeen, United Kingdom

## Abstract

**Background:**

For cancer screening programmes to be effective in early detection it is important that those invited can access screening services and understand the benefits of participation. A better understanding of the factors that matter to potential participants of cancer screening programmes can assist in developing strategies to increase current uptake.

**Methods:**

We conducted an overview of systematic reviews to answer the question: What factors influence the uptake of cancer screening services (breast, bowel and cervical) in high-income countries? A thematic approach supported by tabular summaries and qualitative heat maps was used to categorize factors, described as ‘barriers’ or ‘facilitators’.

**Results:**

A total of 41 systematic reviews met the criteria for inclusion. The barrier with the greatest number of ‘hot spots’ across all three screening programmes was a fear of the unknown regarding a possible diagnosis of cancer or abnormal screening results, followed closely by a general lack of knowledge surrounding cancer screening programmes. The greatest collective facilitator to uptake was recommendation by a healthcare provider to attend screening.

**Conclusion:**

Across all factors ‘trust’ and ‘building trusted relationships’ can be seen as integral to the success of cancer screening programmes and must be reflective of collaborative efforts to mitigate barriers and enhance facilitators to uptake. There is future scope to consider interventions that (i) increase demand for screening services, (ii) reduce barriers to uptake of services and/or (iii) are relevant to the healthcare system and those providing services.

## Introduction

The National Health Service (NHS) in the UK provides population-based screening programmes for bowel, breast and cervical cancer which are free to access and aim to detect cancer or precancerous activity in asymptomatic individuals. For screening programmes to be effective in early detection it is important that those invited can access screening services and understand the benefits of participation.

Historically, uptake of these offerings has varied over time within the NHS.^1–3^ Screening uptake has been shown to correlate with the number of cancers diagnosed at an earlier, and more treatable, stage.^4^^,^^5^ Invitations from the NHS are currently issued by age and at periodic intervals: ^6^

- Breast screening is offered to anyone registered with a general practitioner as female between the ages of 50 and 70, every 3 years.
- In England, everyone aged 60–74 is offered a bowel cancer screening home test kit every 2 years; in Scotland, the age range is 50–74 years and 55–74 years in Wales.
- Cervical screening is offered to all women and people with a cervix every 3 years for those aged 25–49, and every 5 years from the ages of 50–64.

Early diagnosis through screening improves health outcomes for patients and lessens the burden of required resources within a healthcare system.^7^ A better understanding of the factors that matter to potential participants of cancer screening programmes can assist in developing strategies to increase uptake.

This overview of systematic reviews aims to address the question: *What factors influence the uptake of cancer screening services (breast, bowel and cervical) in high-income countries?* Studying factors within all three cancer screening services offers the opportunity to reflect on programmes individually and identify commonalities.

## Methods

We conducted an overview of systematic reviews and registered the research protocol into PROSPERO, the National Institute for Health and Care Research International prospective register of systematic reviews (registration identification: CRD42023403479). The overview was guided by the recommendations of the Cochrane Handbook for Systematic Reviews of Interventions and the Joanna Briggs Institute (JBI) Manual for Systematic Reviews. We used the JBI Checklist for Systematic Reviews and Research Syntheses to account for the inclusion of qualitative and mixed-methods systematic reviews.^8^

### Search strategy

A comprehensive search strategy was developed with the assistance of an information specialist and is presented in Supplementary material 1. MEDLINE (Ovid), the Cochrane Database of Systematic Reviews, and the Database of Abstracts of Reviews of Effects were searched for systematic reviews and systematic scoping reviews published from 01 January 2012 to 31 January 2023. A 10-year period was selected with our NHS partners to best capture the most relevant factors related to uptake in the context of modern screening programmes. No language restrictions were initially applied, with English language used as the final limiting filter. Searches were not rerun prior to the final analysis.

### Eligibility criteria

This overview is international in scope and focussed on high-income countries with healthcare systems similar to the UK. The World Economic Situation and Prospects 2023 report from the United Nations was used to identify countries classified as high-income.^9^ Reviews for inclusion focussed on factors that matter to potential participants of bowel, breast and/or cervical cancer screening programmes. It was agreed that screening must be the primary focus, not a wider component, of included reviews.

Reviews were excluded if studies were not exclusively conducted in high-income countries, incorporated indications other than bowel, breast and/or cervical cancer, focussed on genetic cancer screening (e.g. BRCA1/BRCA2, Lynch syndrome), or highlighted screening procedures beyond routine uptake. Guidelines, economic analyses, and reviews of solely interventional research were not eligible for inclusion.

### Data extraction

All citations retrieved through the search process were initially assessed by title and abstract based on the inclusion and exclusion criteria. One reviewer assessed all citations with each second reviewer blind assessing a differing 10% of citations, giving a total of 20%, to ensure consistency in the selection process.

All citations judged to be potentially relevant were retrieved in full and reviewed by one reviewer, with each second reviewer blind assessing a differing 10%, for a total of 20%, to ensure uniformity in the interpretation of eligibility criteria. Any disagreements or uncertainty during the selection process was resolved by consensus between reviewers. Citations and inclusion/exclusion rationale were recorded using Microsoft Excel.

A data extraction form was designed to capture review specifics such as search strategies and methods, details of included studies, and key findings including implications for future research. The full data extraction forms are available in Supplementary material 2.

### Critical appraisal

The JBI Checklist for Systematic Reviews and Research Syntheses was used to critically appraise the methodological quality of included systematic reviews.^10^ It was agreed that Question 8 of the JBI checklist be amended to reflect the range of methods more broadly within the reviews and was modified to read: ‘Were the methods used to combine *and/or summarise* studies appropriate?’.

Reviews were not excluded based on their methodological quality, which was assessed to inform the robustness of findings as well as identify common areas for methodological improvement across reviews. The results of the critical appraisal are included in Supplementary material 3.

### Synthesis and analysis

We decided *a priori* to acknowledge the heterogeneous nature of the existing reviews, and to be guided by the evidence presented by the authors of the identified reviews when considering the diverse factors influencing the uptake of bowel, breast and/or cervical cancer screening.

Factors, commonly described in the literature as either ‘barriers’ or ‘facilitators’, were first identified and highlighted by one reviewer using NVivo 12.0 and then discussed collaboratively. As the presentation of results varied widely across reviews, only those factors that were described by the authors as integral or key to understanding were captured for further discussion. Factors were discussed in depth by the review group (SP, MB, ST) and organized in common categories as supported by previous similar research for vaccine uptake.^11^

Recoding of items that covered comparable content was performed by consensus. For example, ‘physician recommendation as a cue to action’,^12^ ‘having a clinician recommendation for the test’^13^ and ‘healthcare provider recommendation’^14^ were recoded to the wider line item of ‘*healthcare provider recommendation to attend screening*’. It was agreed that monetary factors such as insurance or privatized healthcare, as noted in studies from the USA, would not be included in the analysis as these factors are not relevant within the context of NHS population-based screening programmes in the UK and other similar international counterparts.

All data were synthesized using a thematic approach as described above. A thematic approach was used to support an inductive process of coding by identifying, describing and interpreting themes within a multifaceted research question. Codes and descriptive themes were developed from the data to provide insights into factors influencing screening uptake. The thematic analysis is supported by tabular summaries and visual heat maps with factors frequently mentioned coloured red, and those rarely mentioned coloured green. Factors that were not mentioned are coloured grey. A red square is, therefore, a ‘hot spot’ for action (see Supplementary material 6).

Analysis of the implications and recommendations for future research as described by the authors of the reviews was also undertaken using the same methodology, with key ideas highlighted in NVivo 12.0 by one reviewer and further synthesized using a thematic approach with tabular summaries and supporting quotes. As this is an overview of systematic reviews in varying contexts, no meta-analytical synthesis of findings was undertaken.

## Results

**Figure 1:**
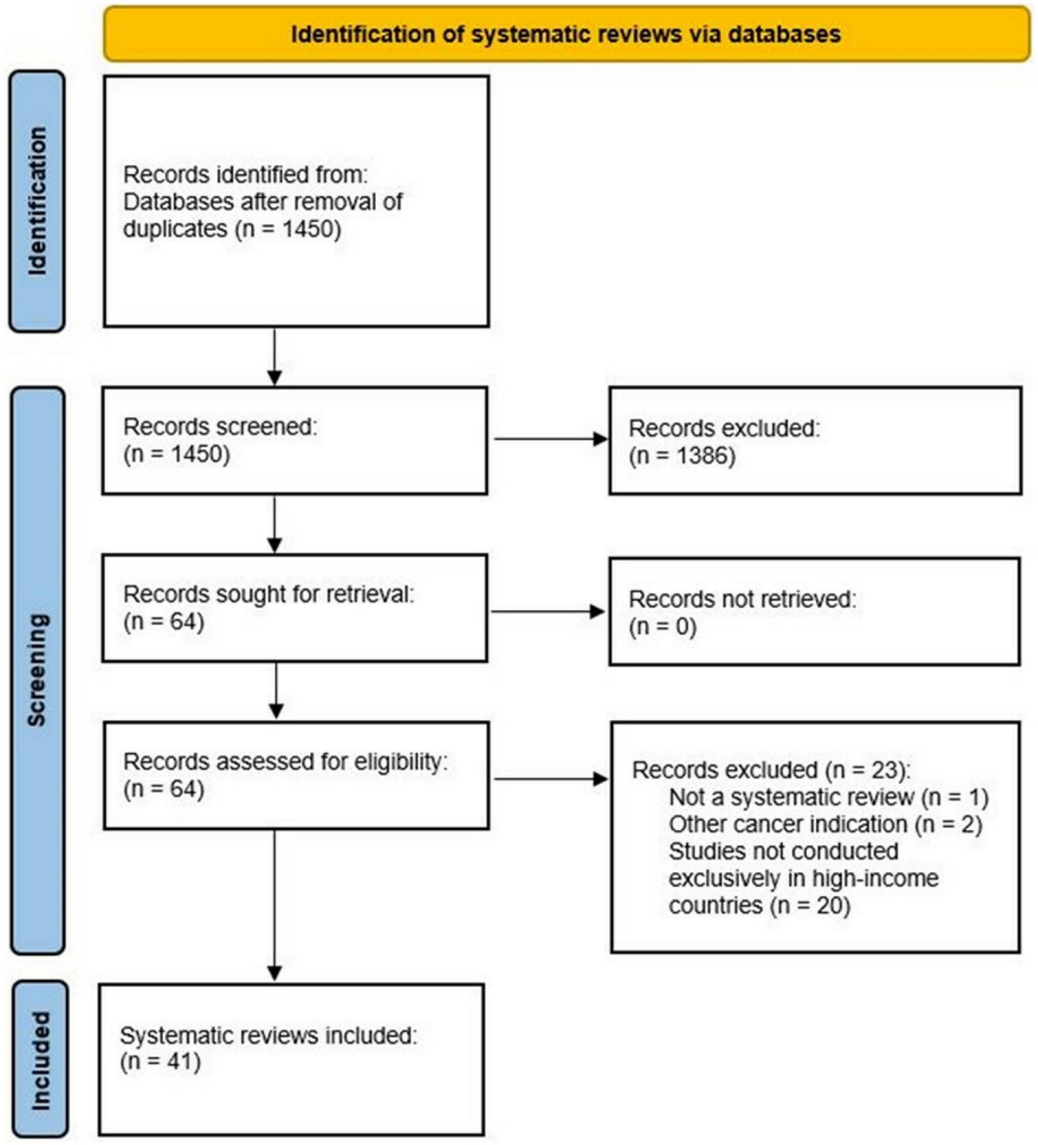
Results of the search process.

Figure 1 summarizes the results of the searches and selection process in a PRISMA-style diagram. We identified 1450 citations after the removal of duplicates. After title and abstract screening, 64 records were reviewed in full text with 41 systematic reviews included in the final overview. Of these, 16 reviews (39%) focussed solely on factors related to bowel screening; 15 (37%) on cervical cancer screening and 6 (15%) on breast cancer screening. The remaining reviews provided a combined synthesis of factors relevant to screening uptake in breast and cervical programmes (2/41, 5%) and bowel, breast and cervical programmes (2/41, 5%).

### Description of the included reviews

The main characteristics of the included systematic reviews, which varied widely given the heterogeneous nature of the overview, are presented in Supplementary material 4. Sixteen reviews (39%) focussed on the uptake of screening as directly relevant to ethnicity, while an additional 10 reviews (24%) addressed under-served groups more widely. Geographically, 21 reviews (51%) incorporated studies from multiple high-income countries. Studies from the United States were included in most reviews (32/41, 78%) followed by European countries broadly (21/41, 51%).

### Overview of factors influencing the uptake of cancer screening services in high-income countries

A total of 12 categories were used to delineate the factors influencing the uptake of cancer screening services. Within this categorization a total of 45 barriers were identified across all three screening programmes (see table 1) with 15 barriers (33%) common to all screening programmes. The barrier with the greatest number of ‘hot spots’ for low uptake across all three screening programmes was a fear of the unknown regarding a possible diagnosis of cancer or abnormal screening results.

**Table 1:** Barriers to the uptake of cancer screening services

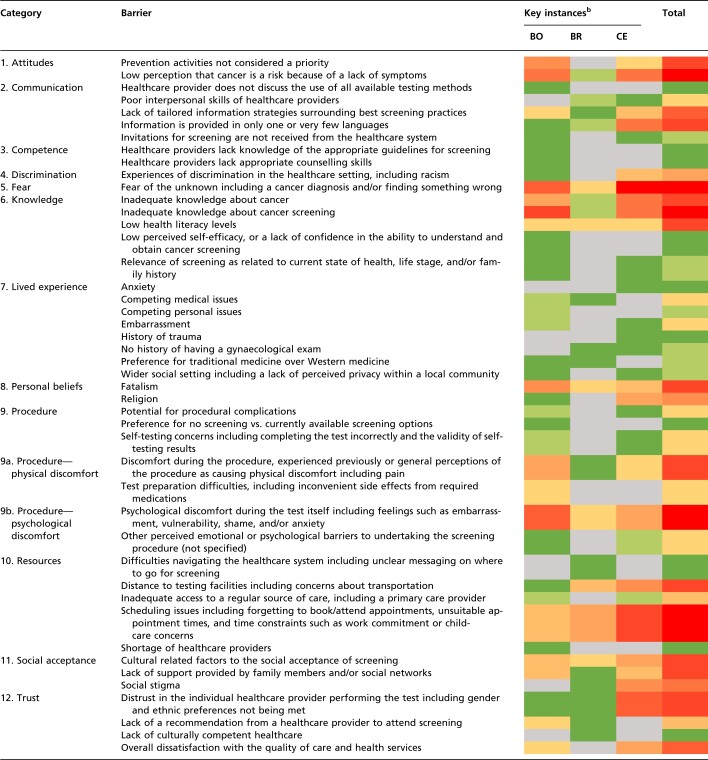

Gradient colour key^a^

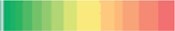

Least → Most observed

a Barriers that were frequently mentioned are coloured red, while those rarely mentioned are coloured green. Barriers that were not mentioned at all are coloured grey. A red square is, therefore, a hot spot for action.

b BO, Bowel; BR, Breast; CE, Cervical.

The most reported barrier to the uptake of bowel cancer screening was an inadequate knowledge of the screening programme itself. Scheduling issues were the most common barriers for breast cancer screening programmes including forgetting to book or attend appointments, unsuitable appointment times, and time constraints such as work commitments or childcare concerns. Fear of the unknown was the most observed barrier for uptake of cervical cancer screening programmes.

Fewer facilitators to the uptake of cancer screening were found within the reviews as shown in table 2. Twenty-nine facilitators were identified across 9 categories. Of these, 15 were common across all three programmes (52%) with the greatest collective facilitator to uptake being the recommendation by a healthcare provider to attend screening.

**Table 2:** Facilitators to the uptake of cancer screening services

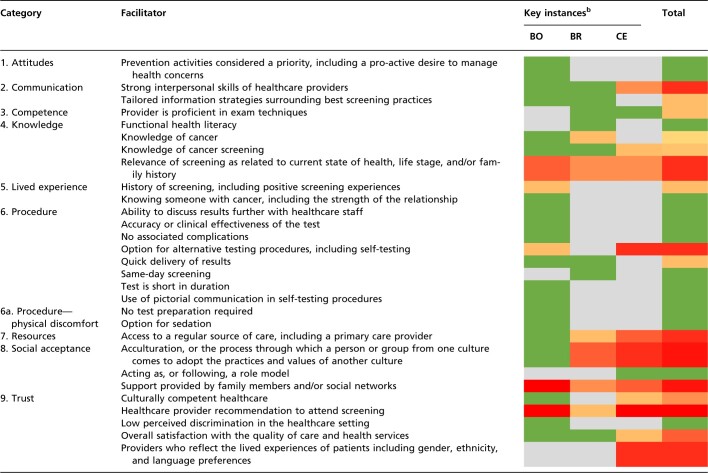

Gradient colour key^a^

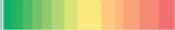

Least → Most observed

a Facilitators that were frequently mentioned are coloured red, while those rarely mentioned are coloured green. Facilitators that were not mentioned at all are coloured grey. A red square is, therefore, a hot spot for action.

b BO, Bowel; BR, Breast; CE, Cervical.

Support provided by family members and/or social networks along with a healthcare provider recommendation to attend screening were the most cited facilitators within bowel screening programmes. Acculturation, described by the reviews as the process through which a person or group from one culture comes to adopt the practices and values of another culture, was found to be the main facilitator of breast cancer screening uptake.^15^ This is reflective of the included qualitative data, as most of the reviews incorporating breast cancer screening (8/10, 80%) focussed specifically on the perspectives of one or more ethnic groups. The recommendation of a healthcare provider to attend screening was the greatest individual facilitator of cervical cancer screening uptake, with other key facilitators being practitioners who reflect the lived experiences of participants and options for procedural self-testing.

### Key recommendations from the systematic reviews

Recommendations for future research and best practice were also collated and synthesized from the included systematic reviews. Five priority areas were identified as outlined in table 3. This includes maximizing the trusted role of healthcare providers, prioritizing patient-centred care and patient autonomy, culturally tailored health promotion, systematically reducing health inequalities through policy, and informed research design. Further illustrative summaries and quotes are available in Supplementary material 5.

**Table 3:** Priority areas for future research and best practices in cancer screening programmes as identified by the reviews

i. Maximizing the trusted role of healthcare providers	
*Recommendation*	*As illustrated in the reviews*
a. Health providers may require additional support and training to enhance their knowledge of cancer and screening programmes. Effective training includes awareness of barriers to screening within under-served groups, consistently identifying those at higher risk of cancer such as first-degree relatives, and up-to-date knowledge on relevant screening guidelines and policies.	*‘…there is an urgent need for basic education surrounding the healthcare needs of gender minority patients [assigned female at birth], so that clinicians are responsive to each individual’s needs and skilled in a range of approaches to cervical cancer screening.’* ^42^
b. Communication skills have been identified by the reviews as integral to improving overall willingness to attend screening and satisfaction with screening services. Healthcare providers should be made better aware of the impacts both at the personal and public health levels of their endorsement to attend screening.	‘*The patients' decision to attend screening is directly influenced by their encounters with healthcare staff. There is, therefore, a real need to better educate healthcare staff on the public health implications following their patient interaction*’.^16^
c. Internal reminder systems for a primary care team may also be useful in ensuring recommendation for screening is delivered at appropriate intervals.	*‘The presence of an effective reminder system for the primary care team and for patients can also be helpful’.* ^43^
**ii. Prioritizing patient-centred care and patient autonomy**	
*Recommendation*	*As illustrated in the reviews*
a. The preferences of potential participants should be considered when undertaking cancer screening, including timely response to individual needs and values. Overarching patient-centred care strategies to increase uptake include considering the role of provider gender, language barriers, and differing lived experiences that may impact willingness to undertake cancer screening.	*‘Failing to account for the specific preferences of medically underserved individuals will allow the disparities in cervical cancer incidence and mortality to continue widening’.* ^17^
b. Individualized options for procedural processes may alleviate both physical and psychological distress associated with cancer screening.	*‘…increased emphasis needs to be placed on efforts to improve the bowel preparation process, enhancing comfort and modesty during the examination and identifying patients with significant anxiety beforehand’.* ^13^
c. Further educational materials, including complex terminology and/or visual aids, are needed to better inform the process of consent in cancer screening.	*‘…it is important for genuinely informed consent that people are not overwhelmed with information. Some terminology, for example, “high-risk HPV” and “wart virus”, was confusing or alarming and not clearly understood. Explanations about causation, risk of cervical abnormality or cancer, persistence/clearance of monogenic HPV and difference from genital wart viruses are needed’.* ^18^
**iii. Culturally tailored health promotion**	
*Recommendation*	*As illustrated in the reviews*
a. There is a lack of culturally tailored health promotion as a ‘one-size-fits-all’ approach fails to consider the heterogeneity within differing ethnic communities. Engagement strategies should be designed by and for specific communities, focussed on building trust and clear communication with available health services.	*‘Relevant programs should be aware of the importance of alternative sources in reaching the population as well as the limited quality of such sources. The dissemination of accurate cancer screening and prevention information, customized to the population’s needs through their preferred information sources should be prioritized, as well as offering more opportunities to increase health/cancer literacy skills’.* ^19^
b. Culturally sensitive educational interventions are needed at both the participant and provider level. Potential participants will benefit from interventions that are communicated in a culturally sensitive and linguistically appropriate manner. Providers require further education and training on best practices for delivering care and screening recommendations to minority populations.	*‘When designing interventions to increase screening uptake among immigrants, gaps in physician and screening education, access to care, and trust need to be addressed through culturally sensitive supports. These interventions should be tailored to the specific immigrant group, since a one-size-fits approach fails to consider the heterogeneity within this population’.* ^20^
**iv. Systematically reducing health inequalities through policy**	
*Recommendation*	*As illustrated in the reviews*
a. Strong public policies are needed that both promote and create awareness of different cancer screening procedures. This includes policies for underserved groups which can benefit from a process of decision making tailored to circumstances that may otherwise prevent the uptake of cancer screening.	‘*Government and policy makers might revise their strategies to promote screening uptake in other ways […] interventions directed at these levels may help to improve the screening uptake by increasing the facilities available and accessible, supporting childcare, having female physicians speaking the minority’s language, and overcoming culture-related influences that deter screening’.*^21^
b. Policy makers should also consider the role of trust in achieving appropriate uptake of cancer screening. Healthcare providers can capitalize on their trusted influence and join policy makers in designing and delivering appropriate strategies for cancer screening.	*‘Specifically supported in our findings is the suggestion medical providers capitalize on their influence and join policy makers in efforts to eliminate colorectal cancer screening disparities…’* ^14^
**v. Informed research design**	
*Recommendation*	*As illustrated in the reviews*
a. Future research should consider multimodal or mixed methods approaches that go beyond the clinical setting to reach potential participants who do not currently undergo regular cancer screening.	*‘Future studies would benefit from a mixed methods approach, including both high quality intervention studies alongside qualitative studies to explore barriers and enablers to screening participation.’* ^22^
b. Further appraisal is needed of current interventions to increase screening uptake (e.g. bowel, breast, and cervical programmes) to assess how the intervention agenda can be revised to better align with inclusive best practices in preventative health.	*‘Appraisal of existing UK-wide NHS interventions to increase [flexible sigmoidoscopy screening] uptake, which are largely paper based, require further validation regarding their effectiveness…’* ^23^

## Discussion

This overview of existing reviews aimed to capture the factors, defined as barriers and facilitators, to uptake within bowel, breast and/or cervical cancer screening programmes in high-income countries. However, there are clear practical differences in how the programmes are delivered that influence these factors. Bowel screening programmes reflected the most variability in process, offering screening options within a healthcare setting and self-testing options performed at home. Both physical and psychological discomfort were expressed as key barriers to uptake due to the invasive nature of some bowel procedures such as a colonoscopy or sigmoidoscopy. These options also require an element of preprocedure bowel preparation via laxative treatment and/or dietary changes. Test preparation difficulties were a noted barrier to uptake with one review suggesting the preparation element potentially impedes individuals from repeat screening.^23^

While there was evidence for self-testing options as facilitators to bowel cancer screening, concerns raised by potential participants as barriers included completing the test incorrectly and the validity of self-test results.^24^^,^^25^ Some participants were found to prefer a colonoscopy as ‘they believed it to be a more thorough examination’.^25^ These factors offer some insight when reflecting on the general lack of knowledge surrounding bowel cancer screening and subsequent facilitators to alleviate misunderstandings. For example, the use of pictorial communication in self-testing procedures was noted as a facilitator to address concerns when completing an immunochemical faecal occult blood test or faecal occult blood test.^24^ Both tests, which are widely integrated to screening programmes across high-income countries, involve a self-sampling of stool which is then typically returned to a lab for further diagnostic review.^24^^,^^25^ Clear communication for self-testing procedures may also contribute to enhanced trust within the healthcare system, including alleviating fears of screening procedures and potential health outcomes.

There are no readily available global self-testing options for breast cancer screening. While self-administered breast exams are recommended by the NHS and other similar healthcare systems, ultimately a diagnosis cannot be obtained without further clinical investigations.^26^ All six reviews that focussed solely on breast cancer screening referred to the requirement of participants to attend screening in-person due to the use of x-ray mammography.^12^^,^^16^^,^^27–30^ Scheduling issues were the most cited barrier to uptake, including perceived time constraints to attending a screening appointment and childcare concerns. These were closely followed by the barrier of distance to testing facilities. Two reviews that focussed on rural geographies identified lack of transportation as a key aspect when considering the distance to testing facilities.^28^^,^^31^ While interventions were not within the scope of the current overview, only one review discussed the use of mobile mammography to assist with resource concerns in relation to breast cancer screening.^28^

The barrier of fear was most clearly seen as related to cervical cancer screening. This referred to not only the potential of a cancer diagnosis but general fear surrounding the results of human papillomavirus (HPV) testing. Confusion also persists surrounding HPV screening vs. a Papanicolaou (Pap) test.^18^ HPV screening aims to identify the virus which may cause cell changes to the cervix and eventually lead to cancerous outcomes. In contrast, a Pap test looks for precancerous cell changes on the cervix itself that may become cancerous if not treated promptly. A positive outcome in both testing scenarios is not necessarily indicative of a cancer diagnosis, with differing treatment options available. Reviews were explicit in the need for better informed choice in cervical cancer screening to alleviate fears surrounding testing procedures and potential outcomes.^17^^,^^18^^,^^32^

The option for HPV self-sampling was also a potential facilitator of cervical cancer screening uptake. While self-testing options exist in some high-income countries, these are not yet readily available worldwide. Findings from a 2022 article focussed on the global use of HPV self-sampling for cervical cancer screening noted six high-income countries with self-testing approaches in national programmes and/or guidelines (Australia, Denmark, Finland, France, the Netherlands and Sweden).^33^ Multiple other countries, including those grouped as low- and lower middle- income, had also implemented self-testing approaches or were piloting new programmes.^33^ Scheduling issues were a barrier for those attending cervical cancer screening which could be circumvented with the introduction of self-sampling options in the first instance. This approach has also been shown to better reach those with irregular access to health care or in areas where health facilities may be less accessible.^17^^,^^18^

Distrust in the individual healthcare provider performing the test was observed in a broad capacity across all three programmes, most notably as a ‘hot spot’ barrier for cervical cancer screening. Potential participants preferred providers who reflected their own lived experiences including aspects such as gender, ethnicity and language.^21^^,^^34^^––^^36^ The element of *trust* was consistent throughout the reviews, including discussions of satisfaction with the quality of care and health services and the overarching facilitator of a healthcare provider recommendation to attend cancer screening. Healthcare providers were typically identified as nurses, general practitioners or primary health care providers, and specialists such as oncologists. The role of a trusted healthcare provider was also reflected in the implications for future research and best practice as recommended by the authors of the included reviews. Recommendations included additional support and training for providers to enhance already existing knowledge of cancer and screening programmes as well as counselling practices for both the wider public and under-served communities.

The need for culturally tailored health promotion was reflected in both barriers and facilitators throughout the reviews. Experiences of discrimination within the healthcare system were noted by five reviews describing differing aspects such as racism, legacies of colonialism and challenges faced by rural communities.^22^^,^^32^^,^^35^^,^^37^^,^^38^ Wider cultural phenomena were also highlighted such as the heteronormative role of masculinity in the acceptability of procedures involving the rectum.^39^ Culturally tailored health promotion may also be reflected in barriers such as language preferences and/or low levels of health literacy, both of which were noted as contributing to an overall lack of knowledge regarding cancer and cancer screening programmes. Recommendations for future research in this area often overlapped with concepts of prioritizing patient-centred care and patient autonomy. A ‘one-size-fits-all’ approach to cancer screening fails to consider the heterogeneity within differing cultural traditions.^19^^,^^20^ It was recommended that engagement strategies be designed by and for the individuals within a specific community, focussed on building trust and clear communication with available health services.^19^^,^^20^ Established examples of such strategies include community-based participatory research and integrated knowledge translation.^40^^,41^

While capturing information about interventions was not part of this overview, the barriers and facilitators presented can further guide discussions on best practices for future research. As evidenced by the reviews there is scope to consider interventions that (i) increase demand for screening services, (ii) reduce barriers to uptake of services and/or (iii) are relevant to the healthcare system and those providing services. The latter also reflects the need to address systemic inequalities through additional measures at the policy level that aim to promote and create awareness of different cancer screening opportunities and procedures. Across reviews, tailored information strategies were described as both barriers and facilitators to screening uptake, with some discussion around the role of media and educational materials that are fit-for-purpose.^18^^,^^19^^,^^24^

This overview was not without limitations. By addressing three cancer screening programmes in tandem, a granular assessment of barriers and facilitators was not feasible. We also did not seek an equitable distribution between reviews focussed on barriers vs. facilitators (or a combination thereof). The key factors presented, and the synthesis of future practice recommendations, are intended to provide an overarching reflection on issues that are both unique and common across bowel, breast and/or cervical cancer screening efforts. The depth of barriers and facilitators presented by the included reviews also varied widely in their presentation, with some offering contextual details and others lacking this additional description. Facilitators were comparatively under-researched (or under-reported) and may offer a potential avenue for future research and development.

We sought to identify and discuss the factors influencing uptake of cancer screening programmes and not which groups of people are most or least likely to participate. As with any overview of existing reviews, the search process itself was also limited in its capacity, including the decision to restrict inclusion to reviews published in three key databases, and to only incorporate studies from high-income countries published in English. We sought to balance the thoroughness of the search with the needs of those commissioning the research with regard to the use of time and money. We used the JBI Checklist for Systematic Reviews and Research Syntheses to assess the methodological quality of the included reviews, but we did not attempt to reassess the risk of bias of the individual studies included in each review. Furthermore, we did not quantify the degree of overlap between reviews in terms of included studies. Pre-established frameworks or guiding theories were also not incorporated into the analysis. This overview sought to offer overarching insights for multiple cancer screening programmes, and choosing one framework or theory as a lens for analysis could potentially limit the applicability of the insights. Additional research that is fit-for-purpose within cancer screening programmes is needed to further contextualize the identified factors as they relate to uptake.

In conclusion, this overview acknowledges a need for further informed research design when considering the barriers and facilitators that may influence the uptake of cancer screening programmes. A key recommendation identified within the reviews is to consider multimodal or mixed methods approaches to research that go beyond the clinical setting to reach potential participants who do not currently undergo regular cancer screening.^22^ There is also a need to further appraise current interventions across programmes to discuss how the intervention agenda can be revised at both local and national levels to better align with inclusive practices in preventative health.^23^ As shown by the findings of this overview, across all factors ‘trust’ and ‘building trusted relationships’ with diverse communities are integral to the success of cancer screening programmes and must be reflective of collaborative efforts to mitigate barriers and enhance facilitators to uptake.

## Supplementary Material

ckae073_Supplementary_Data

## Data Availability

The data underlying this article are available in the article and in its online supplementary material.

## Supplementary data

Key points

- A better understanding of the factors that matter to potential participants of cancer screening programmes can assist in developing strategies to increase current uptake.
- The barrier with the greatest number of ‘hot spots’ across bowel, breast, and cervical screening programmes was a fear of the unknown regarding a possible diagnosis of cancer or abnormal screening results, followed closely by a general lack of knowledge surrounding cancer screening programmes.
- The greatest collective facilitator to uptake across bowel, breast, and cervical screening programmes was the recommendation of a healthcare provider to attend screening.
- Across all factors the element of ‘trust’ and ‘building trusted relationships’ can be seen as integral to the success of cancer screening programmes and must be reflective of collaborative efforts to mitigate barriers and enhance facilitators to uptake.
- There is future scope to consider interventions that (i) increase demand for screening services, (ii) reduce barriers to uptake of services and/or (iii) are relevant to the healthcare system and those providing services.

## References

[1] NHS Digital. NHS Breast Screening Programme, England 2020-21. Available at: https://digital.nhs.uk/data-and-information/publications/statistical/breast-screening-programme/england—2020-21 (11 July 2023, date last accessed).

[2] NHS Digital. National Bowel Cancer Audit 2023. Available at: https://digital.nhs.uk/data-and-information/clinical-audits-and-registries/national-bowel-cancer-audit (11 July 2023, date last accessed).

[3] NHS Digital. NHS Cervical Screening Programme, England 2020-21 2022. Available at: https://digital.nhs.uk/data-and-information/publications/statistical/cervical-screening-annual/england—2020-2021 (11 July 2023, date last accessed).

[4] NHS Digital. NHS CCG Outcomes Indicator Set—October 2022: 1.18 Percentage of cancers detected at stage 1 and 2 2022. Available at: https://digital.nhs.uk/data-and-information/publications/statistical/ccg-outcomes-indicator-set/october-2022/domain-1-preventing-people-from-dying-prematurely-ccg/1-18-percentage-of-cancers-detected-at-stage-1-and-2 (11 July 2023, date last accessed).

[5] National Cancer Registration and Analysis Service (NCRAS). COVID-19 rapid cancer registration and treatment data 2023. Available at: https://www.cancerdata.nhs.uk/covid-19/rcrd (11 July 2023, date last accessed).

[6] National Health Service (NHS). NHS screening 2018. Available at: https://www.nhs.uk/conditions/nhs-screening/ (11 July 2023, date last accessed).

[7] World Health Organization. National Cancer Control Programmes: Policies and Managerial Guidelines, 2nd edn. World Health Organization, 2002. Available at: https://apps.who.int/iris/handle/10665/42494

[8] Pollock M , Fernandes RM, Becker LA, et al Chapter V: Overviews of Reviews. In: Higgins JPT, Thomas J, Chandler J, Cumpston M, Li T, Page MJ, Welch VA (editors). *Cochrane Handbook for Systematic Reviews of Interventions version 6.4 (updated August 2023).* Cochrane, 2023. Available from www.training.cochrane.org/handbook.

[9] United Nations. World Economic Situation Prospects 2023. Available at: https://desapublications.un.org/publications/world-economic-situation-and-prospects-2023 (10 July 2023, date last accessed).

[10] Aromataris E , Fernandez R, Godfrey CM, et al Summarizing systematic reviews: methodological development, conduct and reporting of an umbrella review approach. Int J Evid Based Healthc 2015;13:132–40. 26360830 10.1097/XEB.0000000000000055

[11] Collaboration for Change. Collaboration for Change: Promoting Vaccine Uptake 2021. Available at: https://collaborationforchange.co.uk/factors-strategies/ (13 November 2023, date last accessed).

[12] Jerome-D’Emilia B. A systematic review of barriers and facilitators to mammography in hispanic women. J Transcult Nurs 2015;26:73–82. 24797255 10.1177/1043659614530761

[13] McLachlan S-A , Clements A, Austoker J. Patients’ experiences and reported barriers to colonoscopy in the screening context—A systematic review of the literature. Patient Educ Couns 2012;86:137–46. 21640543 10.1016/j.pec.2011.04.010

[14] Rogers CR , Goodson P, Foster MJ. Factors associated with colorectal cancer screening among younger African American men: a systematic review. J Health Dispar Res Pract 2015;8:133–56. 26435888 PMC4590998

[15] National Library of Medicine. Acculturation: MeSH Descriptor Data 2023. Available at: https://meshb.nlm.nih.gov/record/ui?ui=D000069 (13 November 2023, date last accessed).

[16] Baird J , Yogeswaran G, Oni G, Wilson EE. What can be done to encourage women from Black, Asian and minority ethnic backgrounds to attend breast screening? A qualitative synthesis of barriers and facilitators. Public Health 2021;190:152–9. 33419526 10.1016/j.puhe.2020.10.013

[17] Biddell CB , O'Leary MC, Wheeler SB, Spees LP. Variation in cervical cancer screening preferences among medically underserved individuals in the United States: a systematic review. Cancer Epidemiol Biomarkers Prev 2020;29:1535–48. 32457182 10.1158/1055-9965.EPI-20-0306 PMC7415615

[18] Hendry M , Pasterfield D, Lewis R, et al Are women ready for the new cervical screening protocol in England? A systematic review and qualitative synthesis of views about human papillomavirus testing. Br J Cancer 2012;107:243–54. 22699825 10.1038/bjc.2012.256 PMC3394982

[19] Jun J , Nan X. Determinants of cancer screening disparities among Asian Americans: a systematic review of public health surveys. J Cancer Educ 2018;33:757–68. 28378200 10.1007/s13187-017-1211-x

[20] Puli AV , Lussiez A, MacEachern M, et al Barriers to colorectal cancer screening in US immigrants: a scoping review. J Surg Res 2023;282:53–64. 36257164 10.1016/j.jss.2022.08.024 PMC10369365

[21] Chan DNS , So WKW. A systematic review of the factors influencing ethnic minority women’s cervical cancer screening behavior: from intrapersonal to policy level. Cancer Nurs 2017;40:E1–30. 10.1097/NCC.0000000000000436 28081032

[22] D'Onise K , Iacobini ET, Canuto KJ. Colorectal cancer screening using faecal occult blood tests for Indigenous adults: a systematic literature review of barriers, enablers and implemented strategies. Prev Med 2020;134:106018. 32057956 10.1016/j.ypmed.2020.106018

[23] Travis E , Ashley L, Pownall M, O'Connor DB. Barriers to flexible sigmoidoscopy colorectal cancer screening in low uptake socio-demographic groups: a systematic review. Psychooncology 2020;29:1237–47. 32539187 10.1002/pon.5443

[24] Chin YH , Ng CH, Seah SHY, et al Evolving perspectives on stool testing for colorectal cancer: a qualitative systematic review. Eur J Cancer Prev 2020;29:416–23. 32740167 10.1097/CEJ.0000000000000607

[25] Dressler J , Johnsen AT, Madsen LJ, et al Factors affecting patient adherence to publicly funded colorectal cancer screening programmes: a systematic review. Public Health 2021;190:67–74. 33360029 10.1016/j.puhe.2020.10.025

[26] National Health Service (NHS). How should I check my breasts?. Available at: https://www.nhs.uk/common-health-questions/womens-health/how-should-i-check-my-breasts/ (10 July 2023, date last accessed).

[27] Andreeva VA , Pokhrel P. Breast cancer screening utilization among Eastern European immigrant women worldwide: a systematic literature review and a focus on psychosocial barriers. Psychooncology 2013;22:2664–75. 23824626 10.1002/pon.3344

[28] Jerome-D'Emilia B , Gachupin FC, Suplee PD. A systematic review of barriers and facilitators to mammography in American Indian/Alaska native women. J Transcult Nurs 2019;30:173–86. 30122121 10.1177/1043659618793706

[29] Oh KM , Taylor KL, Jacobsen KH. Breast cancer screening among Korean Americans: a systematic review. J Community Health 2017;42:324–32. 27678390 10.1007/s10900-016-0258-7

[30] Pagliarin F , Pylkkanen L, Salakari M, Deandrea S. Are women satisfied with their experience with breast cancer screening? Systematic review of the literature. Eur J Public Health 2020;31:206–14. 10.1093/eurpub/ckaa202 33200183

[31] Pariser A , Hirko KA, Muñoz GM, et al Barriers to access for cervical and breast cancer screenings among female latinx migrant farmworkers in the US: a scoping literature review. J Prim Care Community Health 2022;13:21501319211073252. 35068265 10.1177/21501319211073252 PMC8796074

[32] Kandasamy S , Jonathan Y, Majid U, et al Indigenous women’s experiences of cervical cancer screening: incorporating indigenous ways of knowing into a systematic review and meta-synthesis of qualitative research. Glob Public Health 2022;17:2676–89. 34842041 10.1080/17441692.2021.2010115

[33] Serrano B , Ibáñez R, Robles C, et al Worldwide use of HPV self-sampling for cervical cancer screening. Prev Med 2022;154:106900. 34861338 10.1016/j.ypmed.2021.106900

[34] Cudjoe J , Nkimbeng M, Turkson-Ocran RA, et al Understanding the Pap testing behaviors of african immigrant women in developed countries: a systematic review. J Immigr Minor Health 2021;23:840–56. 33165711 10.1007/s10903-020-01119-x PMC8747177

[35] Chorley AJ , Marlow LA, Forster AS, et al Experiences of cervical screening and barriers to participation in the context of an organised programme: a systematic review and thematic synthesis. Psychooncology 2017;26:161–72. 27072589 10.1002/pon.4126 PMC5324630

[36] Jillapalli R , Radhakrishnan K. Cervical cancer screening behaviors among Asian Indians in the United States: a systematic review. J Immigr Minor Health 2022;24:779–89. 34273046 10.1007/s10903-021-01237-0

[37] Majid U , Kandasamy S, Farrah K, Vanstone M. Women's preferences and experiences of cervical cancer screening in rural and remote areas: a systematic review and qualitative meta-synthesis. Rural Remote Health 2019;19:5190–11. 31640391 10.22605/RRH5190

[38] Christy K , Kandasamy S, Majid U, et al Understanding Black Women's perspectives and experiences of cervical cancer screening: a systematic review and qualitative meta-synthesis. J Health Care Poor Underserved 2021;32:1675–97. 34803036 10.1353/hpu.2021.0159

[39] Rogers CR , Mitchell JA, Franta GJ, et al Masculinity, racism, social support, and colorectal cancer screening uptake among African American men: a systematic review. Am J Mens Health 2017;11:1486–500. 26483293 10.1177/1557988315611227 PMC4835264

[40] Boland L , Kothari A, McCutcheon C, Graham ID, for the Integrated Knowledge Translation Research Network. Building an integrated knowledge translation (IKT) evidence base: colloquium proceedings and research direction. Health Res Policy Syst 2020;18:8. 31959184 10.1186/s12961-019-0521-3 PMC6972018

[41] Jull J , Giles A, Graham ID. Community-based participatory research and integrated knowledge translation: advancing the co-creation of knowledge. Am Implement Sci. 2017;12(1):150. 10.1186/s13012-017-0696-3 PMC5735911 29258551

[42] Connolly D , Hughes X, Berner A. Barriers and facilitators to cervical cancer screening among transgender men and non-binary people with a cervix: A systematic narrative review. Prev Med. 2020;135:106071. 32243938 10.1016/j.ypmed.2020.106071

[43] Ferdous M , Lee S, Goopy S, Yang H, Rumana N, Abedin T, et al Barriers to cervical cancer screening faced by immigrant women in Canada: a systematic scoping review. BMC Womens Health 2018;18(1):165. 30305056 10.1186/s12905-018-0654-5 PMC6180489

