# Supplementary material for: What factors influence the uptake of bowel, breast and cervical cancer screening? An overview of international research
Source: Eur J Public Health. 2024 May 3;34(4):818–25. doi: 10.1093/eurpub/ckae073 (PMC11293835; doi:10.1093/eurpub/ckae073)
Supplement: ckae073_Supplementary_Data [file ckae073_supplementary_data.zip › ckae073_Supplementary_Data/ejph-2023-09-om-0509-File002.pdf]

Search strategy (Ovid platform)

1.	exp "Early Detection of Cancer"/ or exp Mass Screening/
2.	((screen* or detect* or diagnos* or test*) adj5 (cancer* or neoplasm*)).tw,kf.
3.	(mammogra* or colonoscop* or "f?ecal immunochemical test" or "f?ecal test" or "pap smear" or "pap test" or "HPV" or "human papillomavirus").tw,kf.
4.	1 or 2 or 3
5.	exp Breast Neoplasms/ or exp Uterine Cervical Neoplasms/ or exp Breast Cancer Lymphedema/ or exp Colorectal Neoplasms/
6.	((breast or bowel or cervical or colo*) adj5 (cancer? or neoplasm?)).tw,kf.
7.	5 or 6
8.	(compli* or comply* or adher* or encourag* or promot* or uptake* or non-attend* or nonattend* or accept* or attend* or attitude* or utilis* or utiliz* or refus* or reluctan* or non-respon* or nonrespon* or barrier* or facilitat* or enabl* or engag* or perception* or experience* or view* or intention*).tw,kw.
9.	systematic review.pt.
10.	systematic review/ or systematic reviews as topic/
11.	(systematic$ adj5 review$).tw,kf.
12.	9 or 10 or 11
13.	4 and 7 and 8 and 12
14.	limit 13 to yr="2012 -Current"
15.	limit 14 to english language
16.	remove duplicates from 15

DOI 10.17605/OSF.IO/57B86
