# Supplementary material for: What factors influence the uptake of bowel, breast and cervical cancer screening? An overview of international research
Source: Eur J Public Health. 2024 May 3;34(4):818–25. doi: 10.1093/eurpub/ckae073 (PMC11293835; doi:10.1093/eurpub/ckae073)
Supplement: ckae073_Supplementary_Data [file ckae073_supplementary_data.zip › ckae073_Supplementary_Data/ejph-2023-09-om-0509-File003.pdf]

Data extraction form (BOWEL)

| Review details | |
|---|---|
| Study ID (First author, year) | Ait Ouakrim (2013) |
| Title of the review | Screening Participation Predictors for People at Familial Risk of Colorectal Cancer: A Systematic Review |
| Review objectives/focus of the review | A systematic review of the literature was conducted to identify observational studies that investigate factors associated with CRC screening participation among people at increased risk due to family history of the disease. The principal objective was to provide an inventory of these factors that could be used to inform future research and to target interventions to increase CRC screening uptake in this sector of the population. |
| Outcomes assessed | ☐Barriers<br>☒Facilitators - *('predictors' associated with CRC screening participation; only facilitators highlighted in key synthesis points)* |
| Type of review | A review that seeks to include:<br>☒Qualitative studies<br>☐Quantitative studies<br>☐Mixed methods studies<br>☐Not specified |
| Systematic review | Do the authors state this is a systematic review, systematic scoping review, or other form of systematic evaluation?<br>☒Yes<br>☐No |
| Screening programmes targeted by the review | ☒Bowel<br>☐Breast<br>☐Cervical |
| Focus on specific method of screening? | N/A |
| Details of included studies | |
| Population(s) of the included reviews | Included characteristics:<br>☒Age: Not consistently reported across included studies, range from ≥18 years to 79 years<br>☒Age within screening for NHS Scotland (50-74)?:<br>☐Gender and/or sex (described by the authors): Not reported – focus of the review is on people at increased risk for colorectal cancer (defined by having at least one first-degree relative affected with the disease)<br>☐Ethnicity: Not reported<br>☒Other: Eight studies referred to educational level of participants as a predictor of CRC screening - only three found an increase in screening behaviours as related to a higher level of education; one study found people living as a couple were more likely to undergo screening |
| Number of studies included | 10 |
| Total number of participants across all studies | 4,304 |
| Type of studies included | ☒Qualitative studies - 10<br>☐Quantitative studies<br>☐Mixed methods studies<br>☐Not specified |

Data extraction form (BOWEL)

| | |
|---|---|
| Geographic scope (high-income countries only) | AUS; CAN; FRA; ITA; USA |
| **Search strategy and methods** | |
| Sources | MEDLINE, Cinahl Information Sevices, Embase, and PsycINFO databases |
| Search restrictions (language, years, region etc) | The search was conducted using selected MeSH and free-text terms relating to colorectal cancer screening and familial risk; the list of terms was developed with the assistance of a trained medical librarian. |
| Search dates | Jan 1995 to May 2012 |
| Other criteria | References from all relevant articles were then checked for any additional publications. |
| Instrument/tool used for quality appraisal of studies | ☐Yes:<br>☒Not specified |
| Characteristics of included studies table? | ☒Yes: See Table 1<br>☐No |
| Method of analysis/synthesis of results | ☒Narrative synthesis<br>☐Thematic analysis<br>☒Other qualitative analysis: Use of the Preventive Health Model for psychological constructs<br>☐Meta-analysis<br>☐Other quantitative analysis |
| Presentation of results supported by | ☒Tabulation: See Tables 2 and 3<br>☐Framework/model<br>☐Forest plot<br>☐Other: |
| **Key findings** | |
| Key barriers and/or facilitators identified | Receiving recommendations from clinicians was the most consistent predictor identified across studies and appears to be a strong facilitator of CRC screening.<br><br>The current study also revealed a consistent pattern of association of predictor variables related to family influence with the familial aspect of the disease, such as the strength of family history and a person's relationship to the affected relative.<br><br>Among the psychological constructs, “social influence” emerged as the most consistent predictor of screening participation.<br><br>See: Evidence synthesis |
| Limitations of the review | – Limited number of results, with studies often failing to distinguish between screening and diagnostic tests.<br>– No uniform way to present heterogeneous material; obstacle in translating research to the clinical setting |
| Implications for future research | – Influence of clinicians shown to be key in adherence to health Implications for future research such as screening; “…any public health initiative intending to improve the level of CRC screening participation among those at increased risk due to their family history of the disease will be more effective if clinicians play a key role.” |

| | |
|---|---|
| | – Further research needed on the motives of those at increased risk of CRC versus the general population |
| Other notes | N/A |
| Other | |
| Quality appraisal (JBI checklist) | ☒Include<br>☐Exclude |

| Review details | |
|---|---|
| Study ID (First author, year) | Bromley (2015) |
| Title of the review | Explaining persistent under-use of colonoscopic cancer screening in African Americans: A systematic review |
| Review objectives/focus of the review | Although African Americans have the highest incidence and mortality from colorectal cancer (CRC), they are less likely than other racial groups to undergo CRC screening. Previous research has identified barriers to CRC screening among African Americans. However we lack a systematic review that synthesizes contributing factors and informs interventions to address persistent disparities. |
| Outcomes assessed | ☒Barriers<br>☒Facilitators |
| Type of review | A review that seeks to include:<br>☒Qualitative studies<br>☒Quantitative studies<br>☐Mixed methods studies<br>☐Not specified |
| Systematic review | Do the authors state this is a systematic review, systematic scoping review, or other form of systematic evaluation?<br>☒Yes<br>☐No |
| Screening programmes targeted by the review | ☒Bowel<br>☐Breast<br>☐Cervical |
| Focus on specific method of screening? | Colonoscopy |
| Details of included studies | |
| Population(s) of the included reviews | Included characteristics:<br>☐Age: Not reported<br>☐Age within screening for NHS Scotland (50-74)?:<br>☒Gender and/or sex (described by the authors): Two studies focused specifically on men; all other studies described as either ‘adults’, ‘participants’, ‘residents’ or ‘patients’<br>☒Ethnicity: African American<br>☒Other: Authors note the “majority of studies included low-income subjects from Community Health Centers or primary care facilities in urban areas of the US”; five studies featured some or all rural participants. Three study populations were reflective of differing healthcare providers including medical residents, family practitioners, and other specialist healthcare providers (which may include non-African Americans). |

Data extraction form (BOWEL)

| | |
|---|---|
| Number of studies included | 19 |
| Total number of participants across all studies | 978,381 (some studies noted as approximate totals) |
| Type of studies included | ☒Qualitative studies - 8<br>☒Quantitative studies - 11<br>☐Mixed methods studies<br>☐Not specified |
| Geographic scope (high-income countries only) | USA (incl some rural) |
| **Search strategy and methods** | |
| Sources | MEDLINE and the Cochrane Central Register of Controlled Trials (CENTRAL) databases |
| Search restrictions (language, years, region etc) | See Figure 1 for Medline search terms; search restricted to English language articles published between 1950 and November 2013. |
| Search dates | Not reported |
| Other criteria | N/A |
| Instrument/tool used for quality appraisal of studies | ☒Yes: Criteria for Reporting Qualitative Research (COREQ); Strengthening the Reporting of Observational Studies in Epidemiology (STROBE) guidelines<br>☐Not specified |
| Characteristics of included studies table? | ☒Yes: See Table 1 and Table 2<br>☐No |
| Method of analysis/synthesis of results | ☐Narrative synthesis<br>☐Thematic analysis<br>☒Other qualitative analysis: Creation of a conceptual framework<br>☐Meta-analysis<br>☐Other quantitative analysis |
| Presentation of results supported by | ☐Tabulation<br>☒Framework/model – conceptual model (Fig 4)<br>☐Forest plot<br>☐Other: |
| **Key findings** | |
| Key barriers and/or facilitators identified | Patient-level barriers to colonoscopy screening include fear, lack of knowledge about CRC screening, and competing personal and medical issues.<br><br>Provider-level factors include knowledge deficits of guidelines and barriers, and counselling practices including recommendations to attend screening.<br><br>System-level factors include access to care, financial barriers, and infrequent primary care visits.<br><br>See: Results, abstract |
| Limitations of the review | – Risk of publication bias (use of published literature only); review may be biased by selective reporting as primary studies may not have addresses all domains investigated or reported on key facilitators<br>– Difficult to be certain proposed solutions will ultimately mitigate screening disparities in African Americans |

| | |
|---|---|
| | – Findings may not be applicable to African Americans in higher socioeconomic standing, as majority of studies focused on low-income settings<br>– Not all barriers/facilitators identified are wholly specific to African Americans |
| Implications for future research | – Ensuring that "providers appropriately recommend CRC screening to African Americans, and evaluate whether patients fully comprehend and recall the recommendation once delivered."<br>– Confusion among choice in screening; future research "must determine whether optimal physician counselling practices are to suggest colonoscopy alone or to provide a menu of screening options."<br>– Culturally tailored interventions would better address the cumulative effects of barriers for African Americans including procedural resistance<br>– Multi-modal interventions should extend education and awareness beyond the clinic and into community settings |
| Other notes | N/A |
| Other | |
| Quality appraisal (JBI checklist) | ☒Include<br>☐Exclude |

| | |
|---|---|
| Review details | |
| Study ID (First author, year) | Chin (2020) |
| Title of the review | Evolving perspectives on stool testing for colorectal cancer: a qualitative systematic review |
| Review objectives/focus of the review | This qualitative review aims to discover the barriers and facilitators to the utility of the [faecal immunochemical test] FIT from the general population's perspective. |
| Outcomes assessed | ☒Barriers<br>☒Facilitators |
| Type of review | A review that seeks to include:<br>☒Qualitative studies<br>☐Quantitative studies<br>☐Mixed methods studies<br>☐Not specified |
| Systematic review | Do the authors state this is a systematic review, systematic scoping review, or other form of systematic evaluation?<br>☒Yes<br>☐No |
| Screening programmes targeted by the review | ☒Bowel<br>☐Breast<br>☐Cervical |
| Focus on specific method of screening? | Faecal immunochemical test (FIT) |
| Details of included studies | |
| Population(s) of the included reviews | Included characteristics:<br>☒Age: 50-75 years old |

| | |
|---|---|
| | ☒Age within screening for NHS Scotland (50-74)?: N/A<br>☐Gender and/or sex (described by the authors): Not reported<br>☒Ethnicity: One study described a target population of African Americans; one additional study focused on Latinos.<br>☒Other: One study focused on under-insured and uninsured individuals; four studies included the views of healthcare providers. |
| Number of studies included | 11 |
| Total number of participants across all studies | 432 |
| Type of studies included | ☒Qualitative studies - 11<br>☐Quantitative studies<br>☐Mixed methods studies<br>☐Not specified |
| Geographic scope (high-income countries only) | AUS; GBR; USA |
| Search strategy and methods | |
| Sources | Medline, Embase, CINAHL, PsycINFO, and Web of Science |
| Search restrictions (language, years, region etc) | Search strategy, Supplemental Digital Content 1, *http://links.lww.com/EJCP/A298* |
| Search dates | From inception till 21 December 2019 |
| Other criteria | N/A |
| Instrument/tool used for quality appraisal of studies | ☒Yes: CASP tool<br>☐Not specified |
| Characteristics of included studies table? | ☒Yes: See Table 1<br>☐No |
| Method of analysis/synthesis of results | ☐Narrative synthesis<br>☒Thematic analysis<br>☐Other qualitative analysis<br>☐Meta-analysis<br>☐Other quantitative analysis |
| Presentation of results supported by | ☒Tabulation: See Table 2<br>☐Framework/model<br>☐Forest plot<br>☐Other: |
| Key findings | |
| Key barriers and/or facilitators identified | Faecal immunochemical test kit factors: collecting stool samples is embarrassing (‘disgusting’), preferred the convenience of the FIT, preference for pictorial instructions<br><br>Patient perception of CRC screening: general lack of understanding and knowledge of FIT screening, provider did not discuss alternative methods of testing, questions surrounding accuracy and results of a FIT, fatalism, low risk perception, lack of a prevention attitude, fear of being diagnosed with cancer and future treatment, relevance of the screening as a facilitator |

| | |
|---|---|
| | Social health support system: education and publicity (communication), cost of care, normative support from friends, family and partners as both barrier and facilitator<br><br>See: Results |
| Limitations of the review | – Search scope and strategy – only included English language articles, possibility of incomplete information retrieval<br>– Findings may not be generalisable as most articles were from North America<br>– Included studies did not fully address aspects of the FIT such as protective hygiene equipment, durability of the components of the kit, and patients views on making further screening appointments with a physician |
| Implications for future research | – "Consistent effort by relevant authorities is needed to encourage the population to embrace the colorectal cancer screening experience"; this includes supplementing the use of FIT with pictorial guides, video, and mail delivery of the kit and results<br>– Further required are "strong public policies that promote and create awareness about the use of FIT" |
| Other notes | Article sourced from inter-library loan system. |
| Other | |
| Quality appraisal (JBI checklist) | ☒Include<br>☐Exclude |

| Review details | |
|---|---|
| Study ID (First author, year) | D'Onise (2020) |
| Title of the review | Colorectal cancer screening using faecal occult blood tests for Indigenous adults: A systematic literature review of barriers, enablers and implemented strategies |
| Review objectives/focus of the review | Colorectal cancer (CRC) screening using a Faecal Occult Blood Test (FOBT) is a well-established population intervention to reduce mortality and morbidity of CRC. As Indigenous people are not fully benefiting from the screening programs, a greater understanding of barriers and enablers affecting participation is needed. |
| Outcomes assessed | ☒Barriers<br>☐Facilitators *(interventions to increase uptake are discussed, but are not within the scope of the current overview)* |
| Type of review | A review that seeks to include:<br>☒Qualitative studies<br>☒Quantitative studies<br>☒Mixed methods studies<br>☐Not specified |
| Systematic review | Do the authors state this is a systematic review, systematic scoping review, or other form of systematic evaluation?<br>☒Yes<br>☐No |

Data extraction form (BOWEL)

| | |
|---|---|
| Screening programmes targeted by the review | ☒Bowel<br>☐Breast<br>☐Cervical |
| Focus on specific method of screening? | Faecal occult blood tests (FOBT) |
| **Details of included studies** | |
| Population(s) of the included reviews | Included characteristics:<br>☒Age: Age data not reported for qualitative studies; variable for quantitative studies, lowest age reported 21 years and highest age limit of 87 years<br>☒Age within screening for NHS Scotland (50-74)?:<br>☒Gender and/or sex (described by the authors): Two studies focused specifically on women; one study focused specifically on men; all others either not described or referred to as 'adults'<br>☒Ethnicity: Indigenous peoples including American Indians, Alaskan Natives, Native Hawaiians, Hopi people, Navajo people, Aboriginal Canadians, Māori and Pacific Islanders, Aboriginal and Torres Strait Islanders<br>☒Other: One study reported "greater barriers for older patients due to language barrier, strong use of traditional medicine, having problems to navigate the system, understanding education materials"; six study populations focused on health service providers and/or other community members (which may include non-Indigenous persons). |
| Number of studies included | 34 |
| Total number of participants across all studies | 109,850 (all participants, some studies noted as inclusive of non-Indigenous persons) |
| Type of studies included | ☒Qualitative studies - 16<br>☒Quantitative studies - 17<br>☒Mixed methods studies - 1<br>☐Not specified |
| Geographic scope (high-income countries only) | AUS; CAN; NSL; USA |
| **Search strategy and methods** | |
| Sources | PubMed, Embase, Sociological Abstracts, Scopus, CINAHL, and selected websites |
| Search restrictions (language, years, region etc) | See Appendix A |
| Search dates | Jan 1993 to Jan 2019 |
| Other criteria | Some grey material included; see Appendix B |
| Instrument/tool used for quality appraisal of studies | ☒Yes: Long and Godfrey (2004) and Quality Assessment tool for Quantitative studies of Effective Public Health Practice Project (EPHPP)<br>☐Not specified |
| Characteristics of included studies table? | ☒Yes: See Table 1 and Table 2<br>☐No |
| Method of analysis/synthesis of results | ☒Narrative synthesis<br>☐Thematic analysis<br>☐Other qualitative analysis |

| | |
|---|---|
| | ☐Meta-analysis<br>☐Other quantitative analysis |
| Presentation of results supported by | ☒Tabulation<br>☐Framework/model<br>☐Forest plot<br>☐Other: |
| Key findings | |
| Key barriers and/or facilitators identified | A consistently reported barrier for CRC screening is the lack of knowledge, as CRC is perceived as having low importance as it does not receive the same attention as other health conditions (such as diabetes, breast cancer)<br><br>Misconceptions about risk – CRC only affects men, or that Indigenous people do not get CRC<br><br>For many Indigenous people across the USA, New Zealand and Australia, cancer, bodily functions, and illness are taboo topics or against cultural protocol and many participants reported that CRC and CRC screening are not openly discussed in the community and can even be difficult conversations to raise with health service providers<br><br>Many Indigenous people also fear a cancer diagnosis or have fatalistic views of cancer leading to people not valuing the screening test, believing cancer is a death sentence and/or would rather not know<br><br>Common barriers identified by American, New Zealander and Australian participants were that the current CRC education resources are lacking or not culturally appropriate, use complex medical terminology and/or do not elicit action<br><br>The FOBT is often described as embarrassing, distasteful or unpleasant<br><br>Preference for and strong belief in Traditional medicines which may deter Indigenous people from seeking health care from Western medicine and therefore CRC screening<br><br>Lacking a health professional recommendation to screen for CRC was found as a barrier of Indigenous participation<br><br>Experiences of medical discrimination and cultural competency in the health services resulted in a lack of trust in the health services, health providers<br><br>See: Results |
| Limitations of the review | – Quality of included studies was generally low<br>– Limited analysis as not an international study of Indigenous people |

| | |
|---|---|
| | – Challenging to discern if FOBT was included in the study, and study results were often presented alongside colonoscopy or other procedures (not exclusively related to FOBT screening) |
| Implications for future research | – Need for culturally competent health service access, including Indigenous health service providers<br>– Intervention studies should be conducted in partnership with Indigenous communities to improve participation, "ideally featuring and delivered by Indigenous people"<br>– Health providers require additional training "to improve their knowledge of CRC and CRC screening, cultural training and generally encourage preventative healthcare".<br>– Future studies may benefit from mixed methods; low-cost interventions (community lab drop-off of FOBT kits, telephone/text interventions) could be further considered |
| Other notes | N/A |
| Other | |
| Quality appraisal (JBI checklist) | ☒Include<br>☐Exclude |

| Review details | |
|---|---|
| Study ID (First author, year) | Decruz (2021) |
| Title of the review | Afterthoughts on colonoscopy. Was it that bad? |
| Review objectives/focus of the review | Colorectal cancer is among the top three most common cancers globally. In order to reduce the health burden, it is important to improve the uptake of colorectal cancer screening by understanding the barriers and facilitators encountered. There are numerous reports in the literature on the views of the general public on cancer screening. However, the experiences of colonoscopy patients are not as well studied. This paper maps their perceptions. |
| Outcomes assessed | ☒Barriers<br>☒Facilitators |
| Type of review | A review that seeks to include:<br>☒Qualitative studies<br>☐Quantitative studies<br>☐Mixed methods studies<br>☐Not specified |
| Systematic review | Do the authors state this is a systematic review, systematic scoping review, or other form of systematic evaluation?<br>☒Yes<br>☐No |
| Screening programmes targeted by the review | ☒Bowel<br>☐Breast<br>☐Cervical |
| Focus on specific method of screening? | Colonoscopy |
| Details of included studies | |

Data extraction form (BOWEL)

| | |
|---|---|
| Population(s) of the included reviews | Included characteristics:<br>☒Age: Variable across studies, lowest range of 18 and highest range of 92<br>☒Age within screening for NHS Scotland (50-74)?:<br>☒Gender and/or sex (described by the authors): One study focused on women only; otherwise not reported<br>☒Ethnicity: One study focused on American Indian women; otherwise not reported<br>☒Other: One study focused on "people from medically underserved areas"; varying subpopulations for each of the 11 included studies, review is aiming to evaluate the overarching experiences of colonoscopy patients, not focus any one specific subgroup |
| Number of studies included | 11 |
| Total number of participants across all studies | 482 |
| Type of studies included | ☒Qualitative studies - 11<br>☐Quantitative studies<br>☐Mixed methods studies<br>☐Not specified |
| Geographic scope (high-income countries only) | AUS; DNK; GBR; USA |
| Search strategy and methods | |
| Sources | Medline, Embase, CINAHL, PsycINFO and Web of Science Core Collection |
| Search restrictions (language, years, region etc) | Full search available in Supplementary Material 1 |
| Search dates | Conducted 3 December 2019; no further details available |
| Other criteria | N/A |
| Instrument/tool used for quality appraisal of studies | ☒Yes: Critical Appraisal Skills Programme (CASP)<br>☐Not specified |
| Characteristics of included studies table? | ☒Yes: See Table 1<br>☐No |
| Method of analysis/synthesis of results | ☐Narrative synthesis<br>☒Thematic analysis<br>☐Other qualitative analysis<br>☐Meta-analysis<br>☐Other quantitative analysis |
| Presentation of results supported by | ☐Tabulation<br>☐Framework/model<br>☐Forest plot<br>☒Other: Narrative only |
| Key findings | |
| Key barriers and/or facilitators identified | Pre-procedure: bowel preparation is disruptive (test preparation difficulties), preference for FIT test to circumvent bowel preparation, lack of tailored information strategies surrounding best screening practices vs clear communication of the process, normative support or lack thereof from family and friends |

Data extraction form (BOWEL)

| | |
|---|---|
| | During the procedure: Pain, use of sedative allows for a fast/easy experience, emotional distress (humiliation, embarassment…), interpersonal skills of healthcare providers (rapport with providers during the procedure)<br><br>Post-procedure: Quick release of results, often within recovery from sedation, ability to discuss further with healthcare staff<br><br>See: Results |
| Limitations of the review | – Articles limited to English Language and largely reflective of North American and UK settings; may not be fully generalisable to other global areas as a result |
| Implications for future research | – "An overall need is identified for a holistic approach to improve colonoscopy adherence rates"; this includes patient comfort, the use of clear instructional aids including graphics, the importance of patient rapport, and individualized options for procedural steps and use of sedation |
| Other notes | N/A |
| Other | |
| Quality appraisal (JBI checklist) | ☒Include<br>☐Exclude |

| | |
|---|---|
| Review details | |
| Study ID (First author, year) | Dressler (2021) |
| Title of the review | Factors affecting patient adherence to publicly funded colorectal cancer screening programmes: a systematic review |
| Review objectives/focus of the review | The aim of the study was to identify barriers, facilitators and modifiers to participation in systematised, stool sample–based, publicly financed CRC screening programmes. |
| Outcomes assessed | ☒Barriers<br>☒Facilitators |
| Type of review | A review that seeks to include:<br>☒Qualitative studies<br>☒Quantitative studies<br>☐Mixed methods studies<br>☐Not specified |
| Systematic review | Do the authors state this is a systematic review, systematic scoping review, or other form of systematic evaluation?<br>☒Yes<br>☐No |
| Screening programmes targeted by the review | ☒Bowel<br>☐Breast<br>☐Cervical |
| Focus on specific method of screening? | Systematised, stool sample–based, publicly financed CRC screening programmes; included studies further defined by type and frequency of testing including FOBT, iFOBT and gFOBT |
| Details of included studies | |
| Population(s) of the included reviews | Included characteristics:<br>☒Age: Variable by study; studies reporting on age groups less |

| | |
|---|---|
| | than 40 years or more than 80 years were excluded to improve homogeneity of the populations<br>☒Age within screening for NHS Scotland (50-74)?:<br>☒Gender and/or sex (described by the authors): Female and male; sex not reported for one study<br>☐Ethnicity: Not reported<br>☒Other: Authors note that both sociodemographic and socioeconomic factors on screening participation were excluded as "these have already been investigated thoroughly" (see: study selection). |
| Number of studies included | 21 |
| Total number of participants across all studies | 31,039 |
| Type of studies included | ☒Qualitative studies - 11<br>☒Quantitative studies - 10<br>☐Mixed methods studies<br>☐Not specified |
| Geographic scope (high-income countries only) | AUS; CAN; DNK; ESP; FRA; GBR; IRL; ITA; NLD |
| Search strategy and methods | |
| Sources | PubMed, Embase, MEDLINE, CINAHL, Cochrane CENTRAL and PsycINFO |
| Search restrictions (language, years, region etc) | No limitation was set on study design or language. The search strategy had a combination of synonyms of the keywords including 'colorectal cancer', 'screening', 'faecal immunochemical test', 'adherence' and 'participation'. The search strategy and filters were modified for each database. |
| Search dates | The search included studies published since the commencement of the respective databases until March 5, 2018. |
| Other criteria | A search of grey literature was performed in Google Scholar. Reference lists of the included articles were screened for additional relevant articles. |
| Instrument/tool used for quality appraisal of studies | ☒Yes: CASP tools for qualitative and quantitative studies<br>☐Not specified |
| Characteristics of included studies table? | ☒Yes: See Table 1 and Table 2<br>☐No |
| Method of analysis/synthesis of results | ☐Narrative synthesis<br>☒Thematic analysis<br>☐Other qualitative analysis<br>☐Meta-analysis<br>☐Other quantitative analysis |
| Presentation of results supported by | ☒Tabulation<br>☐Framework/model<br>☐Forest plot<br>☒Other: Narrative summary technique |
| Key findings | |
| Key barriers and/or facilitators identified | Key themes:<br>Psychology – indifferent about taking the test/procrastination, embarrassment/disgust towards the test, lack of knowledge |

| | |
|---|---|
| | about cancer and symptoms, fear, other mental or emotional problems<br><br>Religion – fatalism<br><br>Logistics – time constraints, forgetfulness, other priorities; technical problems with self-testing kits or not receiving an invitation to screen<br><br>Health-related issues – other competing medical issues including physical problems, functional limitations, mental health etc<br><br>Knowledge and awareness -lack of relevance of screening, lack of knowledge about the screening options and outcomes, low health literacy<br><br>General practitioners – involvement of GP perceived as an important facilitator<br><br>Environmental factors – friends or partner participating in screening, encouragement by other networks, previous positive screening experience, knowing someone diagnosed with cancer<br><br>See: Results |
| Limitations of the review | – Non-participation was generally not caused by a negative attitude towards CRC screening. It is likely that this finding might represent selection bias as people participating in studies of barriers of screening are more prone to screening in general.<br>– Moreover, the inclusion of both qualitative and quantitative studies on an equal basis was a discipline lacking international consensus; a barrier could be reported because it was mentioned by a few patient in an interview or reported by most participants in a large questionnaire survey.<br>– Excluded sociodemographic variables; further research is needed in regards to publicly funded FOBT-based screening programmes within the EU |
| Implications for future research | – "Involvement of general practitioners, implementation of media campaigns and the creation of a logistical support unit can result in higher participation rates" |
| Other notes | N/A |
| Other | |
| Quality appraisal (JBI checklist) | ☒Include<br>☐Exclude |

| Review details | |
|---|---|
| Study ID (First author, year) | Kerrison (2021) |
| Title of the review | Patient barriers and facilitators of colonoscopy use: A rapid systematic review and thematic synthesis of the qualitative literature |

Data extraction form (BOWEL)

| | |
|---|---|
| Review objectives/focus of the review | The aim of this review was to characterise the barriers and facilitators of colonoscopy use described in the qualitative literature. |
| Outcomes assessed | ☒Barriers<br>☒Facilitators |
| Type of review | A review that seeks to include:<br>☒Qualitative studies<br>☐Quantitative studies<br>☐Mixed methods studies<br>☐Not specified |
| Systematic review | Do the authors state this is a systematic review, systematic scoping review, or other form of systematic evaluation?<br>☒Yes<br>☐No |
| Screening programmes targeted by the review | ☒Bowel<br>☐Breast<br>☐Cervical |
| Focus on specific method of screening? | Colonoscopy |
| Details of included studies | |
| Population(s) of the included reviews | Included characteristics:<br>☒Age: Variable by study when reported, age range from 23 to 90<br>☒Age within screening for NHS Scotland (50-74)?:<br>☒Gender and/or sex (described by the authors): Men (male) and women (female)<br>☒Ethnicity: Most studies (23/57) reported a mix of ethnicities within cited populations<br>☒Other: One study "found evidence for a significant association between reporting cost as a barrier and increased socioeconomic deprivation" |
| Number of studies included | 57 |
| Total number of participants across all studies | 3,595 |
| Type of studies included | ☒Qualitative studies – 57<br>☐Quantitative studies<br>☐Mixed methods studies<br>☐Not specified |
| Geographic scope (high-income countries only) | CAN; DNK; NZL; SGP; USA |
| Search strategy and methods | |
| Sources | PubMed and PsychInfo |
| Search restrictions (language, years, region etc) | See Table 1 |
| Search dates | All searches were performed in April 2020; no further details provided. |
| Other criteria | Approach was to successively broaden the search terms until newly identified papers potentially eligible on abstract review was < 1% of the total papers found by the search. |

Data extraction form (BOWEL)

| | |
|---|---|
| Instrument/tool used for quality appraisal of studies | ☐Yes:<br>☒Not specified – authors note in the paper limitations that no formal quality assessment was performed. |
| Characteristics of included studies table? | ☒Yes: See Table 3 and Table 4<br>☐No |
| Method of analysis/synthesis of results | ☐Narrative synthesis<br>☒Thematic analysis<br>☐Other qualitative analysis<br>☐Meta-analysis<br>☐Other quantitative analysis |
| Presentation of results supported by | ☒Tabulation<br>☐Framework/model<br>☐Forest plot<br>☒Other: Tree diagram showing the relationships between key themes; see Figure 2 |
| Key findings | |
| Key barriers and/or facilitators identified | Social factors – power of positive relations, social networks and other influences; cultural taboos and perceptions of masculinity; past experiences and experiences of important others<br><br>Practical factors – competing priorities and accessibility to attend screening issues<br><br>Psychological factors – concerns about the procedure (physical and psychological), enhanced peace of mind, knowledge about CRC and screening (barrier and facilitator), attitudes towards health (lack of interest, proactive desire to stay healthy), perceived risk and mortality, post-hoc rationalisation of abnormal screening results<br><br>See: Discussion, as supported by Table 5 |
| Limitations of the review | – No formal quality assessment undertaken; search strategy used was not comprehensive, with possibility of key literature missed due to searching technique |
| Implications for future research | – Further research is needed on the barriers and facilitators specific to surveillance and follow-up colonoscopy<br>– A larger global focus is needed, as the majority of studies focused on the United States where screening programme delivery may differ from countries with socialized medicine<br>– Interventions should focus on targets amenable to change – “e.g., ‘lack of understand that bowel cancer can be an asymptomatic disease” |
| Other notes | N/A |
| Other | |
| Quality appraisal (JBI checklist) | ☒Include<br>☐Exclude |

| | |
|---|---|
| Review details | |
| Study ID (First author, year) | Kim (2018) |

Data extraction form (BOWEL)

| | |
|---|---|
| Title of the review | Unraveling the Determinants to Colorectal Cancer Screening Among Asian Americans: a Systematic Literature Review |
| Review objectives/focus of the review | Examination of facilitators and barriers to cancer screening among AA subgroups is fairly recent and the synthesis of this information is limited. As such, a systematic review was conducted examining the facilitators and the barriers among Chinese, Filipino, Korean, and Japanese Americans using a systematic literature review method. |
| Outcomes assessed | ☒Barriers<br>☒Facilitators |
| Type of review | A review that seeks to include:<br>☒Qualitative studies<br>☒Quantitative studies<br>☐Mixed methods studies<br>☐Not specified |
| Systematic review | Do the authors state this is a systematic review, systematic scoping review, or other form of systematic evaluation?<br>☒Yes<br>☐No |
| Screening programmes targeted by the review | ☒Bowel<br>☐Breast<br>☐Cervical |
| Focus on specific method of screening? | N/A |
| Details of included studies | |
| Population(s) of the included reviews | Included characteristics:<br>☒Age: The authors note the "majority of the age range in the studies was in compliance with the USPSTF-recommended age group for screening 50-75"<br>☒Age within screening for NHS Scotland (50-74)?:<br>☒Gender and/or sex (described by the authors): Men and women; three studies focused specifically on women; two studies did not report the gender breakdown of their samples<br>☒Ethnicity: Chinese, Filipino, Korean and Japanese Americans (Asian Americans)<br>☒Other: Three studies focused on lower-income AAs and two on higher income AAs, although the authors did not provide further analysis of these findings; one study examined the role of informal social support. |
| Number of studies included | 29 |
| Total number of participants across all studies | 54,645 |
| Type of studies included | ☒Qualitative studies – see Table 1 for further details on included studies as total number of qualitative and quantitative studies are unclear from study design information provided<br>☒Quantitative studies<br>☐Mixed methods studies<br>☐Not specified |
| Geographic scope (high-income countries only) | USA |

Data extraction form (BOWEL)

| Search strategy and methods | |
|---|---|
| Sources | OneSearchManoa, three EBSCO databases: Academic Search Complete, Cumulative Index to Nursing and Allied Health (CINAHL), and Psychology and Behavioral Sciences Collection, and the American Psychological Association's PsycNet |
| Search restrictions (language, years, region etc) | – Search string "colorectal cancer AND screening AND [Chinese (CA), Filipino (FA), Korean (KA), and Japanese (JA)] American." <br> – Each ethnic subgroup was searched independently from the other subgroups in each of the databases. The USPSTF CRC screening recommendation guideline was used as a guide in this review; however, age range was not restricted to USPSTF's recommended age range in order to maximize the publications included in this review for this hard to reach population. |
| Search dates | The search was conducted in August 2016. No further details on years searched was provided under methodology. |
| Other criteria | N/A |
| Instrument/tool used for quality appraisal of studies | ☐Yes: <br> ☒Not specified |
| Characteristics of included studies table? | ☒Yes: See Table 1 <br> ☐No |
| Method of analysis/synthesis of results | ☐Narrative synthesis <br> ☐Thematic analysis <br> ☒Other qualitative analysis: Use of the Health Belief Model as a primary theoretical framework to organize and synthesize the facilitators/barriers <br> ☐Meta-analysis <br> ☐Other quantitative analysis |
| Presentation of results supported by | ☒Tabulation <br> ☐Framework/model <br> ☐Forest plot <br> ☐Other: |
| Key findings | |
| Key barriers and/or facilitators identified | Aggregated key barriers/facilitators among Asian Americans: <br> – Physician recommendation was a cue to action that consistently facilitated CCSP across the subgroups. <br> – Individual's unawareness of screening tests and those stating having no problems/symptoms of CRC were also identified as a barrier to CCSP across the subgroups. <br> – Interventions targeting education on CRC screening among AA may be more effective with the inclusion of recruiting culturally appropriate non-traditional health sources as trusted and effective health information disseminators. <br> See: Discussion |
| Limitations of the review | – Findings generalisable with caution: eligible studies published in other database may have been overlooked <br> – Heterogenous data collection methods and analyses raise susceptibility to response bias |
| Implications for future research | – A "one-size-fits-all" approach will not be effective and tailored approaches that address cultural, psychological, healthcare- |

| | |
|---|---|
| | related barriers and cues to action are needed for differing Asian American ethnic subgroups<br>– Further research is needed "on the influence of ethnic culture's behavioral norm"; this may lead to better understanding as to the role of family and friends in promoting screening programmes |
| Other notes | N/A |
| Other | |
| Quality appraisal (JBI checklist) | ☒Include<br>☐Exclude |

| Review details | |
|---|---|
| Study ID (First author, year) | McLachlan (2012) |
| Title of the review | Patients' experiences and reported barriers to colonoscopy in the screening context—A systematic review of the literature |
| Review objectives/focus of the review | A systematic review of the literature was conducted to characterise patients' own experience of colonoscopy in the screening context. |
| Outcomes assessed | ☒Barriers<br>☒Facilitators *('incentives' to uptake of screening are presented)* |
| Type of review | A review that seeks to include:<br>☒Qualitative studies<br>☒Quantitative studies<br>☐Mixed methods studies<br>☐Not specified |
| Systematic review | Do the authors state this is a systematic review, systematic scoping review, or other form of systematic evaluation?<br>☒Yes<br>☐No |
| Screening programmes targeted by the review | ☒Bowel<br>☐Breast<br>☐Cervical |
| Focus on specific method of screening? | Colonscopy |
| Details of included studies | |
| Population(s) of the included reviews | Included characteristics:<br>☒Age: Inconsistently reported across included studies; noted as ranging from greater than 30 years of age to 90 years of age<br>☒Age within screening for NHS Scotland (50-74)?:<br>☒Gender and/or sex (described by the authors): Women and men, or otherwise noted as 'individuals' or population sample<br>☒Ethnicity: Variable by study when reported<br>☒Other: Three studies focused on low-income or low-income housing settings, all in the USA. Varying subpopulations for each of the 56 included studies, including those deemed at high risk or first-degree relatives of colorectal cancer patients |
| Number of studies included | 56 |
| Total number of participants across all studies | 51,454 |

Data extraction form (BOWEL)

| | |
|---|---|
| Type of studies included | ☒Qualitative studies - 11<br>☒Quantitative studies – 45<br>☐Mixed methods studies<br>☐Not specified |
| Geographic scope (high-income countries only) | AUS; CAN; GBR; HKG; NLD; USA (incl some rural) |
| **Search strategy and methods** | |
| Sources | USA (incl some rural); GBR; CAN; HKG; AUS; NLD |
| Search restrictions (language, years, region etc) | A combination of text terms and MeSH terms was used to maximise the amount of literature retrieved. The full search strategy is available on request from the authors. |
| Search dates | These searches were conducted in June 2009 and updated in June 2010 and focused on studies published in the last 13 years (1996–2009), as during this period use of colonoscopy became more common in the screening context. |
| Other criteria | In addition reference lists contained within selected published papers were also scrutinized. |
| Instrument/tool used for quality appraisal of studies | ☒Yes: Critical Appraisal Skills Programme (CASP) tools<br>☐Not specified |
| Characteristics of included studies table? | ☒Yes: See Table 1 and Table 2<br>☐No |
| Method of analysis/synthesis of results | ☒Narrative synthesis<br>☒Thematic analysis<br>☐Other qualitative analysis<br>☐Meta-analysis<br>☐Other quantitative analysis |
| Presentation of results supported by | ☒Tabulation: See Table 1 and Table 2<br>☐Framework/model<br>☐Forest plot<br>☒Other: Narrative synthesis with thematic analysis |
| **Key findings** | |
| Key barriers and/or facilitators identified | Themes identified by the authors as emerging from the literature:<br><br>A primary concern of the patients (regardless of whether they had direct experience of a colonoscopy or whether they were considering it) was discomfort and inconvenience of the laxative bowel preparation. A significant majority judged this to be the worst part of the process, and the main barrier.<br><br>Other procedure related and personal concerns were pre-test anxiety, worry about anticipated pain and potential complications. Embarrassment and feelings of vulnerability were reported particularly by women.<br><br>Inadequate knowledge about screening and CRC were common and acted as obstacles to the uptake of screening colonoscopy, as did fear of finding cancer.<br><br>Physician recommendation ranked highly as a factor influencing uptake of colonoscopic screening. |

Data extraction form (BOWEL)

| | |
|---|---|
| | See: Discussion |
| Limitations of the review | – Heterogenous results prohibit a meta-analysis; use of differing methods to derive patient reported information |
| Implications for future research | – "…increased emphasis needs to be placed on efforts to improve the bowel preparation process, enhancing comfort and modesty during the examination and identifying patients with significant anxiety beforehand".<br>– Physicians "should be aware of the impact of their endorsement and be encouraged to discuss colon cancer screening and convey its importance to patients". |
| Other notes | N/A |
| Other | |
| Quality appraisal (JBI checklist) | ☒Include<br>☐Exclude |

| Review details | |
|---|---|
| Study ID (First author, year) | Puli (2023) |
| Title of the review | Barriers to Colorectal Cancer Screening in US Immigrants: A Scoping Review |
| Review objectives/focus of the review | Timely colorectal cancer (CRC) screening has been shown to improve CRC-related morbidity and mortality rates. However, even with this preventative care tool, CRC screening rates remain below 70% among eligible United States (US) adults, with even lower rates among US immigrants. The aim of this scoping review is to describe the barriers to CRC screening faced by this unique and growing immigrant population and discuss possible interventions to improve screening. |
| Outcomes assessed | ☒Barriers<br>☐Facilitators |
| Type of review | A review that seeks to include:<br>☒Qualitative studies<br>☒Quantitative studies<br>☐Mixed methods studies<br>☐Not specified |
| Systematic review | Do the authors state this is a systematic review, systematic scoping review, or other form of systematic evaluation?<br>☒Yes<br>☐No |
| Screening programmes targeted by the review | ☒Bowel<br>☐Breast<br>☐Cervical |
| Focus on specific method of screening? | Studies further divided by screening test (FOBT, FIT, sigmoidoscopy, proctoscopy, endoscopy, and/or colonoscopy) |
| Details of included studies | |
| Population(s) of the included reviews | Included characteristics:<br>☒Age: Variable when reported; range from 18 years of age to 88 years of age ("or older") |

| | |
|---|---|
| | ☒Age within screening for NHS Scotland (50-74)?:<br>☒Gender and/or sex (described by the authors): Male and female when reported; otherwise described as ‘immigrants’<br>☒Ethnicity: Ethnicities highlighted by the authors include South Asians, Filipinos, Vietnamese, Koreans, Bangladeshi, Hispanics, Latino(x), Somali, Jamaicans, Chinese and “many more […] with most studies focused on immigrants from East and Southeast Asia”<br>☒Other: Immigrants |
| Number of studies included | 55 |
| Total number of participants across all studies | 318,939; participant numbers not reported for 6 studies |
| Type of studies included | ☒Qualitative studies – see Table 1 for further breakdown of study types<br>☒Quantitative studies<br>☐Mixed methods studies<br>☐Not specified |
| Geographic scope (high-income countries only) | USA |
| Search strategy and methods | |
| Sources | Ovid MEDLINE, EBSCOhost CINAHL, Elsevier Embase, and Clarivate Web of Science |
| Search restrictions (language, years, region etc) | See Supplementary file 1 for the complete search strategy |
| Search dates | The initial literature search was conducted on January 15, 2020 with an updated, follow-up second search on January 14, 2022. Years searched were not reported in the methodology. |
| Other criteria | N/A |
| Instrument/tool used for quality appraisal of studies | ☐Yes:<br>☒Not specified |
| Characteristics of included studies table? | ☒Yes: See Table 1<br>☐No |
| Method of analysis/synthesis of results | ☐Narrative synthesis<br>☒Thematic analysis<br>☐Other qualitative analysis<br>☐Meta-analysis<br>☐Other quantitative analysis |
| Presentation of results supported by | ☒Tabulation: See Table 2<br>☐Framework/model<br>☐Forest plot<br>☐Other: |
| Key findings | |
| Key barriers and/or facilitators identified | Commonly cited knowledge barriers included an unfamiliarity with CRC and screening, and a lack of physician recommendation—all of which restrict the ability of immigrants to navigate the healthcare system and learn about resources available to them. Access-related barriers included financial burden and lack of primary care. Collectively, these identified barriers help explain the low rates of CRC screening in immigrant population. |

Data extraction form (BOWEL)

| | |
|---|---|
| | See: Discussion (note: Table 2 provides further detailed information under specific themes, also integrating sociodemographic details which are not within the scope of the current overview) |
| Limitations of the review | – Only reflective of English language studies; perspectives from within immigrant communities, which could point to more culturally sensitive and accurate depictions of barriers faced, may have been missed.<br>– Many studies aggregated patient populations into general immigrant categories that lacked a distinction on immigrant group differences; distinct barriers require disaggregating demographic data.<br>– Potential for bias in extracted themes; attempted to mitigate this through multiple-reviewer abstraction methodology to ensure consistency in themes extracted. |
| Implications for future research | – "When designing interventions to increase screening uptake among immigrants, gaps in physician and screening education, access to care, and trust need to be addressed through culturally sensitive supports."<br>– "These interventions should be tailored to the specific immigrant group, since a one-size-fits approach fails to consider the heterogeneity within this population." |
| Other notes | N/A |
| Other | |
| Quality appraisal (JBI checklist) | ☒Include<br>☐Exclude |

| Review details | |
|---|---|
| Study ID (First author, year) | Rogers (2015) |
| Title of the review | Factors Associated with Colorectal Cancer Screening among Younger African American Men: A Systematic Review |
| Review objectives/focus of the review | To assess whether the factors influencing young adult African American men's screening intentions and behaviors are changeable through structured health education interventions, we conducted a systematic review, with the two-fold purpose of: (1) synthesizing studies examining African American men's knowledge, beliefs, and behaviors regarding CRCS; and (2) assessing these studies' methodological quality. |
| Outcomes assessed | ☒Barriers<br>☐Facilitators |
| Type of review | A review that seeks to include:<br>☒Qualitative studies<br>☒Quantitative studies<br>☐Mixed methods studies<br>☐Not specified |
| Systematic review | Do the authors state this is a systematic review, systematic scoping review, or other form of systematic evaluation? |

Data extraction form (BOWEL)

| | |
|---|---|
| | ☒Yes<br>☐No |
| Screening programmes targeted by the review | ☒Bowel<br>☐Breast<br>☐Cervical |
| Focus on specific method of screening? | N/A |
| Details of included studies | |
| Population(s) of the included reviews | Included characteristics:<br>☒Age: Age not specifically reported for all studies; some mentions within narrative, inclusion criteria notes studies must have samples including AA men younger than 50<br>☒Age within screening for NHS Scotland (50-74)?: Samples as described also incorporate <50 years of age<br>☒Gender and/or sex (described by the authors): Men<br>☒Ethnicity: African American<br>☐Other: |
| Number of studies included | 28 |
| Total number of participants across all studies | Not reported |
| Type of studies included | ☒Qualitative studies – see Table 3 for further design details as summarised by the authors<br>☒Quantitative studies<br>☐Mixed methods studies<br>☐Not specified |
| Geographic scope (high-income countries only) | USA |
| Search strategy and methods | |
| Sources | Cinahl, Embase, Medline, and PsycInfo |
| Search restrictions (language, years, region etc) | MeSH and key terms included colorectal neoplasms, colonoscopy, sigmoidoscopy, occult blood; mass screening, and AA or Black. |
| Search dates | Inclusion criteria for studies published between Jan 2000 and Feb 2013; exact dates that searches were conducted have not been provided. |
| Other criteria | Using the Scopus database, we also assessed the cited references from each of the studies included in the review. |
| Instrument/tool used for quality appraisal of studies | ☒Yes: MQS (Lee et al, 2002)<br>☐Not specified |
| Characteristics of included studies table? | ☒Yes: See Table 2 and Table 3<br>☐No: |
| Method of analysis/synthesis of results | ☒Narrative synthesis<br>☐Thematic analysis<br>☐Other qualitative analysis<br>☐Meta-analysis<br>☐Other quantitative analysis |
| Presentation of results supported by | ☒Tabulation<br>☐Framework/model<br>☐Forest plot |

| | |
|---|---|
| | ☒Other: Primarily narrative synthesis, supported by some additional tabulation |
| Key findings | |
| Key barriers and/or facilitators identified | This review identified 6 key factors associated with CRC and CRCS. These 6 factors included: previous CRCS (screening history), CRC test preference, perceived benefits, perceived barriers, CRC and CRCS knowledge, and physician support/recommendation.<br><br>Previous screening history – facilitator<br>Lack of knowledge around CRC - barrier<br>Overestimate the risk of complications from colonoscopy – barrier<br>Physician recommendation – facilitator<br>Fear of the unknown – illness or diagnosis, barrier<br>Fear of procedural pain/discomfort – barrier<br><br>See: Discussion |
| Limitations of the review | – One limitation is a weakness inherent in nearly all systematic literature reviews and meta-analyses: the possibility of having missed one or more relevant studies/reports.<br>– Another limitation is the lack of validation of the MQS criteria used in this study, and its bias towards quantitative studies. |
| Implications for future research | – Focus on the role of communication (between health promoters and lay public, providers and patients, scientists and practitioners); "Specifically supported in our findings is the suggestion medical providers capitalize on their influence and join policy makers in efforts to eliminate CRCS disparities among AA men" |
| Other notes | N/A |
| Other | |
| Quality appraisal (JBI checklist) | ☒Include<br>☐Exclude |

| | |
|---|---|
| Review details | |
| Study ID (First author, year) | Rogers (2017) |
| Title of the review | Masculinity, Racism, Social Support, and Colorectal Cancer Screening Uptake Among African American Men: A Systematic Review |
| Review objectives/focus of the review | Colorectal cancer (CRC) is highly preventable when CRC screening is utilized, yet CRC screening completion among African American men is relatively low and their mortality rates remain 50% higher juxtaposed to their White counterparts. Since a growing body of literature indicates masculinity, racism, and social support each have strong influences on CRC screening uptake, this systematic review examined the connections between these three sociocultural factors and CRC screening uptake among African American men. |
| Outcomes assessed | ☒Barriers<br>☒Facilitators |
| Type of review | A review that seeks to include:<br>☒Qualitative studies |

| | |
|---|---|
| | ☒Quantitative studies<br>☐Mixed methods studies<br>☐Not specified |
| Systematic review | Do the authors state this is a systematic review, systematic scoping review, or other form of systematic evaluation?<br>☒Yes<br>☐No |
| Screening programmes targeted by the review | ☒Bowel<br>☐Breast<br>☐Cervical |
| Focus on specific method of screening? | N/A |
| Details of included studies | |
| Population(s) of the included reviews | Included characteristics:<br>☐Age: Not reported<br>☐Age within screening for NHS Scotland (50-74)?: N/A<br>☒Gender and/or sex (described by the authors): Men<br>☒Ethnicity: African American<br>☒Other: Social support was the most frequently examined factor influencing CRC screening among African American men, reported by 16 reviewed studies |
| Number of studies included | 19 |
| Total number of participants across all studies | Not reported |
| Type of studies included | ☒Qualitative studies – See Table 2 for further design details as summarised by the authors<br>☒Quantitative studies<br>☐Mixed methods studies<br>☐Not specified |
| Geographic scope (high-income countries only) | USA |
| Search strategy and methods | |
| Sources | MEDLINE, CINAHL, EMBASE, and PsycINFO |
| Search restrictions (language, years, region etc) | See appendix for full search strategy |
| Search dates | Studies published between Jan 2000 and June 2014; exact dates that searches were conducted have not been provided. |
| Other criteria | Using Scopus, the largest bibliographic database of peer-reviewed research literature, cited reference searching for the final sample was employed to identify and assess additional studies for inclusion. |
| Instrument/tool used for quality appraisal of studies | ☒Yes: MQS (Lee et al, 2002)<br>☐Not specified |
| Characteristics of included studies table? | ☒Yes: See Tables 2 and 3<br>☐No |
| Method of analysis/synthesis of results | ☒Narrative synthesis – examines three key sociocultural factors<br>☐Thematic analysis<br>☐Other qualitative analysis |

Data extraction form (BOWEL)

| | |
|---|---|
| | ☐Meta-analysis<br>☐Other quantitative analysis |
| Presentation of results supported by | ☒Tabulation: See Tables 2 and 3 to further support narrative synthesis<br>☐Framework/model<br>☐Forest plot<br>☐Other: |
| Key findings | |
| Key barriers and/or facilitators identified | Masculinity – feelings of embarrassment about engaging in any medical exam that involved the rectum<br><br>Racism – participants who reported low perceived discrimination were more likely to report a higher level of trust in their physicians; no clear conclusions on correlation between racism and motivation to attend CRC screening<br><br>Social support – normative support provided by family members or social networks and support from healthcare providers was a key driving facilitator across a majority of studies<br><br>See: Results |
| Limitations of the review | – Sample sizes of AA men within included studies may not be adequate for corresponding methods used and data analyses undertaken.<br>– Lack of validation of MQS criteria and bias towards quantitative studies.<br>– Search parameters could lead to missing studies and risk of bias. |
| Implications for future research | – "It is important to acknowledge the need for both increased rigor and diversity in designing future research studies that will provide the highest quality of data to support practitioners, patients, policymakers, and scientific stakeholders."<br>– "…future studies might consider a mixed-methods research design to capture the depth and breadth of health care experiences among diverse subsets of African American men, particularly those with fewer socioeconomic resources, to more comprehensively understand the intersection of race and CRC screening access and completion."<br>– More research is needed in the areas of masculinity and sexuality, to further dispel homophobia associated with CRC screening |
| Other notes | N/A |
| Other | |
| Quality appraisal (JBI checklist) | ☒Include<br>☐Exclude |

| | |
|---|---|
| Review details | |
| Study ID (First author, year) | Tan (2018) |

Data extraction form (BOWEL)

| | |
|---|---|
| Title of the review | Uncovering the barriers to undergoing screening among first degree relatives of colorectal cancer patients: a review of qualitative literature |
| Review objectives/focus of the review | First degree relatives (FDRs) of colorectal cancer (CRC) patients are at higher risks of developing the disease, but screening rates amongst this group remains dismal. We undertook a systematic review of qualitative studies to identify the barriers surrounding CRC screening among FDRs from both the FDRs' and the healthcare professionals' perspectives. |
| Outcomes assessed | ☒Barriers<br>☐Facilitators |
| Type of review | A review that seeks to include:<br>☒Qualitative studies<br>☐Quantitative studies<br>☐Mixed methods studies<br>☐Not specified |
| Systematic review | Do the authors state this is a systematic review, systematic scoping review, or other form of systematic evaluation?<br>☒Yes<br>☐No |
| Screening programmes targeted by the review | ☒Bowel<br>☐Breast<br>☐Cervical |
| Focus on specific method of screening? | N/A |
| Details of included studies | |
| Population(s) of the included reviews | Included characteristics:<br>☐Age: Not reported<br>☐Age within screening for NHS Scotland (50-74)?: N/A<br>☐Gender and/or sex (described by the authors): Not reported<br>☐Ethnicity: Not reported<br>☒Other: All first-degree relatives of colorectal cancer patients; three studies also reflected health professionals including gastroenterologists, oncologists and surgeons |
| Number of studies included | 8 |
| Total number of participants across all studies | 439; includes first degree relatives, current patients, and health professionals |
| Type of studies included | ☒Qualitative studies - 8<br>☐Quantitative studies<br>☐Mixed methods studies<br>☐Not specified |
| Geographic scope (high-income countries only) | FRA; GBR; USA |
| Search strategy and methods | |
| Sources | PubMed, Cumulative Index to Nursing and Allied Health Literature (CINAHL), SCOPUS, and PsycINFO |
| Search restrictions (language, years, region etc) | The concepts searched were pertinent to the topics of CRCs, family members and screening. We used MeSH headings and free text key words combined with Boolean operators. |

Data extraction form (BOWEL)

| | |
|---|---|
| Search dates | Literature published from Jan 2000 to Feb 2017; exact dates that searches were conducted have not been provided. |
| Other criteria | This search was supplemented with manual searches of the reference lists of extracted articles. |
| Instrument/tool used for quality appraisal of studies | ☒Yes: COREQ checklist (Tong et al)<br>☐Not specified |
| Characteristics of included studies table? | ☒Yes: see Table 1<br>☐No |
| Method of analysis/synthesis of results | ☐Narrative synthesis<br>☒Thematic analysis<br>☐Other qualitative analysis<br>☐Meta-analysis<br>☐Other quantitative analysis |
| Presentation of results supported by | ☐Tabulation<br>☐Framework/model<br>☐Forest plot<br>☒Other: Narrative thematic overview |
| Key findings | |
| Key barriers and/or facilitators identified | Thematic barriers identified from FDRs:<br>Fear of diagnosis of cancer<br>Negative attitude towards screening tests (uncomfortable, embarrassed undergoing invasive procedure)<br>Lack of risk awareness as a first-degree relative<br>Locus of control/fatalism<br>Cultural factors<br><br>See: Results |
| Limitations of the review | – Search process may not be inclusive of all relevant studies; interpretation of subsequent themes may have been influenced by the personal views of the reviewers. |
| Implications for future research | – Findings from the review should be further validated outside of the context of Europe and the United States<br>– "One of our recommendations is to explore the possibility of actively engaging patients to be advocates for CRC screening amongst their family members"<br>– "The various healthcare providers must also play an active role in identifying opportunities to relate the important message of CRC screening to the patients or directly to FDRs." |
| Other notes | This review also includes thematic barriers from the perspectives of healthcare providers <u>relevant to their ability to provide care/counsel to FDRS</u>; these elements have not been included as they are not within the scope of the current review. |
| Other | |
| Quality appraisal (JBI checklist) | ☒Include<br>☐Exclude |

| Review details | |
|---|---|
| Study ID (First author, year) | Travis (2020) |

Data extraction form (BOWEL)

| | |
|---|---|
| Title of the review | Barriers to flexible sigmoidoscopy colorectal cancer screening in low uptake socio-demographic groups: A systematic review |
| Review objectives/focus of the review | To synthesise qualitative evidence related to barriers and facilitators of flexible sigmoidoscopy screening (FSS) intention and uptake, particularly within low socio-demographic uptake groups. |
| Outcomes assessed | ☒Barriers (see – Results, *'Barriers and facilitators of screening intention'*)<br>☒Facilitators |
| Type of review | A review that seeks to include:<br>☒Qualitative studies<br>☐Quantitative studies<br>☐Mixed methods studies<br>☐Not specified |
| Systematic review | Do the authors state this is a systematic review, systematic scoping review, or other form of systematic evaluation?<br>☒Yes<br>☐No |
| Screening programmes targeted by the review | ☒Bowel<br>☐Breast<br>☐Cervical |
| Focus on specific method of screening? | Flexible sigmoidoscopy |
| Details of included studies | |
| Population(s) of the included reviews | Included characteristics:<br>☒Age: Inconsistent when reported by included studies, most often listed as aged 50+<br>☒Age within screening for NHS Scotland (50-74)?:<br>☒Gender and/or sex (described by the authors): Women and men<br>☒Ethnicity: Three studies were noted as focusing on Asian ethnicities<br>☒Other: Education and/or household income noted as either a direct or indirect reporting measure in 7 studies; further varying subpopulations for each of the 10 studies are reported in the supplementary material |
| Number of studies included | 10 |
| Total number of participants across all studies | 1,343 |
| Type of studies included | ☒Qualitative studies - 10<br>☐Quantitative studies<br>☐Mixed methods studies<br>☐Not specified |
| Geographic scope (high-income countries only) | CAN; GBR; USA |
| Search strategy and methods | |
| Sources | EMBASE, MEDLINE, PsycINFO and Web of Science |
| Search restrictions (language, years, region etc) | See Table S1 for further search specifics |

Data extraction form (BOWEL)

| | |
|---|---|
| Search dates | Publications from inception to end date of March 2019; later revised to include end dates up to Jan 2020; exact dates that searches were conducted have not been provided. |
| Other criteria | Hand-searching was also conducted; use of Google Scholar's 'cited by' function to search for additional studies of relevance. |
| Instrument/tool used for quality appraisal of studies | ☒Yes: Critical Appraisal Skills Program tool<br>☐Not specified |
| Characteristics of included studies table? | ☒Yes: Table S2<br>☐No |
| Method of analysis/synthesis of results | ☐Narrative synthesis<br>☒Thematic analysis<br>☐Other qualitative analysis<br>☐Meta-analysis<br>☐Other quantitative analysis |
| Presentation of results supported by | ☐Tabulation<br>☐Framework/model<br>☐Forest plot<br>☒Other: Tree diagram showing relationship between descriptive themes |
| Key findings | |
| Key barriers and/or facilitators identified | Barriers and facilitators as identified by thematic analysis:<br>Procedural anxieties ('invasiveness', embarrassment)<br>FSS a threat to masculinity<br>Low risk perception<br>Sense of responsibility to use public funds, maintenance of health<br>Fear of the unknown<br>Understanding and value of early detection<br>Test preparation difficulties<br>Avoidant decision making about the test (lack of prevention attitude)<br>Influence of family and friends (barrier and facilitator)<br>Physician recommendation of direct personal benefit<br>Religious and cultural-influenced health beliefs<br>Preference for female physicians<br>Fatalism<br>Peace of mind<br>Competing priorities (childcare, work commitments, rescheduling/scheduling issues)<br><br>See: Results |
| Limitations of the review | – A key review limitation was the inclusion of only published peer-reviewed journals, excluding all grey literature.<br>– All studies highlighted limitations of small sample sizes and or purposeful quota sampling, stating that conclusions drawn from qualitative data alone should remain tentative. Qualitative research principles argue that findings are not intended to be generalisable, but specific to a certain context, time and set of participants. |
| Implications for future research | – Gap between intention and uptake should be further addressed in future qualitative research |

| | |
|---|---|
| | – Improve and enhance comfort and modesty to address 'procedural anxieties' (screening modifications)<br>– Further investigation and trials needed on the 'nudge technique' to explore the 'decoy effect' in gender preferences for practitioner<br>– Further appraisal needed of current UK interventions (avoidance due to low health literacy, 'think-aloud' studies may offer more immediate understanding of low uptake groups, community-based participatory research may be relevant to certain ethnic groups) |
| Other notes | N/A |
| Other | |
| Quality appraisal (JBI checklist) | ☒Include<br>☐Exclude |

| Review details | |
|---|---|
| Study ID (First author, year) | Wang (2019) |
| Title of the review | Barriers of colorectal cancer screening in rural USA: a systematic review |
| Review objectives/focus of the review | Colorectal cancer (CRC) screening rates are lower in rural areas in the USA. To guide the design of interventions to improve CRC screening, a systematic review was conducted to identify CRC screening barriers for rural populations. |
| Outcomes assessed | ☒Barriers<br>☐Facilitators |
| Type of review | A review that seeks to include:<br>☒Qualitative studies<br>☒Quantitative studies<br>☐Mixed methods studies<br>☐Not specified |
| Systematic review | Do the authors state this is a systematic review, systematic scoping review, or other form of systematic evaluation?<br>☒Yes<br>☐No |
| Screening programmes targeted by the review | ☒Bowel<br>☐Breast<br>☐Cervical |
| Focus on specific method of screening? | N/A |
| Details of included studies | |
| Population(s) of the included reviews | Included characteristics:<br>☒Age: Inconsistent when reported by included studies, most often listed as aged 50+<br>☒Age within screening for NHS Scotland (50-74)?:<br>☒Gender and/or sex (described by the authors): Women and men, or otherwise not specified<br>☒Ethnicity: 2 studies focused on Hispanic/Latino populations; 1 study reflective of respondents within African American churches, 2 studies incorporating American Indian/American Native |

| | |
|---|---|
| | perspectives<br>☒Other: Also includes studies from the perspective of primary healthcare providers; focus of the review is rurality, reflected explicitly in almost all studies |
| Number of studies included | 27 |
| Total number of participants across all studies | Unable to accurately calculate from the summary of studies information provided by the authors; unclear notion specific to numbers reported for Wilkins |
| Type of studies included | ☒Qualitative studies – *further specifics on individual study designs not provided by the authors*<br>☒Quantitative studies<br>☐Mixed methods studies<br>☐Not specified |
| Geographic scope (high-income countries only) | USA (rural) |
| Search strategy and methods | |
| Sources | Medline, CINAHL, Embase, and Scopus |
| Search restrictions (language, years, region etc) | Literature published from 1998–2017; combination keyword search strategy. |
| Search dates | The search was conducted in Dec 2017. |
| Other criteria | A manual search was conducted through journals, internet resources, and bibliographies of retrieved articles. |
| Instrument/tool used for quality appraisal of studies | ☐Yes:<br>☒Not specified |
| Characteristics of included studies table? | ☒Yes: See Table 1<br>☐No |
| Method of analysis/synthesis of results | ☒Narrative synthesis<br>☐Thematic analysis<br>☐Other qualitative analysis<br>☐Meta-analysis<br>☐Other quantitative analysis |
| Presentation of results supported by | ☒Tabulation<br>☐Framework/model<br>☐Forest plot<br>☐Other: |
| Key findings | |
| Key barriers and/or facilitators identified | Results summary:<br>The most frequently reported barriers were high screening cost and lack of insurance coverage, embarrassment or discomfort undergoing screening, lack of knowledge or perceived need on CRC screening, and lack of physician recommendation. Lack of prevention attitude toward cancer, perceived lack of privacy, shortage of specialists, and distance to test facilities were reported as rural-specific barriers for CRC screening.<br><br>See: Abstract, results summary |
| Limitations of the review | – Studies used various definitions of rural populations; refined and accurate definitions would help to better understand the rural-specific CRC screening barriers. |

| | |
|---|---|
| | – Differing quality and outcome measures for qualitative and quantitative studies included in the review.<br>– Review was limited to published, peer-reviewed, English-language articles in the specified databases and results from studies not indexed in these databases may have been under-represented |
| Implications for future research | – Additional research needed on rural-urban differences in CRC screening barriers<br>– Further studies should aim to distinguish barriers specific to FOBT as compared to endoscopies<br>– "…findings suggest strategies providing public health education, reducing costs, and increasing access to specialists may be most effective in rural areas to promote CRC screening. An evaluation of these strategies and their impact on CRC screening can help inform policy interventions in rural areas". |
| Other notes | N/A |
| Other | |
| Quality appraisal (JBI checklist) | ☒Include<br>☐Exclude |

| Review details | |
|---|---|
| Study ID (First author, year) | Wortley (2014) |
| Title of the review | Assessing Stated Preferences for Colorectal Cancer Screening: A Critical Systematic Review of Discrete Choice Experiments |
| Review objectives/focus of the review | It is well established that screening is effective in reducing the incidence and mortality associated with colorectal cancer (CRC). National screening programs have been implemented in many countries; however, uptake remains an issue. Understanding patient preferences may assist in shaping screening programs and tailoring information about screening tests. The objective was to undertake a systematic review of discrete choice experiments (DCEs) of CRC screening. |
| Outcomes assessed | ☒Barriers<br>☒Facilitators |
| Type of review | A review that seeks to include:<br>☐Qualitative studies<br>☒Quantitative studies<br>☐Mixed methods studies<br>☐Not specified |
| Systematic review | Do the authors state this is a systematic review, systematic scoping review, or other form of systematic evaluation?<br>☒Yes<br>☐No |
| Screening programmes targeted by the review | ☒Bowel<br>☐Breast<br>☐Cervical |
| Focus on specific method of screening? | N/A |

Data extraction form (BOWEL)

| Details of included studies | |
|---|---|
| Population(s) of the included reviews | Included characteristics:<br>☒Age: Inconsistent when reported by included studies, most often listed as aged 50+<br>☒Age within screening for NHS Scotland (50-74)?:<br>☒Gender and/or sex (described by the authors): Men and women<br>☐Ethnicity: Not reported<br>☒Other: One study included physicians |
| Number of studies included | 9 |
| Total number of participants across all studies | 11,055 |
| Type of studies included | ☐Qualitative studies<br>☒Quantitative studies - 9<br>☐Mixed methods studies<br>☐Not specified |
| Geographic scope (high-income countries only) | AUS; CAN; DNK; FRN; NLD; USA |
| Search strategy and methods | |
| Sources | MEDLINE, Embase, EconLit, and PreMedline databases |
| Search restrictions (language, years, region etc) | Databases searched from inception to April 2013 using a strategy aimed at identifying studies employing discrete choice methodology in screening populations; search strategy was a combination of MeSH terms and keyword searching. |
| Search dates | Exact dates that searches were conducted have not been provided. |
| Other criteria | Google Scholar was also searched using text words from the searches. |
| Instrument/tool used for quality appraisal of studies | ☒Yes: ISPOR checklist<br>☐Not specified |
| Characteristics of included studies table? | ☒Yes: See Table 1<br>☐No |
| Method of analysis/synthesis of results | ☒Narrative synthesis<br>☐Thematic analysis<br>☐Other qualitative analysis<br>☐Meta-analysis<br>☐Other quantitative analysis |
| Presentation of results supported by | ☒Tabulation: See Table 2, Key findings<br>☐Framework/model<br>☐Forest plot<br>☐Other: |
| Key findings | |
| Key barriers and/or facilitators identified | The majority of the studies reported that accuracy or clinical effectiveness (‘how many more cancers found’) was an important attribute, and that respondents were willing to trade other attributes for improvements in accuracy |

| | |
|---|---|
| | No single test seemed to be optimal, rather, respondents indicated a preference for a test that was accurate, short in duration, needed no preparation, and had no associated complications. A significant proportion preferred no screening to the currently available screening tests for CRC<br><br>See: Conclusions and 'Key Points for Decision Makers' |
| Limitations of the review | – The manner in which studies were presented and/or results and conclusions were reported varied in quality. Most studies did include some discussion around the implications of the study findings and positioned the results within the broader evidence on CRC screening.<br>– More recently published studies provided greater detail, both aiding and abetting the accuracy of the contextual overview. |
| Implications for future research | – Choice of screening tests does not always improve participation; using value-clarification tools (such as DCEs) can help "participants elucidate the characteristics of screening tests that are most important to them, thus aiding in the development of tailored communication strategies to assist in informed decision making regarding CRC screening options."<br>– As a "significant proportion preferred no screening to the currently available screening tests for CRC" preference studies will continue to be relevant in shaping new screening programs and assisting with informed consent |
| Other notes | N/A |
| Other | |
| Quality appraisal (JBI checklist) | ☒Include<br>☐Exclude |

Data extraction form (BREAST)

| Review details | |
|---|---|
| Study ID (First author, year) | Andreeva (2013) |
| Title of the review | Breast cancer screening utilization among Eastern European immigrant women worldwide: a systematic literature review and a focus on psychosocial barriers |
| Review objectives/focus of the review | Many countries host growing Eastern European immigrant communities whose breast cancer preventive behaviors are largely unknown. Thus, we aimed to synthesize current evidence regarding secondary prevention via breast cancer screening utilized by that population. |
| Outcomes assessed | ☒Barriers<br>☒Facilitators |
| Type of review | A review that seeks to include:<br>☒Qualitative studies<br>☒Quantitative studies<br>☐Mixed methods studies<br>☐Not specified |
| Systematic review | Do the authors state this is a systematic review, systematic scoping review, or other form of systematic evaluation?<br>☒Yes<br>☐No |
| Screening programmes targeted by the review | ☐Bowel<br>☒Breast<br>☐Cervical |
| Focus on specific method of screening? | Mammography |
| Details of included studies | |
| Population(s) of the included reviews | Included characteristics:<br>☒Age: Inconsistently reported across included studies; range from 18 years to 80+<br>☒Age within screening for NHS Scotland (50-70)?:<br>☒Gender and/or sex (described by the authors): Women<br>☒Ethnicity: Eastern European<br>☒Other: Immigrants; two studies specifically looked at education level among Former Soviet Union immigrant women with post-secondary education (87% and 67% of the study populations, respectively) each showing low awareness of breast cancer risk and prevention. |
| Number of studies included | 30 |
| Total number of participants across all studies | 35,755; participant numbers not reported for 4 studies |
| Type of studies included | ☒Qualitative studies – design specifics not provided by the authors for individual studies<br>☒Quantitative studies<br>☐Mixed methods studies<br>☐Not specified |
| Geographic scope (high-income countries only) | AUS; CAN; CHE; DEU; DNK; ESP; GBR; ISR; NLD; USA |
| Search strategy and methods | |
| Sources | EMBASE and MEDLINE |

Data extraction form (BREAST)

| | |
|---|---|
| Search restrictions (language, years, region etc) | See Figure 3 for detailed search strategy. |
| Search dates | Databases were searched from inception through March 2013 using indexing terms. |
| Other criteria | A manual search of the bibliographies of all relevant articles was also conducted. |
| Instrument/tool used for quality appraisal of studies | ☒Yes: Authors note "we also assessed the quality of the reviewed studies using a checklist adapted from existing quality assessment guidelines" re: Greenhalgh et al, Khan et al, and Prictor et al.<br>☐Not specified |
| Characteristics of included studies table? | ☒Yes: See Table 1<br>☐No |
| Method of analysis/synthesis of results | ☒Narrative synthesis<br>☐Thematic analysis<br>☐Other qualitative analysis<br>☐Meta-analysis<br>☐Other quantitative analysis |
| Presentation of results supported by | ☐Tabulation<br>☐Framework/model<br>☐Forest plot<br>☒Other: Primarily narrative synthesis, as supported by Table 1 |
| Key findings | |
| Key barriers and/or facilitators identified | Predictors of breast cancer screening:<br>Acculturation (positive association)<br>Low risk perception<br>Lack of understanding about value of screening, insufficient knowledge about early detection<br>No history of screening for preventative purposes<br><br>Psychosocial barriers to screening behaviour:<br>External locus of control regarding decision making in health matters (fatalism)<br>Lack of healthcare provider recommendation to attend screening; onus on healthcare providers<br><br>See: Results |
| Limitations of the review | – Data deficiencies within reviewed studies may have affected the overall quality of the review; much of the data was derived from self-reports.<br>– Lack of clear definitions around EE ethnicities (heterogeneity in sample compositions) |
| Implications for future research | – "…well-targeted and culturally tailored efforts to increase screening mammography among recent EE immigrants are needed."<br>– Critical to "expand the cancer prevention research and intervention agendas and to document the prevalence and determinants of BC-related health behaviors in that [Eastern European immigrant] vulnerable population, which could lead to increased efficacy of future initiatives." |

| | |
|---|---|
| | – Patients can become involved in their own healthcare and modify behaviours through the use of technology, including Internet sites and mobile applications |
| Other notes | N/A |
| Other | |
| Quality appraisal (JBI checklist) | ☒Include<br>☐Exclude |

| | |
|---|---|
| Review details | |
| Study ID (First author, year) | Baird (2021) |
| Title of the review | What can be done to encourage women from Black, Asian and minority ethnic backgrounds to attend breast screening? A qualitative synthesis of barriers and facilitators |
| Review objectives/focus of the review | The objective of the study is to identify the barriers to UK Black, Asian and Minority Ethnic (BAME) women attending breast screening and subsequently, support the growing evidence base providing solutions to the public health problem of ethnic variation within screening attendance. |
| Outcomes assessed | ☒Barriers<br>☒Facilitators |
| Type of review | A review that seeks to include:<br>☒Qualitative studies<br>☐Quantitative studies<br>☐Mixed methods studies<br>☐Not specified |
| Systematic review | Do the authors state this is a systematic review, systematic scoping review, or other form of systematic evaluation?<br>☒Yes<br>☐No |
| Screening programmes targeted by the review | ☐Bowel<br>☒Breast<br>☐Cervical |
| Focus on specific method of screening? | Mammography |
| Details of included studies | |
| Population(s) of the included reviews | Included characteristics:<br>☒Age: Inconsistently reported by included studies; lower age limit of 20, with upper limit of some studies 70 years of age<br>☒Age within screening for NHS Scotland (50-70)?:<br>☒Gender and/or sex (described by the authors): Women and men<br>☒Ethnicity: Black, Asian, and Minority Ethnic (BAME)<br>☒Other: One study noted women from an array of social and economic backgrounds were selected to represent Hackney's diversity; another study noted women living in Luton must have had a conversational level of English to participate. One study describes the population as 67 breast screening clinics across England (survey design, 70% response rate). |
| Number of studies included | 8 |

Data extraction form (BREAST)

| | |
|---|---|
| Total number of participants across all studies | 2,335 (2,285 women and 50 men); two studies did not report participant numbers, one study noted the population as number of breast screening clinics |
| Type of studies included | ☒Qualitative studies - 8<br>☐Quantitative studies<br>☐Mixed methods studies<br>☐Not specified |
| Geographic scope (high-income countries only) | GBR |
| Search strategy and methods | |
| Sources | MEDLINE, EMBASE and PubMed |
| Search restrictions (language, years, region etc) | Databases searched up to Dec 2017 using predefined inclusion/exclusion criteria. |
| Search dates | Dates of the search process were not stated. |
| Other criteria | N/A |
| Instrument/tool used for quality appraisal of studies | ☒Yes: CASP toolkit.<br>☐Not specified |
| Characteristics of included studies table? | ☒Yes:<br>☐No |
| Method of analysis/synthesis of results | ☐Narrative synthesis<br>☒Thematic analysis<br>☐Other qualitative analysis<br>☐Meta-analysis<br>☐Other quantitative analysis |
| Presentation of results supported by | ☒Tabulation<br>☐Framework/model<br>☐Forest plot<br>☒Other: Illustrative quotes |
| Key findings | |
| Key barriers and/or facilitators identified | Knowledge-related factors:<br>All eight included studies reported knowledge as either a barrier or facilitator to breast screening attendance. Overall, the main barriers presented for BAME women were a lack of knowledge surrounding the following: what is breast cancer, how to identify it, what is the screening programme, who is at risk and the treatments available.<br><br>Access-related factors:<br>Logistical (distance, inconvenience, and cost) and emotional barriers were the main causes of decreased access of breast screening services.<br><br>Cultural-related factors:<br>This broad category encompassed factors such as cultural values, religious beliefs and the influence of family and friends. Influence of faith could lead to fatalism; stigma surrounding marital consequences of a diagnosis; gender of healthcare professional also a barrier. Reluctance of healthcare providers to engage with BAME women due to feelings of cultural incompetence, and specific needs of BAME women and how to address them. Past |

| | |
|---|---|
| | negative experiences with healthcare professionals were a barrier to attendance, while strong interpersonal relationships encouraged screening attendance.<br><br>See: Results |
| Limitations of the review | – To maximise the reliability and validity of results, the search strategy specified 'articles from peer-reviewed journals.' Grey literature, such as PhD theses and unpublished research, was not included in the search and therefore, there is potential for selection bias to influence results as a consequence of this exclusion. Furthermore, generalisability is potentially impacted due to the exclusion limiting the evidence scope.<br>– The primary articles included in this review were subjective to varying degrees of methodological shortcomings yielding less robust conclusions therefore, to limit the extent of this, evidence from weaker studies was only included if stronger studies supported it.<br>– Adapted CASP tool may have been less accurate which impacts the strength of the conclusions. |
| Implications for future research | – The influence of barriers/facilitators for screening programme uptake may vary within different ethnic communities<br>– "The patients' decision to attend screening is directly influenced by their encounters with healthcare staff. There is, therefore, a real need to better educate healthcare staff on the public health implications following their patient interaction." |
| Other notes | N/A |
| Other | |
| Quality appraisal (JBI checklist) | ☒Include<br>☐Exclude |

| Review details | |
|---|---|
| Study ID (First author, year) | Jerome-D'Emilia (2015) |
| Title of the review | A Systematic Review of Barriers and Facilitators to Mammography in Hispanic Women |
| Review objectives/focus of the review | A systematic review of the research on barriers and facilitators to mammography in Latinas was conducted to determine if the challenges faced by these women are unique to this population. |
| Outcomes assessed | ☒Barriers<br>☒Facilitators |
| Type of review | A review that seeks to include:<br>☐Qualitative studies<br>☒Quantitative studies – limited to quantitative studies as noted in the search strategy; authors report all studies were cross-sectional surveys<br>☐Mixed methods studies<br>☐Not specified |
| Systematic review | Do the authors state this is a systematic review, systematic scoping review, or other form of systematic evaluation? |

Data extraction form (BREAST)

| | |
|---|---|
| | ☒Yes<br>☐No |
| Screening programmes targeted by the review | ☐Bowel<br>☒Breast<br>☐Cervical |
| Focus on specific method of screening? | Mammography |
| **Details of included studies** | |
| Population(s) of the included reviews | Included characteristics:<br>☒Age: Inconsistently reported across all studies; lower range of 18 and upper range to 79 when reported<br>☒Age within screening for NHS Scotland (50-70)?:<br>☒Gender and/or sex (described by the authors): Women<br>☒Ethnicity: Latina/Hispanic<br>☒Other: Low-income (17 studies considered income, with the majority of respondents earning $10,000 USD or less per year – the authors hypothesize the remaining one study was also representative of low-income, as the sample consisted of relatively recent immigrants). |
| Number of studies included | 18 |
| Total number of participants across all studies | 6,866 |
| Type of studies included | ☐Qualitative studies<br>☒Quantitative studies<br>☐Mixed methods studies<br>☐Not specified |
| Geographic scope (high-income countries only) | USA |
| **Search strategy and methods** | |
| Sources | MEDLINE and CINAHL databases |
| Search restrictions (language, years, region etc) | Keyword searching was conducted within databases; see Figure 1 for the full inclusion/exclusion process. |
| Search dates | Exact dates of the search process were not reported. |
| Other criteria | N/A |
| Instrument/tool used for quality appraisal of studies | ☐Yes:<br>☒Not specified |
| Characteristics of included studies table? | ☒Yes: see Table 1<br>☐No |
| Method of analysis/synthesis of results | ☒Narrative synthesis<br>☐Thematic analysis<br>☐Other qualitative analysis<br>☐Meta-analysis<br>☐Other quantitative analysis |
| Presentation of results supported by | ☒Tabulation<br>☐Framework/model<br>☐Forest plot<br>☒Other: Narrative synthesis supported by Table 1 |
| **Key findings** | |

Data extraction form (BREAST)

| | |
|---|---|
| Key barriers and/or facilitators identified | Barriers and facilitators included: familism, acculturation, physician recommendation as a cute to action, health literacy (language); this review found no significant difference among level of knowledge and medical history (awareness of family history).<br><br>See: Results |
| Limitations of the review | – Each of these studies was based on a cross-sectional design. As such, neither can causality be determined nor can opposing hypotheses be disconfirmed. Many of the studies relied on convenience sampling that impedes the study's generalizability. Even in cases of random sampling, sample sizes were small.<br>– Each of these studies relied on a woman's self-report of mammogram use. Although there has been only one study that addressed the validity of self-reported mammograms in Latinas, this study found that Latinas have a lower rate of agreement between self-report and medical record as compared with African American and non-Hispanic White women. |
| Implications for future research | – A physician's recommendation (cue to action) "has been found to be the strongest predictor of mammogram use across all ages and populations of women."<br>– "In order to encourage and support women in their health-seeking efforts, nurses must be cognizant of the factors that enable women to seek preventive care"; this includes education, poor breast cancer knowledge, insurance coverage and costs, and lack of a physician's recommendation |
| Other notes | N/A |
| Other | |
| Quality appraisal (JBI checklist) | ☒Include<br>☐Exclude |

| Review details | |
|---|---|
| Study ID (First author, year) | Jerome-D'Emilia (2019) |
| Title of the review | A Systematic Review of Barriers and Facilitators to Mammography in American Indian/Alaska Native Women |
| Review objectives/focus of the review | The purpose of this systematic review was to synthesize the current knowledge of factors that enable or impede American Indian and Alaska Native (AI/AN) women from accessing breast cancer screening. |
| Outcomes assessed | ☒Barriers<br>☒Facilitators |
| Type of review | A review that seeks to include:<br>☒Qualitative studies<br>☒Quantitative studies<br>☐Mixed methods studies<br>☐Not specified |
| Systematic review | Do the authors state this is a systematic review, systematic scoping review, or other form of systematic evaluation? |

Data extraction form (BREAST)

| | |
|---|---|
| | ☒Yes<br>☐No |
| Screening programmes targeted by the review | ☐Bowel<br>☒Breast<br>☐Cervical |
| Focus on specific method of screening? | Mammography |
| Details of included studies | |
| Population(s) of the included reviews | Included characteristics:<br>☒Age: Inconsistent when reported by studies; lower age range of 18 for some studies, highest age range noted as 82<br>☒Age within screening for NHS Scotland (50-70)?:<br>☒Gender and/or sex (described by the authors): Women<br>☒Ethnicity: American Indian (AI) and Alaska Native (AN)<br>☒Other: Authors note studies overall incorporate socioeconomic factors such as lack of insurance, lower income and less education, inadequate access to care, and the absence of a usual source of care. One study includes 12 tribal staff; one additional study includes 17 community health providers and 13 community leaders. |
| Number of studies included | 18 |
| Total number of participants across all studies | 26,049 (authors note for some studies it is unclear how many participants were AI/AN) |
| Type of studies included | ☒Qualitative studies - 7<br>☒Quantitative studies – 8<br>☐Mixed methods studies<br>☒Not specified – 3 intervention studies |
| Geographic scope (high-income countries only) | USA (incl some rural) |
| Search strategy and methods | |
| Sources | MEDLINE and CINAHL databases |
| Search restrictions (language, years, region etc) | The search limits were original research studies written in the English language. Inclusion criteria included NA/AI women; age 40 years and older (at least as a subsample); with attitudes, barriers, and facilitators to mammography; and studies based in the United States. |
| Search dates | Studies published in a 10-year period, from 2007 to 2017. |
| Other criteria | N/A |
| Instrument/tool used for quality appraisal of studies | ☐Yes:<br>☒Not specified |
| Characteristics of included studies table? | ☒Yes: see Table 1<br>☐No |
| Method of analysis/synthesis of results | ☒Narrative synthesis<br>☐Thematic analysis<br>☐Other qualitative analysis<br>☐Meta-analysis<br>☐Other quantitative analysis |

Data extraction form (BREAST)

| | |
|---|---|
| Presentation of results supported by | ☒Tabulation<br>☐Framework/model<br>☐Forest plot<br>☒Other: Narrative synthesis supported by Table 1 |
| **Key findings** | |
| Key barriers and/or facilitators identified | Breast cancer screening barriers within scope of overview:<br>Barriers specifically noted for AI/AN women include geographic isolation, language barriers, lack of transportation and child care, and traditional and cultural issues. Preference for traditional healers/"traditionality" were also significant barriers to screening. Fatalism is also associated with a lower likelihood of screening.<br><br>Breast cancer screening facilitators within scope of overview:<br>Factors that would facilitate screening for AI/AN women included a physician's recommendation, specifically if the physician made a referral for a certain facility, or if the woman could be screened the same day she received the recommendation.<br><br>See: Results |
| Limitations of the review | – Potential for missed literature as part of the search process; local screening practices that may not be documented within the literature.<br>– Limited studies conducted in this population may limit the generalizability of findings.<br>– An additional factor, which was demonstrated in the studies examined in this review, is that while there is a great deal of diversity in the AI/AN population, many studies aggregate or "lump" members of various tribes located in very different parts of the United States with diverse cultural traditions and histories into one circumscribed group. |
| Implications for future research | – "The qualitative studies suggest that women may be more responsive to locally supportive, targeted, and culturally appropriate interventions that respect traditionality, yet encourage trust in the medical system."<br>– Specific to the United States, working with "tribes in the development of interventions framed by community-based participatory research are needed to tackle the disparities in the AI/AN community." |
| Other notes | – Focus also on sociodemographic elements, which have been omitted for the purpose of the overview as these are not within current project scope. |
| **Other** | |
| Quality appraisal (JBI checklist) | ☒Include<br>☐Exclude |

| | |
|---|---|
| **Review details** | |
| Study ID (First author, year) | Oh (2017) |
| Title of the review | Breast Cancer Screening Among Korean Americans: A Systematic Review |

Data extraction form (BREAST)

| | |
|---|---|
| Review objectives/focus of the review | Cancer is the leading cause of death for Korean Americans (KAs). Breast cancer (BC) is the most commonly occurring cancer among KA women, and its rate has been rapidly increasing. Low BC screening rates for KAs puts them at greater risk for late-stage breast cancer. No previous systematic review has evaluated the full body of literature on breast cancer screening facilitators and barriers among KAs. This paper examines the prevalence of breast cancer screening practices among KAs and identifies the factors that might positively influence breast cancer screening rates in future health promotion interventions targeted at the KA population. |
| Outcomes assessed | ☒Barriers<br>☒Facilitators |
| Type of review | A review that seeks to include:<br>☒Qualitative studies<br>☒Quantitative studies<br>☐Mixed methods studies<br>☐Not specified |
| Systematic review | Do the authors state this is a systematic review, systematic scoping review, or other form of systematic evaluation?<br>☒Yes<br>☐No |
| Screening programmes targeted by the review | ☐Bowel<br>☒Breast<br>☐Cervical |
| Focus on specific method of screening? | Mammography |
| Details of included studies | |
| Population(s) of the included reviews | Included characteristics:<br>☒Age: ≥ 40 years of age<br>☒Age within screening for NHS Scotland (50-70)?:<br>☒Gender and/or sex (described by the authors): Women<br>☒Ethnicity: Korean Americans<br>☐Other: |
| Number of studies included | 38 |
| Total number of participants across all studies | 13,341; two studies did not report participant numbers |
| Type of studies included | ☒Qualitative studies<br>☒Quantitative studies<br>☐Mixed methods studies<br>☒Not specified: Identified by the others as 9 papers reporting on interventions, and the remainder (29) as observational studies using a mix of interview and self-administered questionnaires. |
| Geographic scope (high-income countries only) | USA |
| Search strategy and methods | |
| Sources | Medline, the Cumulative Index to Nursing and Allied Health Literature (CINAHL), the Educational Resources Information Center (ERIC), the American Psychological Association’s PsycINFO, |

| | |
|---|---|
| | and four EBSCO databases (Academic Search Complete, Communication and Mass Media Complete, Health Source: Nursing/Academic Edition, and the Psychology and Behavioral Sciences Collection) |
| Search restrictions (language, years, region etc) | No publication date or language restrictions applied. |
| Search dates | Search conducted in July 2016; no further details on years searched provided. |
| Other criteria | N/A |
| Instrument/tool used for quality appraisal of studies | ☐Yes:<br>☒Not specified |
| Characteristics of included studies table? | ☒Yes: See Table 1<br>☐No |
| Method of analysis/synthesis of results | ☒Narrative synthesis<br>☐Thematic analysis<br>☐Other qualitative analysis<br>☐Meta-analysis<br>☐Other quantitative analysis |
| Presentation of results supported by | ☒Tabulation<br>☐Framework/model<br>☐Forest plot<br>☐Other: |
| Key findings | |
| Key barriers and/or facilitators identified | Screening rates are higher among adults with higher socioeconomic status, greater acculturation to the United States, more cancer knowledge, higher perceived susceptibility to BC, more social support, and better access to health services.<br><br>However, fear of finding something wrong, fear of embarrassment or lack of modesty, not knowing where to go for screening, believing that mammography is only necessary when symptoms are present, and perceived time and cost difficulties in accessing mammography were reported as barriers to mammogram screening.<br><br>See: Results, abstract |
| Limitations of the review | Not reported. |
| Implications for future research | – “Additional research on effective interventions for improving screening among KAs is needed, but a trusted clinician will certainly have a positive impact on encouraging patients to seek screening.”<br>– “Other tools for increasing breast cancer screening among KA women may include culturally-tailored cancer education materials accessible to adults with limited English literacy that provide information about breast cancer and screening guidelines, the importance of regular screening in the absence of symptoms, and the procedures for scheduling and receiving breast screening. Social networks such as churches, cultural organizations, and KA doctors’ offices can provide |

| | |
|---|---|
| | assistance with patient navigation, including supporting transportation and translation services” |
| Other notes | N/A |
| Other | |
| Quality appraisal (JBI checklist) | ☒Include<br>☐Exclude |

| | |
|---|---|
| Review details | |
| Study ID (First author, year) | Pagliarin (2021) |
| Title of the review | Are women satisfied with their experience with breast cancer screening? Systematic review of the literature |
| Review objectives/focus of the review | A systematic review to assess how satisfied women were with organized breast cancer screening programs. |
| Outcomes assessed | ☒Barriers *(focus on ‘experiences’ of breast cancer screening programmes)*<br>☒Facilitators |
| Type of review | A review that seeks to include:<br>☐Qualitative studies<br>☒Quantitative studies<br>☐Mixed methods studies<br>☐Not specified |
| Systematic review | Do the authors state this is a systematic review, systematic scoping review, or other form of systematic evaluation?<br>☒Yes<br>☐No |
| Screening programmes targeted by the review | ☐Bowel<br>☒Breast<br>☐Cervical |
| Focus on specific method of screening? | Mammography |
| Details of included studies | |
| Population(s) of the included reviews | Included characteristics:<br>☐Age: Not reported<br>☐Age within screening for NHS Scotland (50-70)?: N/A<br>☒Gender and/or sex (described by the authors): Women<br>☐Ethnicity: Not reported<br>☐Other: |
| Number of studies included | 48 |
| Total number of participants across all studies | 44,659 |
| Type of studies included | ☐Qualitative studies<br>☒Quantitative studies – 48 questionnaires reporting quantitative measures<br>☐Mixed methods studies<br>☐Not specified |
| Geographic scope (high-income countries only) | AUS; DNK; ESP; GBR; HRV; IRL; ISR; KOR; NLD; NOR; USA |
| Search strategy and methods | |

Data extraction form (BREAST)

| | |
|---|---|
| Sources | Medline, CINAHL, Embase and PsycINFO |
| Search restrictions (language, years, region etc) | See the Supplementary material for full details. |
| Search dates | 1965 to October 2019 |
| Other criteria | N/A |
| Instrument/tool used for quality appraisal of studies | ☒Yes: EQUATOR checklist<br>☐Not specified |
| Characteristics of included studies table? | ☒Yes: See the Supplementary material for full details<br>☐No |
| Method of analysis/synthesis of results | ☒Narrative synthesis<br>☐Thematic analysis<br>☐Other qualitative analysis<br>☐Meta-analysis<br>☐Other quantitative analysis |
| Presentation of results supported by | ☒Tabulation<br>☐Framework/model<br>☐Forest plot<br>☐Other: |
| Key findings | |
| Key barriers and/or facilitators identified | Experiences with screening mammography resulting in uptake or willingness to re-screen:<br>General satisfaction (facilitator); negative assessment of the staffs attitude; discomfort or pain during mammography<br><br>Staff's interpersonal skills (facilitator); information transfer and clear communication<br><br>Physical surroundings and privacy (overall satisfaction with services)<br><br>Accessibility and wait times; public transport, traffic, parking, work and domestic arrangements in order to attend the appointment. Difficult reaching mammography centre shown with lower willingness to re-attend. Quick delivery of results preferred.<br><br>Sufficient training in performing the test (facilitator).<br><br>See: Results |
| Limitations of the review | – Lack of validation tools for included studies; heterogeneity of the outcomes measures in different studies, which limits the comparability and interpretation of results.<br>– In order to enable comparison of women's experiences with various screening programs and in different countries, a clear definition of 'screening satisfaction' would be required, and harmonized measures should be developed and implemented. |
| Implications for future research | – "Due to the key role played by the staff's communication skills, future efforts to improve screening satisfaction should include the improvement of staff´s communication skills training." |

| | |
|---|---|
| | – Minimizing the wait time for screening results is optimal to alleviate potential concerns from participants with clear communication of expected timeframes<br>– Screening facilities should prioritize privacy and create a welcoming environment within their physical space<br>– "Implementing CBE as a regular screening method, in addition to a mammography might be helpful to improve the satisfaction and perceived quality of screening." |
| Other notes | N/A |
| Other | |
| Quality appraisal (JBI checklist) | ☒Include<br>☐Exclude |

Data extraction form (CERVICAL)

| Review details | |
|---|---|
| Study ID (First author, year) | Alam (2021) |
| Title of the review | Cervical Cancer Screening Among Immigrant Women Residing in Australia: A Systematic Review |
| Review objectives/focus of the review | This study systematically reviewed evidence on cervical cancer screening practices among immigrant women in Australia. |
| Outcomes assessed | ☒Barriers *(focus on barriers, overall 'willingness to participate' but some facilitators also noted)*<br>☒Facilitators |
| Type of review | A review that seeks to include:<br>☒Qualitative studies<br>☒Quantitative studies<br>☒Mixed methods studies<br>☐Not specified |
| Systematic review | Do the authors state this is a systematic review, systematic scoping review, or other form of systematic evaluation?<br>☒Yes<br>☐No |
| Screening programmes targeted by the review | ☐Bowel<br>☐Breast<br>☒Cervical |
| Focus on specific method of screening? | N/A |
| Details of included studies | |
| Population(s) of the included reviews | Included characteristics:<br>☒Age: Noted by authors as between 18-77 years<br>☒Age within screening for NHS Scotland (25-64)?<br>☒Gender and/or sex (described by the authors): Women<br>☒Ethnicity: 8 studies noted as multi-ethnic; ethnicities include former Yugoslavia, Vietnamese, Chinese, Italian, Assyrian, Russian, African/West African, Thai, Arabic<br>☒Other: Immigrants; socioeconomic status reported in some studies, noted by authors as most likely to affect 'opportunity' as suggested by four different studies included in the overall review |
| Number of studies included | 25 |
| Total number of participants across all studies | 1,266,620; two studies did not report the number of participants |
| Type of studies included | ☒Qualitative studies - 6<br>☒Quantitative studies - 16<br>☒Mixed methods studies - 3<br>☐Not specified |
| Geographic scope (high-income countries only) | AUS |
| Search strategy and methods | |
| Sources | MEDLINE, Embase, PubMed, PsycINFO, and CINAHL |
| Search restrictions (language, years, region etc) | See:<br>Supplementary Table 1 for the full electronic search strategy |
| Search dates | From inception until March 2019 |

Data extraction form (CERVICAL)

| Other criteria | PROSPERO: CRD42019130073 |
|---|---|
| Instrument/tool used for quality appraisal of studies | ☒Yes: Mixed-methods assessment tool (MMAT)<br>☐Not specified |
| Characteristics of included studies table? | ☐Yes:<br>☒No – in-text presentation only, see heading *'Characteristics of Reviewed Studies'* supported by Tables 1 and 2 |
| Method of analysis/synthesis of results | ☐Narrative synthesis<br>☐Thematic analysis<br>☒Other qualitative analysis – COM-B model of behaviour<br>☐Meta-analysis<br>☐Other quantitative analysis |
| Presentation of results supported by | ☒Tabulation – see Tables 1 and 2, 4a-d<br>☐Framework/model<br>☐Forest plot<br>☐Other: |
| Key findings | |
| Key barriers and/or facilitators identified | Key factors affecting women's willingness to participate in screening were found within qualitative studies and included insufficient knowledge (extent of the knowledge women have about cervical cancer and Pap tests), low-risk perception, and unavailability of a female health professional<br><br>The COM-B model of behaviour was used to further identify barriers of traditional and religious beliefs, English language proficiency, and the duration of stay in Australia<br><br>See: Abstract + "What this Article Adds" |
| Limitations of the review | – Lack of reliable methods of cervical screening uptake measurement across studies; lack of theoretical models to further support studies<br>– Meta-analysis was not possible due to heterogenous outcome measures<br>– Methodological quality of the studies could not be adequate assessed as 1/3 of the studies were conducted before 2000 and the development of reporting criteria; no study was excluded on the basis of quality |
| Implications for future research | – "…importance should be given to assess the comprehensive understanding that immigrant women have about cervical cancer and its screening test, and develop interventions to improve it. One of the possible ways could be development of informative sources in the native language but steps need to ensure their availability and accessibility to the migrant women."<br>– Interventions to raise awareness among healthcare providers of a lack of cervical cancer screening among culturally and linguistically diverse communities is needed; "evidence suggests that targeted interventions for GPs involving elements of education and behavioral change can be helpful."<br>– The review found preference towards same-gendered healthcare providers and acknowledged that further steps |

| | |
|---|---|
| | should be taken to provide more access to female GPs for immigrant women, particularly those who have not yet acclimated to the Australian healthcare system (recently arrived immigrants, those living in Australian for a shorter period of time) |
| Other notes | N/A |
| Other | |
| Quality appraisal (JBI checklist) | ☒Include<br>☐Exclude |

| Review details | |
|---|---|
| Study ID (First author, year) | Biddell (2020) |
| Title of the review | Variation in Cervical Cancer Screening Preferences Among Medically Underserved Individuals in the US: A Systematic Review |
| Review objectives/focus of the review | Cervical cancer screening preferences among medically underserved patients in the United States. |
| Outcomes assessed | ☐Barriers<br>☒Facilitators *(focus on 'preferences', i.e. more likely to increase screening uptake; barriers are mentioned in the context of previous reviews)* |
| Type of review | A review that seeks to include:<br>☒Qualitative studies<br>☒Quantitative studies<br>☒Mixed methods studies<br>☐Not specified |
| Systematic review | Do the authors state this is a systematic review, systematic scoping review, or other form of systematic evaluation?<br>☒Yes<br>☐No |
| Screening programmes targeted by the review | ☐Bowel<br>☐Breast<br>☒Cervical |
| Focus on specific method of screening? | Reflects preferences on both HPV self-testing and traditional Pap testing |
| Details of included studies | |
| Population(s) of the included reviews | Included characteristics:<br>☒Age: 21-65<br>☒Age within screening for NHS Scotland (25-64)?<br>☒Gender and/or sex (described by the authors): Women; two studies describe transmasculine experiences; one study focused on transgender men<br>☒Ethnicity: Variable by study when reported (provided for 11 studies); see Table 2<br>☒Other: Medically underserved; categorized broadly as racial and ethnic minorities in 30 studies, low-income populations in 7 studies, LGBTQ populations in 4 studies, rural residents in 3 studies, homeless women in 1 study, and women residing in domestic violence shelters in 1 study (note: some populations are intersectional) |

Data extraction form (CERVICAL)

| | |
|---|---|
| Number of studies included | 43 |
| Total number of participants across all studies | 11,494 |
| Type of studies included | ☒Qualitative studies - 17<br>☒Quantitative studies - 23<br>☒Mixed methods studies - 3<br>☐Not specified |
| Geographic scope (high-income countries only) | USA (including some rural) |
| **Search strategy and methods** | |
| Sources | Medline (PubMed), Science Citation Index (Web of Science), EMBASE, Scopus, CINAHL, and PsycINFO |
| Search restrictions (language, years, region etc) | See: Supplementary Material for the full search strategy |
| Search dates | From inception through February 2019 |
| Other criteria | PROSPERO: CRD42019125431 |
| Instrument/tool used for quality appraisal of studies | ☒Yes: Purnell and colleagues (2014), risk of bias template provided in Supplementary Material<br>☐Not specified |
| Characteristics of included studies table? | ☒Yes: Split across four tables, T1-4<br>☐No |
| Method of analysis/synthesis of results | ☒Narrative synthesis<br>☐Thematic analysis<br>☐Other qualitative analysis<br>☐Meta-analysis<br>☐Other quantitative analysis |
| Presentation of results supported by | ☒Tabulation – see T1-4<br>☐Framework/model<br>☐Forest plot<br>☐Other: |
| **Key findings** | |
| Key barriers and/or facilitators identified | Consistent preferences for HPV self-testing (over 90% in screening modality studies) over traditional Pap testing highlight a key potential mechanism for increasing cervical cancer screening uptake among medically underserved populations.<br><br>Additionally, preferences for gender- and language-concordant providers underscore the need for continued efforts toward expanding diversity among medical professionals.<br><br>Patient-centred communication (in-person conversations with doctors, calm demeanour of health professional who can explain the screening process)<br><br>See: Discussion |
| Limitations of the review | – Inability to fully characterise the populations studied (varying definitions of 'medically underserved')<br>– Need for standardized preference elicitation studies to better understand differing populations |

Data extraction form (CERVICAL)

| | |
|---|---|
| Implications for future research | – "Findings suggest that there is significant heterogeneity of patient preferences across populations and individuals, pointing to the importance of assessing preferences among individuals designed to benefit from a given intervention."<br>– Synthesis of included studies found a preference for HPV self-tests over Pap tests and preference for providers who "reflect patient gender, language, and life experience. These preferences must be recognized and leveraged by relevant stakeholders in the development of programs and policies to increase cervical cancer screening uptake among individuals most at risk." |
| Other notes | N/A |
| Other | |
| Quality appraisal (JBI checklist) | ☒Include<br>☐Exclude |

| | |
|---|---|
| Review details | |
| Study ID (First author, year) | Chan (2017) |
| Title of the review | A Systematic Review of the Factors Influencing Ethnic Minority Women's Cervical Cancer Screening Behavior From Intrapersonal to Policy Level |
| Review objectives/focus of the review | The aims of this study were to examine the factors that influence ethnic minority women in using cervical cancer screening and the similarities and differences in associated factors across different groups and to explore the interrelationships between the factors identified. |
| Outcomes assessed | ☒Barriers *(focus on how 'factors' influence behaviour – both barriers and facilitators presented)*<br>☒Facilitators |
| Type of review | A review that seeks to include:<br>☒Qualitative studies<br>☒Quantitative studies<br>☐Mixed methods studies<br>☐Not specified |
| Systematic review | Do the authors state this is a systematic review, systematic scoping review, or other form of systematic evaluation?<br>☒Yes<br>☐No |
| Screening programmes targeted by the review | ☐Bowel<br>☐Breast<br>☒Cervical |
| Focus on specific method of screening? | Papanicolaou tests |
| Details of included studies | |
| Population(s) of the included reviews | Included characteristics:<br>☒Age: reported ages ranged from 18-83 years<br>☒Age within screening for NHS Scotland (25-64)?<br>☒Gender and/or sex (described by the authors): Women<br>☒Ethnicity: Grouped into four categories by the authors – |

| | |
|---|---|
| | Hispanic or Latino, black, Asian, and Inuit<br>☐Other: |
| Number of studies included | 23 |
| Total number of participants across all studies | 18,170 |
| Type of studies included | ☒Qualitative studies - 2<br>☒Quantitative studies - 21<br>☐Mixed methods studies<br>☐Not specified |
| Geographic scope (high-income countries only) | AUS; CAN; GBR; USA |
| Search strategy and methods | |
| Sources | Ovid MEDLINE, Cumulative Index to Nursing and Allied Health Literature Plus, Scopus, PsycINFO, and PubMed |
| Search restrictions (language, years, region etc) | Keyword and phrased searching; secondar search conducted of studies' reference lists to identify addition sources |
| Search dates | Search conducted Oct 2015 across five databases from inception. |
| Other criteria | N/A |
| Instrument/tool used for quality appraisal of studies | ☒Yes: See Table 2 for quality assessment (tool not specified)<br>☐Not specified |
| Characteristics of included studies table? | ☒Yes: See Table 1<br>☐No |
| Method of analysis/synthesis of results | ☒Narrative synthesis<br>☐Thematic analysis<br>☐Other qualitative analysis<br>☐Meta-analysis<br>☐Other quantitative analysis |
| Presentation of results supported by | ☒Tabulation<br>☐Framework/model<br>☐Forest plot<br>☐Other: |
| Key findings | |
| Key barriers and/or facilitators identified | The findings showed that certain factors commonly affected ethnic minority women's screening behavior, including knowledge, attitude and perceptions, physician's recommendation, quality of care and service, language, and acculturation.<br><br>Culture-related factors, religion, and acculturation exhibited close interrelationships with the attitude and perceptions factor, resulting in behavioral change.<br><br>See: Results |
| Limitations of the review | – Results may not be generalizable to other geographies, as most included studies were from the United States<br>– Ethnicity was often poorly defined in the included studies, and authors chose four groupings (Hispanic or Latino, black, Asian, and Inuit) that may have differing contexts in other geographies |

| | |
|---|---|
| Implications for future research | – Multilevel models are needed to better understand screening behaviour including interpersonal, organizational, community, and the role of government and policy<br>– "Government and policy makers might revise their strategies to promote screening uptake in other ways, rather than simply supporting testing costs and insurance coverage. Interventions directed at these levels may help to improve the screening uptake by increasing the facilities available and accessible, supporting childcare, having female physicians speaking the minority's language, and overcoming culture-related influences that deter screening."<br>– "Policy makers or service providers may also consider their users' concerns, such as the issues of trust and the confidentiality of personal information that US black women emphasize. Service providers should state clearly the strategies they use to maintain the confidentiality of personal information and implement the strategies as needed to help in building a trusting and positive relationship between the healthcare providers and black women.<br>– "It may also be important to revise the public health education currently directed at black people"; this includes raising awareness of risk factors for cervical cancer as a means to change screening behaviour |
| Other notes | N/A |
| Other | |
| Quality appraisal (JBI checklist) | ☒Include<br>☐Exclude |

| Review details | |
|---|---|
| Study ID (First author, year) | Chorley (2017) |
| Title of the review | Experiences of cervical screening and barriers to participation in the context of an organised programme: a systematic review and thematic synthesis |
| Review objectives/focus of the review | This systematic review synthesises the qualitative literature on women's perceptions and experiences of cervical screening in the context of an organised call–recall programme to understand the barriers to informed uptake. |
| Outcomes assessed | ☒Barriers *(primarily barriers, but some positive facilitators also discussed)*<br>☒Facilitators |
| Type of review | A review that seeks to include:<br>☒Qualitative studies<br>☐Quantitative studies<br>☐Mixed methods studies<br>☐Not specified |
| Systematic review | Do the authors state this is a systematic review, systematic scoping review, or other form of systematic evaluation?<br>☒Yes<br>☐No |

Data extraction form (CERVICAL)

| | |
|---|---|
| Screening programmes targeted by the review | ☐Bowel<br>☐Breast<br>☒Cervical |
| Focus on specific method of screening? | N/A |
| **Details of included studies** | |
| Population(s) of the included reviews | Included characteristics:<br>☒Age: Ranges reported from 16-75; not reported for eight studies<br>☒Age within screening for NHS Scotland (25-64)?<br>☒Gender and/or sex (described by the authors): Women<br>☒Ethnicity: As described by the authors, 14 reviews focused on BAME women<br>☒Other: Varying forms of socioeconomic disadvantage described by three studies; one study focused on deaf women, one study focused on women with mild to moderate learning disabilities, and one additional study with lesbian and bisexual women |
| Number of studies included | 39 |
| Total number of participants across all studies | 1,868; two studies did not report the number of participants |
| Type of studies included | ☒Qualitative studies - 39<br>☐Quantitative studies<br>☐Mixed methods studies<br>☐Not specified |
| Geographic scope (high-income countries only) | AUS; GBR; KOR; SWE |
| **Search strategy and methods** | |
| Sources | MEDLINE, PsycINFO, Embase, Social Policy and Practice, CINAHL Plus, ProQuest Social Science Journals, Anthrosource, POPLINE and Web of Science |
| Search restrictions (language, years, region etc) | See Supporting Information 1, Supporting Information 2 |
| Search dates | Search conducted 12 Feb 2015 (no further details provided) |
| Other criteria | PROSPERO: CRD42015017075 |
| Instrument/tool used for quality appraisal of studies | ☒Yes: CASP qualitative checklist<br>☐Not specified |
| Characteristics of included studies table? | ☒Yes: See Table 1<br>☐No |
| Method of analysis/synthesis of results | ☐Narrative synthesis<br>☒Thematic analysis<br>☐Other qualitative analysis<br>☐Meta-analysis<br>☐Other quantitative analysis |
| Presentation of results supported by | ☐Tabulation<br>☒Framework/model: See Figure 1 for relationships between identified themes, presented as a diagram; further illustrative quotes are provided in Supporting Information 5 |

| | |
|---|---|
| | ☐Forest plot<br>☐Other: |
| Key findings | |
| Key barriers and/or facilitators identified | Two broad themes were identified: (a) should I go for screening? and (b) screening is a big deal.<br><br>In considering whether to attend, women discussed the personal relevance and value of screening. Women who had previously attended described how it was a big deal, physically and emotionally, and the varied threats that screening presents.<br><br>Practical barriers (language, low literacy, racist treatment) affected whether women translated screening intentions into actions.<br><br>See: Results |
| Limitations of the review | – Further research needed to also include perspectives of women who have never attended screening.<br>– Results may not be generalizable due to limited geography. |
| Implications for future research | – "The variation in women's understanding and perceptions of cervical screening suggests that interventions tailored to decisional stage may be of value in increasing engagement with the invitation and uptake of screening in those who wish to take part."<br>– "There is also a need for further research with women who have never attended screening, especially those who remain unaware or unengaged, as their perspectives are lacking in the existing literature." |
| Other notes | N/A |
| Other | |
| Quality appraisal (JBI checklist) | ☒Include<br>☐Exclude |

| Review details | |
|---|---|
| Study ID (First author, year) | Christy (2021) |
| Title of the review | Understanding Black Women's Perspectives and Experiences of Cervical Cancer Screening: A Systematic Review and Qualitative Meta-synthesis |
| Review objectives/focus of the review | Effective population-based cervical cancer screening programs exist, but improvements in morbidity and mortality continue to demonstrate racial disparities. This review uses an intersectional lens to understand Black women's experiences and perspectives of cervical cancer screening (CCS). |
| Outcomes assessed | ☒Barriers – *(focus on 'perspectives' and 'experiences' capturing primarily barriers and some facilitators)*<br>☒Facilitators |
| Type of review | A review that seeks to include:<br>☒Qualitative studies<br>☐Quantitative studies |

Data extraction form (CERVICAL)

| | |
|---|---|
| | ☐Mixed methods studies<br>☐Not specified |
| Systematic review | Do the authors state this is a systematic review, systematic scoping review, or other form of systematic evaluation?<br>☒Yes<br>☐No |
| Screening programmes targeted by the review | ☐Bowel<br>☐Breast<br>☒Cervical |
| Focus on specific method of screening? | N/A |
| Details of included studies | |
| Population(s) of the included reviews | Included characteristics:<br>☒Age: Inclusion criteria for studies was 21-70 years old<br>☒Age within screening for NHS Scotland (25-64)?:<br>☒Gender and/or sex (described by the authors): Women<br>☒Ethnicity: Black/African American<br>☒Other: Two studies focused on HIV-positive women (one low-income study); one study focused on black lesbian, bisexual, and queer women; one study focused on women with high school education or less and a yearly income <$35,000 USD |
| Number of studies included | 17 |
| Total number of participants across all studies | 591 |
| Type of studies included | ☒Qualitative studies - 17<br>☐Quantitative studies<br>☐Mixed methods studies<br>☐Not specified |
| Geographic scope (high-income countries only) | USA |
| Search strategy and methods | |
| Sources | Ovid MEDLINE, Ovid Embase, Ovid PsycINFO, EBSCO Cumulative Index to Nursing and Allied Health Literature (CINAHL), PubMed, and the Social Sciences and Humanities segments in Scopus. |
| Search restrictions (language, years, region etc) | Search strategy peer-reviewed using the PRESS criteria with the main concept relevant to cervical cancer screening<br>Full-text search strategy available in Appendix 1 of the supporting CADTH report<br>Search originally conducted February 6, 2017; monthly search updates were performed until March 18, 2020 |
| Search dates | Limited to papers published since January 02, 2002 |
| Other criteria | Grey literature was included and some hand-searching of eight eligible papers was also conducted. |
| Instrument/tool used for quality appraisal of studies | ☒Yes: Critical Appraisal Skills Programme (CASP) Qualitative Checklist<br>☐Not specified |
| Characteristics of included studies table? | ☒Yes: Table 2<br>☐No |

Data extraction form (CERVICAL)

| | |
|---|---|
| Method of analysis/synthesis of results | ☐Narrative synthesis<br>☐Thematic analysis<br>☒Other qualitative analysis: Qualitative meta-synthesis<br>☐Meta-analysis<br>☐Other quantitative analysis |
| Presentation of results supported by | ☐Tabulation<br>☐Framework/model<br>☐Forest plot<br>☒Other: Conceptual diagram |
| Key findings | |
| Key barriers and/or facilitators identified | Twelve factors related to accessing cervical screening were identified across three themes: individual, contemporary-socio cultural, and socio-historical and structural<br><br>Individual: competing priorities and responsibilities, financial barriers, experiences of discrimination in the clinical setting, fear of diagnosis, a history of trauma<br><br>Contemporary-socio cultural: (mis)understanding of CCS (cultural expectation that women will encourage others to attend screening), social stigma, religiosity/fatalism, mistrust in the health care system, and social and familial expectations; recommendations of a health care provider “carried considerable weight in women’s decision-making about engagement with CCS”<br><br>Socio-historical and structural factors: planning and neighbourhood design, the underrepresentation of Black health care providers<br><br>See: Results |
| Limitations of the review | – Small sample sizes recruited from specific organisations such as domestic assault shelters, STD clinics, and public Medicaid programs which may limit the generalisability of findings to other groups of Black women<br>– Studies exhibit a broad age range making it challenging to differentiate the experiences of women in differing age cohorts<br>– Studies do not capture the diversity of Black women – participants of the included studies tended to share similar social identities (cis-gendered, heterosexual, able-bodied, born in the United States…) |
| Implications for future research | – Experiences of Black women beyond the United States should be further explored as the results from the review are not wholly generalisable<br>– “Being attuned to the historical and contemporary realities of sexual, physical, and psychological violence experienced by Black women (particularly how different elements of the screening process may trigger responses to earlier experiences for survivors of sexual trauma) can help clinicians to provide care that empowers and centers the preferences of |

| | |
|---|---|
| | the survivor. Such actions can create a clinical environment that feels compassionate and safe for Black women who are survivors of trauma. This may encourage more Black women to get screened earlier, effectively improving patient outcomes."<br>– Lessening the burden placed on Black women in obtaining screening such as providing bus tickets, on-site childcare, and pre-scheduling appointments<br>– Clinicians must be aware of histories of clinical racism; "Elements of trauma-informed care, such as collaboration and empowerment, can not only help foster trust, but also encourage women to continue putting their health first." |
| Other notes | N/A |
| **Other** | |
| Quality appraisal (JBI checklist) | ☒Include<br>☐Exclude |

| Review details | |
|---|---|
| Study ID (First author, year) | Connolly (2020) |
| Title of the review | Barriers and facilitators to cervical cancer screening among transgender men and non-binary people with a cervix: A systematic narrative review. |
| Review objectives/focus of the review | A systematic review intending to synthesis published literature on cervical cancer screening among eligible gender minorities, aiming to identify barriers and facilitators that might inform changes in UK policy and practice. |
| Outcomes assessed | ☒Barriers *(barriers and facilitators noted in title of article)*<br>☒Facilitators |
| Type of review | A review that seeks to include:<br>☒Qualitative studies<br>☒Quantitative studies<br>☒Mixed methods studies<br>☐Not specified |
| Systematic review | Do the authors state this is a systematic review, systematic scoping review, or other form of systematic evaluation?<br>☒Yes<br>☐No |
| Screening programmes targeted by the review | ☐Bowel<br>☐Breast<br>☒Cervical |
| Focus on specific method of screening? | N/A |
| **Details of included studies** | |
| Population(s) of the included reviews | Included characteristics:<br>☒Age: Not reported in full for all studies, age ranges between 18 and 66+<br>☒Age within screening for NHS Scotland (25-64)?<br>☒Gender and/or sex (described by the authors): Transgender men and non-binary people with a cervix (note: descriptions may |

| | |
|---|---|
| | vary by individual study author[s] description of gender)<br>☐Ethnicity: Not reported<br>☒Other: Two studies included healthcare providers; on described as "healthcare providers who have performed a pap test on a transmasculine person" |
| Number of studies included | 27 |
| Total number of participants across all studies | 800,591 |
| Type of studies included | ☒Qualitative studies - 4<br>☒Quantitative studies - 20<br>☒Mixed methods studies - 3<br>☐Not specified |
| Geographic scope (high-income countries only) | CAN; FRN; ITA; USA |
| Search strategy and methods | |
| Sources | Medline, Embase, PsycINFO and Global Health databases |
| Search restrictions (language, years, region etc) | See Table 1 for the full search strategy |
| Search dates | From their date of inception until 3rd January 2020 |
| Other criteria | The references of any review articles that were identified by the database search were hand-searched for additional records. |
| Instrument/tool used for quality appraisal of studies | ☒Yes: CASP checklist and Mixed Methods Appraisal Tool – see Tables 4-5(c)<br>☐Not specified |
| Characteristics of included studies table? | ☒Yes: see Table 2 and Table 3<br>☐No |
| Method of analysis/synthesis of results | ☒Narrative synthesis<br>☐Thematic analysis<br>☐Other qualitative analysis<br>☐Meta-analysis<br>☐Other quantitative analysis |
| Presentation of results supported by | ☐Tabulation<br>☐Framework/model<br>☐Forest plot<br>☒Other: Narrative synthesis only |
| Key findings | |
| Key barriers and/or facilitators identified | Lack of knowledge surrounding the relationship between gender minority status and cervical cancer risk among both service users and providers and highlighted significant barriers to access for gender minorities AFAB.<br><br>Cervical cancer screening was not universally associated with dysphoria among gender minorities AFAB; providers should explore patients' preferences around screening, while avoiding assumptions.<br><br>Providers should be proficient in examination techniques that maximise patient autonomy and minimise gender dysphoria or pain. |

| | |
|---|---|
| | Self-swabs for high-risk HPV may provide a more acceptable, evidence-based, alternative to Pap smears.<br><br>See: Abstract, results |
| Limitations of the review | – Included studies primarily conducted in the USA; may not be generalizable in terms of wider geography (e.g., USA insurance-based healthcare system differs from that of the UK and other countries with universal healthcare)<br>– Differing geographic screening guidelines can also result in variations in care and access pathways to screening services<br>– Ability of some USA physicians to refuse care to gender minority people on religious grounds may also result in a lower uptake of CCS by gender minorities AFAB in comparison to other geographic regions<br>– Quantitative studies may also not be wholly representative, given the 'hidden' nature of the gender minority community |
| Implications for future research | – "Research examining the attitudes to cervical cancer screening among UK-based gender minorities AFAB is urgently needed, with a focus on provider and location preferences, and the acceptability of centralised National Health Service (NHS) databases that may be used to generate automatic invitations for screening."<br>– There is also an "urgent need for basic education surrounding the healthcare needs of gender minority patients AFAB, so that clinicians are responsive to each individual's needs and skilled in a range of approaches to cervical cancer screening."<br>– Policy documents are still lacking in the UK that further support gender minorities<br>– Clinicians are encouraged to present a variety of techniques for screening to maximise patient autonomy |
| Other notes | N/A |
| Other | |
| Quality appraisal (JBI checklist) | ☒Include<br>☐Exclude |

| Review details | |
|---|---|
| Study ID (First author, year) | Cudjoe (2021) |
| Title of the review | Understanding the Pap Testing Behaviors of African Immigrant Women in Developed Countries: A Systematic Review |
| Review objectives/focus of the review | To review studies on the prevalence and determinants of Pap testing among African Immigrant (AI) women living in developed countries. |
| Outcomes assessed | ☒Barriers *(focus on behaviours or 'determinants' of participation in a screening programme, relevant to both barriers and facilitators)*<br>☒Facilitators |
| Type of review | A review that seeks to include:<br>☒Qualitative studies<br>☒Quantitative studies |

| | |
|---|---|
| | ☒Mixed methods studies<br>☐Not specified |
| Systematic review | Do the authors state this is a systematic review, systematic scoping review, or other form of systematic evaluation?<br>☒Yes<br>☐No |
| Screening programmes targeted by the review | ☐Bowel<br>☐Breast<br>☒Cervical |
| Focus on specific method of screening? | Papanicolaou test |
| Details of included studies | |
| Population(s) of the included reviews | Included characteristics:<br>☒Age: Noted by authors as ranging from 18-75 years<br>☒Age within screening for NHS Scotland (25-64)?<br>☒Gender and/or sex (described by the authors): Women<br>☒Ethnicity: African; only five studies identified country of origin, consisting primarily of Somalis<br>☒Other: Immigrants |
| Number of studies included | 16 |
| Total number of participants across all studies | 3,605,235 |
| Type of studies included | ☒Qualitative studies<br>☒Quantitative studies<br>☒Mixed methods studies<br>☐Not specified – see author notes: 12 cross-sectional design; 2 retrospective cohort; 1 mixed methods; 1 randomised controlled trial |
| Geographic scope (high-income countries only) | AUS; CAN; ESP; FIN; ITA; NOR; USA |
| Search strategy and methods | |
| Sources | PubMed, CINAHL, Embase, Scopus |
| Search restrictions (language, years, region etc) | Use of MeSH subject headings and free text search<br>Focus on AI women in developed countries with correlating Pap testing behaviour<br>*Search strategy included as Appendix 1 |
| Search dates | Search conducted in Dec 2018; 10 year scope |
| Other criteria | Hand searching also conducted of relevant articles for those not indexed by the electronic databases used; non-English articles excluded at time of study selection and data extraction |
| Instrument/tool used for quality appraisal of studies | ☒Yes: JBI Checklists<br>☐Not specified |
| Characteristics of included studies table? | ☒Yes: Table 2<br>☐No |
| Method of analysis/synthesis of results | ☐Narrative synthesis<br>☒Thematic analysis - Andersen Behavioral Model used to inform synthesis<br>☐Other qualitative analysis |

| | |
|---|---|
| | ☐Meta-analysis<br>☐Other quantitative analysis |
| Presentation of results supported by | ☒Tabulation<br>☐Framework/model<br>☐Forest plot<br>☐Other |
| Key findings | |
| Key barriers and/or facilitators identified | Having access to a primary care provider and female gender of the provider facilitate Pap testing among AI women, which is consistent with results from studies on Hispanic and Asian immigrants<br><br>Evidence from more than one study showed that low income, male healthcare providers, and no history of gynaecological exam (i.e., first time screeners) were barriers to Pap testing<br><br>See: Results, discussion |
| Limitations of the review | – Limited use of guiding theory within included studies, limiting the generalisability of findings<br>– Participants within studies may have been incorrectly identified as AI and varying global testing guidelines for routine Pap make generalisability difficult across age spectrums<br>– Research focused heavily on psychosocial barriers to uptake, where known predictors such as health literacy were less cited; psychometric measurement tools were rarely used to support psychosocial findings<br>– A priori power analysis was undertaken only in one study; possible that the rest of the studies were underpowered |
| Implications for future research | – "Our study findings indicate the need for theory-guided, methodologically rigorous studies that use psychometrically tested instruments, utilize various forms of recruitment strategies (i.e. engage ethnic churches and organizations), and include larger samples of diverse groups of African immigrants (i.e. different African nationalities) to address the Pap testing behaviors and cancer health needs of AI women".<br>– "..further research is also needed to help clinicians and researchers gain an in-depth understanding of why male healthcare providers may be barriers to Pap testing behaviors among AI women in developed countries." |
| Other notes | N/A |
| Other | |
| Quality appraisal (JBI checklist) | ☒Include<br>☐Exclude |

| Review details | |
|---|---|
| Study ID (First author, year) | Ferdous (2018) |
| Title of the review | Barriers to cervical cancer screening faced by immigrant women in Canada: a systematic scoping review |

Data extraction form (CERVICAL)

| | |
|---|---|
| Review objectives/focus of the review | The objective of this scoping study is to review the published literature and summarize findings related to barriers experienced by immigrant women in Canada while accessing cervical cancer screening. |
| Outcomes assessed | ☒Barriers<br>☐Facilitators |
| Type of review | A review that seeks to include:<br>☒Qualitative studies<br>☒Quantitative studies<br>☒Mixed methods studies<br>☐Not specified |
| Systematic review | Do the authors state this is a systematic review, systematic scoping review, or other form of systematic evaluation?<br>☒Yes<br>☐No |
| Screening programmes targeted by the review | ☐Bowel<br>☐Breast<br>☒Cervical |
| Focus on specific method of screening? | N/A |
| Details of included studies | |
| Population(s) of the included reviews | Included characteristics:<br>☒Age: Variable when reported by study, range from 18-79<br>☒Age within screening for NHS Scotland (25-64)?<br>☒Gender and/or sex (described by the authors): Women<br>☒Ethnicity: Authors note study populations were diverse, “with a large number of selected studies involving Asian immigrant women”<br>☒Other: Immigrants; low socioeconomic status and low income were significant barriers across multiple studies |
| Number of studies included | 28 |
| Total number of participants across all studies | 3,728,476 |
| Type of studies included | ☒Qualitative studies - 12<br>☒Quantitative studies - 9<br>☒Mixed methods studies - 7<br>☐Not specified |
| Geographic scope (high-income countries only) | CAN |
| Search strategy and methods | |
| Sources | See Table 1 |
| Search restrictions (language, years, region etc) | See Table 2 |
| Search dates | Not stated |
| Other criteria | Inclusive of grey literature and reference list searching of the final 28 articles for inclusion |
| Instrument/tool used for quality appraisal of studies | ☐Yes:<br>☒Not specified |

| | |
|---|---|
| Characteristics of included studies table? | ☒Yes: See Table 3<br>☐No |
| Method of analysis/synthesis of results | ☐Narrative synthesis<br>☒Thematic analysis<br>☐Other qualitative analysis<br>☐Meta-analysis<br>☐Other quantitative analysis |
| Presentation of results supported by | ☒Tabulation<br>☐Framework/model<br>☐Forest plot<br>☐Other: |
| Key findings | |
| Key barriers and/or facilitators identified | Based on this review, knowledge-related barriers were shown to be one of the biggest challenges for both patients and healthcare providers, and women's knowledge of cervical cancer and its screening was significantly affected by other barriers.<br><br>Cultural taboos around sexuality and sexual health also discouraged immigrant women from seeking more information concerning cervical cancer and screening.<br><br>Women who were cared for by female physicians reported being more comfortable discussing this subject, and as a result, had more knowledge compared to women who received healthcare from a male doctor.<br><br>Immigrant women's knowledge was also influenced by their linguistic weakness, which significantly affected both communication skills and their ability to understand the available information on cervical cancer screening.<br><br>Many cultural and religious beliefs (e.g., disease is up to God), alongside a low risk perception of cancer, failure to consider screening as priority, fear of cancer and its prognosis, and fear of the Pap test procedure itself were shown to be significantly associated with a general lack of knowledge about cervical cancer and its detection.<br><br>Despite the religious taboo and social stigma associated with cervical cancer and screening, women from many different cultural backgrounds respected physicians' opinions and recommendations.<br><br>See: Results |
| Limitations of the review | – Search limited to English-language results, not inclusive of French Canadian viewpoints<br>– Limitations on population criteria excluded refugees, the undocumented, and temporary migrants; ability to extrapolate findings to these groups is limited<br>– Limited applicability to healthcare systems beyond Canada given the scope of the inclusion/exclusion criteria; however, |

| | |
|---|---|
| | broader findings related to immigrants likely to be relevant in other settings |
| Implications for future research | – "…cancer screening-related information needs to be distributed and communicated in a culturally sensitive and linguistically appropriate manner. A proper explanation of the test procedure by physicians can help patients feel more comfortable and can alleviate their fears".<br>– "Providing access to female healthcare providers for all immigrant women is challenging, but establishing culturally sensitive screening programs that provide timely access, particularly in immigrant-dense areas, can offer an effective solution."<br>– There is a need to ensure physician knowledge of guidelines and recommendation for screening is up-to-date with adequate incentives for screening to be completed; "The present of an effective reminder system for the primary care team and for patients can also be helpful"<br>– Pathways to familiarize new immigrants with the healthcare system should be further investigated to ensure a preventative health focus<br>– Future studies should focus on culture and social barriers for defined ethnic groups (no 'one-size-fits-all' approach) |
| Other notes | N/A |
| Other | |
| Quality appraisal (JBI checklist) | ☒Include<br>☐Exclude |

| Review details | |
|---|---|
| Study ID (First author, year) | Hendry (2012) |
| Title of the review | Are women ready for the new cervical screening protocol in England? A systematic review and qualitative synthesis of views about human papillomavirus testing |
| Review objectives/focus of the review | This is the first review to synthesise a substantial body of international evidence of women's information needs, views and preferences regarding HPV testing. We aimed to inform the development of educational materials to promote informed choice, reduce anxiety and improve disease control. |
| Outcomes assessed | ☒Barriers *(describe as negative and positive associations/concerns)*<br>☒Facilitators |
| Type of review | A review that seeks to include:<br>☒Qualitative studies<br>☐Quantitative studies<br>☒Mixed methods studies<br>☐Not specified |
| Systematic review | Do the authors state this is a systematic review, systematic scoping review, or other form of systematic evaluation?<br>☒Yes<br>☐No |

Data extraction form (CERVICAL)

| | |
|---|---|
| Screening programmes targeted by the review | ☐Bowel<br>☐Breast<br>☒Cervical |
| Focus on specific method of screening? | N/A |
| Details of included studies | |
| Population(s) of the included reviews | Included characteristics:<br>☒Age: Youngest age reported in included studies was 14; highest range to 83 years<br>☒Age within screening for NHS Scotland (25-64)?<br>☒Gender and/or sex (described by the authors): Women<br>☒Ethnicity: Variable by study when reported; as noted in Table 2<br>☒Other: Authors note studies overall incorporate socioeconomic factors such as level of education, low income, and recruitment in 'poor areas' |
| Number of studies included | 17 |
| Total number of participants across all studies | 2,876; one study did not report the number of participants |
| Type of studies included | ☒Qualitative studies - 16<br>☐Quantitative studies<br>☒Mixed methods studies - 1<br>☐Not specified |
| Geographic scope (high-income countries only) | AUS; CAN; GBR; USA |
| Search strategy and methods | |
| Sources | 12 electronic databases; see Appendix 1 for full list |
| Search restrictions (language, years, region etc) | See Table 1 for inclusion and exclusion criteria |
| Search dates | Searches were conducted in June 2009, updated in July 2010, and limited to 1980 onwards, when relevant papers began to appear. |
| Other criteria | Bibliographies of included studies were also hand-searched. |
| Instrument/tool used for quality appraisal of studies | ☒Yes: NICE (2009) – Methodology checklist: qualitative studies<br>☐Not specified |
| Characteristics of included studies table? | ☒Yes: See Table 2<br>☐No |
| Method of analysis/synthesis of results | ☐Narrative synthesis<br>☒Thematic analysis<br>☐Other qualitative analysis<br>☐Meta-analysis<br>☐Other quantitative analysis |
| Presentation of results supported by | ☐Tabulation<br>☐Framework/model<br>☐Forest plot<br>☒Other: Illustrative quotations for each theme |
| Key findings | |
| Key barriers and/or facilitators identified | Three thematic categories emerged: the psychosocial burden of HPV infection, the acceptability of HPV testing and triage and information needs. |

| | |
|---|---|
| | Notably, the first was much more prominent in 'real' situations where participants expressed overwhelmingly negative concerns, suggesting that facing an HPV diagnosis in real life, with the associated problems of disclosure and fear of stigmatisation, was more daunting than women imagined in hypothetical scenarios.<br><br>Many women in the studies of HPV testing as a hypothetical proposal thought it a preferable alternative to repeat smear tests, but did not want to be tested without their knowledge or without informed consent.<br><br>Our review reveals an overwhelming lack of understanding about HPV with participants struggling to interpret limited information in the context of existing knowledge, impacting on the ability to make informed choices.<br><br>See: Discussion, summary of main findings |
| Limitations of the review | – No included studies explored the views of health professionals; no male participants, and no investigative views of LGBTQ+ groups, limiting generalizability of findings<br>– Survey components of qualitative studies were of poor quality |
| Implications for future research | – Further development of educational materials, particularly around terminology, to inform consent is needed; "Identification of the salient points allowing informed choice to uptake from this complex information remains a challenge to researchers…explanations about causation, risk of cervical abnormality or cancer, persistence/clearance of monogenic HPV and difference from genital wart viruses are needed" |
| Other notes | N/A |
| Other | |
| Quality appraisal (JBI checklist) | ☒Include<br>☐Exclude |

| Review details | |
|---|---|
| Study ID (First author, year) | Jillapalli (2022) |
| Title of the review | Cervical Cancer Screening Behaviors Among Asian Indians in the United States: A Systematic Review |
| Review objectives/focus of the review | The purpose of this systematic review is to examine factors influencing cervical cancer screening behaviors among [Asian Indians] in the US. |
| Outcomes assessed | ☒Barriers *(descriptive behaviours either seen to increase or decrease likelihood of screening uptake – described as barriers/facilitators in the 'results')*<br>☒Facilitators |
| Type of review | A review that seeks to include:<br>☐Qualitative studies<br>☒Quantitative studies<br>☐Mixed methods studies<br>☐Not specified |

Data extraction form (CERVICAL)

| | |
|---|---|
| Systematic review | Do the authors state this is a systematic review, systematic scoping review, or other form of systematic evaluation?<br>☒Yes<br>☐No |
| Screening programmes targeted by the review | ☐Bowel<br>☐Breast<br>☒Cervical |
| Focus on specific method of screening? | N/A |
| Details of included studies | |
| Population(s) of the included reviews | Included characteristics:<br>☒Age: Mean ages of the participants ranged from 30 to 50 years as noted by the authors<br>☒Age within screening for NHS Scotland (25-64)?<br>☒Gender and/or sex (described by the authors): Women<br>☒Ethnicity: Asian Indians (AI)<br>☒Other: Overall, 82–91% of the total participants had college educations and higher; 68.9 to 100% were employed; 55.5–73.7% had health insurance; Six studies surveyed healthcare professionals, including one study targeted nationwide at AI physicians |
| Number of studies included | 7 |
| Total number of participants across all studies | 20,095 |
| Type of studies included | ☐Qualitative studies<br>☒Quantitative studies - 7<br>☐Mixed methods studies<br>☐Not specified |
| Geographic scope (high-income countries only) | USA |
| Search strategy and methods | |
| Sources | Medline, CINAHL, PubMed, PsychINFO, and ProQuest databases |
| Search restrictions (language, years, region etc) | Search terms of “south asian indian*”, “cervical cancer screening”, “pap smears”, and “cervical screening guidelines” in various combinations; studies conducted in the North America, English language, first generation South Asian Indian population in the sample, and peer-reviewed research articles. |
| Search dates | All database searches included studies published between January 1990 and January 2020 using the Preferred Reporting Items for Systematic Reviews and Meta-Analyses (PRISMA) method. |
| Other criteria | The review focused on studies conducted within North America only in order to avoid the influence of healthcare delivery systems in other countries and immigration to a similar geographic location as confounding factors. |
| Instrument/tool used for quality appraisal of studies | ☒Yes: Fink (2005)<br>☐Not specified |
| Characteristics of included studies table? | ☒Yes: See Table 1<br>☐No |

| | |
|---|---|
| Method of analysis/synthesis of results | ☒Narrative synthesis<br>☐Thematic analysis<br>☐Other qualitative analysis<br>☐Meta-analysis<br>☒Other quantitative analysis – no true meta-analysis, but detailed information provided on rates of behaviours plus additional narrative organisation of key theoretical frameworks |
| Presentation of results supported by | ☒Tabulation: See Table 2<br>☐Framework/model<br>☐Forest plot<br>☐Other: |
| Key findings | |
| Key barriers and/or facilitators identified | Several barriers to cervical cancer screening among AIs in the US emerge from this systematic review. In particular, (1) cultural beliefs (none are explicitly stated in the studies; although participants referred to barriers to cervical cancer screening as their "culture," some studies include religion as a cultural factor), (2) marital status such as being unmarried and not being sexually active, (3) healthcare provider of the opposite sex, (4) lack of access to health care, regular health care provider, health insurance, time, and transportation, (5) language barriers, and (6) physician ethnicity.<br><br>Some of the common factors influencing cancer screening behaviors positively include (1) having lived in the US more than 10 years (effects of acculturation), (2) attaining higher education, (3) being employed, (4) holding beliefs such as benefits of early cancer screening, and importance of cancer screening, (5) being married, and (6) receiving physician recommendation.<br><br>See: Results |
| Limitations of the review | – A number of the earlier studies combined all Asian Americans into one group, causing data specific for AIs to be unobtainable.<br>– The current terminology used to describe Asian subgroups are mixed and confusing; tendency by researchers to 'bundle' all Asian subgroups.<br>– Although percentages of cervical cancer screening behaviors were provided, the usefulness and generalizability are limited due to use of convenience sampling, random sampling with small response rates, and heterogeneity of the samples.<br>– Limited data on socioeconomic status, education, health literacy, nativity, acculturation or recent immigration status; findings may also be relevant to peak periods of immigration in the United States, between 1990 and 2000. |
| Implications for future research | – The studies included for review found that future research should "focus on cultural beliefs that influence cervical cancer screening behaviour. [This] will better inform the development of culturally sensitive educational interventions |

| | |
|---|---|
| | for both the AI population and for health care providers that provide care to minority populations.” |
| Other notes | N/A |
| Other | |
| Quality appraisal (JBI checklist) | ☒Include<br>☐Exclude |

| Review details | |
|---|---|
| Study ID (First author, year) | Kandasamy (2021) |
| Title of the review | Indigenous women’s experiences of cervical cancer screening: Incorporating Indigenous ways of knowing into a systematic review and meta-synthesis of qualitative research |
| Review objectives/focus of the review | The research question for this investigation was: What are the perspectives, preferences, and experiences of Indigenous women about cervical cancer screening? |
| Outcomes assessed | ☒Barriers *(presented as ‘perspectives’; ‘preferences’; and ‘experiences’)*<br>☒Facilitators |
| Type of review | A review that seeks to include:<br>☒Qualitative studies<br>☐Quantitative studies<br>☐Mixed methods studies<br>☐Not specified |
| Systematic review | Do the authors state this is a systematic review, systematic scoping review, or other form of systematic evaluation?<br>☒Yes<br>☐No |
| Screening programmes targeted by the review | ☐Bowel<br>☐Breast<br>☒Cervical |
| Focus on specific method of screening? | N/A |
| Details of included studies | |
| Population(s) of the included reviews | Included characteristics:<br>☐Age: Not reported<br>☐Age within screening for NHS Scotland (25-64)? N/A<br>☒Gender and/or sex (described by the authors): Women<br>☒Ethnicity: Indigenous people (no further specifics reported by the authors)<br>☒Other: Some studies included the perspectives of healthcare providers who care for Indigenous women |
| Number of studies included | 9 |
| Total number of participants across all studies | Not reported |
| Type of studies included | ☒Qualitative studies - 9<br>☐Quantitative studies<br>☐Mixed methods studies<br>☐Not specified |

Data extraction form (CERVICAL)

| | |
|---|---|
| Geographic scope (high-income countries only) | AUS; CAN; USA |
| Search strategy and methods | |
| Sources | Ovid MEDLINE, Ovid Embase, Ovid PsycINFO, EBSCO Cumulative Index to Nursing and Allied Health Literature (CINAHL), PubMed, and the Social Sciences and Humanities segments in Scopus |
| Search restrictions (language, years, region etc) | The search terms combined a topic-specific search filter about CCS with a published search filter designed to retrieve qualitative research. |
| Search dates | Studies published since 01 Jan 2002; regularly updated until 16 Mar 2020. |
| Other criteria | Selected grey literature sources were also searched using guidance from the CADTH Grey Matters Checklist |
| Instrument/tool used for quality appraisal of studies | ☒Yes: See references to Sandelowski & Barroso (2003a) and Majid & Vanstone (2018)<br>☐Not specified |
| Characteristics of included studies table? | ☒Yes: See Tables 1 and 2 for brief overviews<br>☐No |
| Method of analysis/synthesis of results | ☒Narrative synthesis<br>☐Thematic analysis<br>☐Other qualitative analysis<br>☐Meta-analysis<br>☐Other quantitative analysis |
| Presentation of results supported by | ☐Tabulation<br>☐Framework/model<br>☐Forest plot<br>☒Other: Two Row Wampum-Covenant Chain Tradition, integrating Western approaches with Indigenous worldviews |
| Key findings | |
| Key barriers and/or facilitators identified | Barriers: distrust in the healthcare system, impact of colonial legacies (including experiences of abuse and racism), discomfort or embarrassment about invasive nature of Pap tests, distance to appointment (including lack of transportation), scheduling concerns (including childcare), CCS not considered a priority, screening causes harm through stigma, fear and stress over diagnosis<br><br>Facilitators: family relations (mother-daughter bonds), preference for self-screening, culturally competent healthcare (communication strategies designed for Indigenous women), providers who reflect the lived experiences of patients (*gender, language, and ethnicity preferences)<br><br>See: Findings, discussion |
| Limitations of the review | Not stated by the authors. |
| Implications for future research | – Emphasis on culturally competent healthcare and trauma-informed care approaches; training approach should be prioritised that encourages "cultural competency training through teachings of colonial legacies, issues related to the |

| | |
|---|---|
| | intersectionality of race, gender, and social class, and Indigenous understandings of health and wellbeing.”<br>– “Practice changes that increase the building of trust through culturally competent healthcare, engaging in Indigenous-led research, and prioritising Indigenous perspectives in the implementation of policies, can collaboratively improve barriers and reinstate the facilitators of CCS participation, cervical cancer treatment, and survival.” |
| Other notes | N/A |
| **Other** | |
| Quality appraisal (JBI checklist) | ☒Include<br>☐Exclude |

| Review details | |
|---|---|
| Study ID (First author, year) | Majid (2019) |
| Title of the review | Women’s preferences and experiences of cervical cancer screening in rural and remote areas: a systematic review and qualitative meta-synthesis |
| Review objectives/focus of the review | This systematic review and qualitative meta-synthesis of 14 studies aimed to describe and elaborate the issues women face when accessing cervical cancer screening in rural and remote areas. |
| Outcomes assessed | ☒Barriers *(described as ‘preferences’)*<br>☒Facilitators |
| Type of review | A review that seeks to include:<br>☒Qualitative studies<br>☐Quantitative studies<br>☐Mixed methods studies<br>☐Not specified |
| Systematic review | Do the authors state this is a systematic review, systematic scoping review, or other form of systematic evaluation?<br>☒Yes<br>☐No |
| Screening programmes targeted by the review | ☐Bowel<br>☐Breast<br>☒Cervical |
| Focus on specific method of screening? | N/A |
| **Details of included studies** | |
| Population(s) of the included reviews | Included characteristics:<br>☒Age: Noted exclusion criteria as studies including women ≥71 years of age<br>☒Age within screening for NHS Scotland (25-64)?<br>☒Gender and/or sex (described by the authors): Women<br>☐Ethnicity: Not reported<br>☒Other: At least four studies are referenced in the discussion in regard to distrust in clinicians who show stigma towards lower socioeconomic status; however, a detailed list of study specifics was not included by the authors |

Data extraction form (CERVICAL)

| | |
|---|---|
| Number of studies included | 14 |
| Total number of participants across all studies | 566 |
| Type of studies included | ☒Qualitative studies - 14<br>☐Quantitative studies<br>☐Mixed methods studies<br>☐Not specified |
| Geographic scope (high-income countries only) | CAN; NZL; USA (all rural) |
| Search strategy and methods | |
| Sources | Ovid Medline, Ovid Embase, Ovid PsycINFO, EBSCO Cumulative Index to Nursing and Allied Health Literature (CINAHL), PubMed and the Social Sciences and Humanities segments in Scopus. |
| Search restrictions (language, years, region etc) | Results were limited to English- and French-language publications. Conference abstracts were excluded from the search results. |
| Search dates | Limited to studies published since 1 January 2002. The search was conducted on 6 February 2017, with monthly search updates ensuring that the review was current to 1 June 2018. |
| Other criteria | Selected grey literature sources identified from the *Grey matters* checklist were also searched. |
| Instrument/tool used for quality appraisal of studies | ☒Yes: CASP checklist<br>☐Not specified |
| Characteristics of included studies table? | ☐Yes<br>☒No: See Tables 2 and 3 for summary of the number of included studies according to study design and study location (no further characteristics provided) |
| Method of analysis/synthesis of results | ☐Narrative synthesis<br>☐Thematic analysis<br>☐Other qualitative analysis<br>☐Meta-analysis<br>☒Other quantitative analysis: Qualitative meta-synthesis |
| Presentation of results supported by | ☒Tabulation: See Tables 2 and 3<br>☐Framework/model<br>☐Forest plot<br>☐Other: |
| Key findings | |
| Key barriers and/or facilitators identified | Barriers: time and location of CCS without considering the logistical obstacles faced by rural women (lack of transportation, time to attend commitment and competing priorities including childcare and work, inflexible appointment times), discrimination towards women due to rural culture, absence of continuity of care (may only practice in rural area for limited amount of time, taking few patients), limitations on personal privacy due to close-knit nature of rural communities, overall mistrust in the healthcare system<br><br>Facilitators: culturally competent healthcare, recommendation of a physician to attend screening<br><br>See: Results, discussion |

Data extraction form (CERVICAL)

| | |
|---|---|
| Limitations of the review | – Findings likely not transferrable outside of high-income country settings<br>– Variability in definitions of 'rurality'; "…does not mean that its primary focus was to describe the issues pertinent to rural health care and CCS" |
| Implications for future research | – Concerns surrounding cervical screening uptake "may be managed with increased access and availability to alternative [healthcare providers] who are perceived by the woman as having the capacity to relate to her situation and appreciate the various factors that complicate CCS participation (gender, race, class, language)" |
| Other notes | N/A |
| Other | |
| Quality appraisal (JBI checklist) | ☒Include<br>☐Exclude |

| | |
|---|---|
| Review details | |
| Study ID (First author, year) | Nagendiram (2020) |
| Title of the review | Australian women's self-perceived barriers to participation in cervical cancer screening: A systematic review |
| Review objectives/focus of the review | The primary aim was to complete a systematic literature review of the factors that prevent Australian women from participating in cervical screening. |
| Outcomes assessed | ☒Barriers<br>☐Facilitators |
| Type of review | A review that seeks to include:<br>☐Qualitative studies<br>☒Quantitative studies<br>☐Mixed methods studies<br>☐Not specified |
| Systematic review | Do the authors state this is a systematic review, systematic scoping review, or other form of systematic evaluation?<br>☒Yes<br>☐No |
| Screening programmes targeted by the review | ☐Bowel<br>☐Breast<br>☒Cervical |
| Focus on specific method of screening? | N/A – however, results include focused discussion on role of HPV self-screening kits |
| Details of included studies | |
| Population(s) of the included reviews | Included characteristics:<br>☒Age: Not consistently reported by studies; range from 18 to 70 years when reported.<br>☒Age within screening for NHS Scotland (25-64)?<br>☒Gender and/or sex (described by the authors): Women<br>☒Ethnicity: See 'Other'<br>☒Other: Four studies focused on migrant women from a variety of European and Asian backgrounds; 1 study focused on women who have sex with other women; 3 studies incorporate older |

| | |
|---|---|
| | women, up to 70 years of age; 1 study focused on bone-marrow transplant survivors; 2 studies focused on women with a history of mental illness or sexual assault; 1 study included a rural or remote sample |
| Number of studies included | 13 |
| Total number of participants across all studies | 7,265 |
| Type of studies included | ☐Qualitative studies<br>☒Quantitative studies - 13<br>☐Mixed methods studies<br>☐Not specified |
| Geographic scope (high-income countries only) | AUS (incl some rural) |
| **Search strategy and methods** | |
| Sources | CINAHL, Medline, SCOPUS and the Cochrane Library |
| Search restrictions (language, years, region etc) | Quantitative studies published in peer-reviewed journals. |
| Search dates | Studies after 1991 were considered. Final search of databases conducted on 27 Jul 2018. |
| Other criteria | Full search details available in Appendix 1 |
| Instrument/tool used for quality appraisal of studies | ☒Yes: National Heart, Lung and Blood Institute Quality Assessment Tool for observational studies<br>☐Not specified |
| Characteristics of included studies table? | ☒Yes: See Table 1<br>☐No |
| Method of analysis/synthesis of results | ☒Narrative synthesis<br>☐Thematic analysis<br>☐Other qualitative analysis<br>☐Meta-analysis<br>☐Other quantitative analysis |
| Presentation of results supported by | ☒Tabulation: See Table 3 for a summary of barriers for cervical screening by participant grouping<br>☐Framework/model<br>☐Forest plot<br>☐Other: |
| **Key findings** | |
| Key barriers and/or facilitators identified | The most commonly stated barriers included lack of time, embarrassment, fear of results, irrelevance and male health professionals<br><br>The use of HPV triage in cervical screening was not a barrier to screening, however, some women regarded self-collected HPV testing as a barrier. Barriers to self-collection included desire for the general practitioner to complete the test, fear of doing the test incorrectly, wishing to include it in a general check-up and concerns about the test itself<br><br>See: Abstract, discussion |

| | |
|---|---|
| Limitations of the review | – Varying subgroups within the included studies generated unique barriers to screening that adds to heterogeneity across results.<br>– Results were not reflective of Indigenous Australian populations; samples may also not be wholly representative of the population under investigation due to varying methodologies.<br>– Inconsistencies in the presentation of results; no universal system to classify barriers to screening. |
| Implications for future research | – "General practitioners should offer opportunistic patient education and screening for women eligible to screening and facilitate conversations with under-screened women to overcome the barriers that prevent their participation […] clinicians should also be offered education regarding screening guidelines in specific subgroups [including WSW and BMT survivors]."<br>– Revisions to policy [in the Australian context] should also include further research and strategy development for WSW, older women, and migrant groups in addition to Aboriginal and Torres Strait Island women, immune-deficient women and women who have experienced sexual abuse |
| Other notes | Authors also acknowledge the review is subject to publication bias due to the review design, as only published studies were assessed. |
| Other | |
| Quality appraisal (JBI checklist) | ☒Include<br>☐Exclude |

| Review details | |
|---|---|
| Study ID (First author, year) | Nothacker (2022) |
| Title of the review | Women's attitudes towards a human papillomavirus-based cervical cancer screening strategy: a systematic review |
| Review objectives/focus of the review | Changing cervical cancer screening from cytology to HPV-based screening could influence the acceptability and thus the overall success of screening programmes. Understanding women's attitudes towards an HPV-based screening strategy is therefore essential for the development of successful screening and implementation strategies. |
| Outcomes assessed | ☒Barriers *(focus on positive/negative attitudes that influence uptake of screening services)*<br>☒Facilitators |
| Type of review | A review that seeks to include:<br>☒Qualitative studies<br>☒Quantitative studies<br>☐Mixed methods studies<br>☐Not specified |
| Systematic review | Do the authors state this is a systematic review, systematic scoping review, or other form of systematic evaluation? |

Data extraction form (CERVICAL)

| | |
|---|---|
| | ☒Yes<br>☐No |
| Screening programmes targeted by the review | ☐Bowel<br>☐Breast<br>☒Cervical |
| Focus on specific method of screening? | HPV-based screening (self-test, or other in-office procedure) |
| Details of included studies | |
| Population(s) of the included reviews | Included characteristics:<br>☒Age: Variable when reported by study; lowest range age 16, highest reported range age >66<br>☒Age within screening for NHS Scotland (25-64)?<br>☒Gender and/or sex (described by the authors): Women<br>☒Ethnicity: Variable by study when reported; almost all studies included multiple ethnicities within the reported population<br>☒Other: 9 studies reported details about relationship status |
| Number of studies included | 12 |
| Total number of participants across all studies | 9,928 |
| Type of studies included | ☒Qualitative studies - 5<br>☒Quantitative studies - 7<br>☐Mixed methods studies<br>☐Not specified |
| Geographic scope (high-income countries only) | AUS; CAN; GBR; USA |
| Search strategy and methods | |
| Sources | Medline, Web of Science Core Collection, Cochrane Library, PsycINFO and CINAHL |
| Search restrictions (language, years, region etc) | See Supplemental material (S1) |
| Search dates | Initial searches took place in November 2019 and an update search in Medline was performed in August 2021; exact dates of years searched unclear from the described methodology. |
| Other criteria | Reference lists of eligible studies and systematic reviews were reviewed to identify any other studies that might not have been retrieved by the electronic searches.<br><br>Searches for ongoing or unpublished but completed studies were performed in ClinicalTrials.gov.<br><br>The 'similar articles' function was used in PubMed to retrieve additional references in addition to forward citation tracking using the Web of Science Core Collection. |
| Instrument/tool used for quality appraisal of studies | ☒Yes: Mixed Methods Appraisal Tool (MMAT)<br>☐Not specified |
| Characteristics of included studies table? | ☒Yes: See Table 1<br>☐No |
| Method of analysis/synthesis of results | ☐Narrative synthesis<br>☒Thematic analysis |

| | |
|---|---|
| | ☐Other qualitative analysis<br>☐Meta-analysis<br>☐Other quantitative analysis |
| Presentation of results supported by | ☒Tabulation<br>☐Framework/model<br>☐Forest plot<br>☐Other: |
| Key findings | |
| Key barriers and/or facilitators identified | Women's attitudes towards HPV-based screening strategies were mainly affected by the understanding of (i) the personal risk of an HPV infection, (ii) the implication of a positive finding and (iii) the overall screening purpose.<br><br>Women who considered their personal risk of HPV to be low and women who feared negative implications of a positive finding (including cultural or religious beliefs) were more likely to express negative attitudes, whereas positive attitudes were particularly expressed by women understanding the screening purpose.<br><br>Women with negative attitudes towards HPV-based screening particularly fear that being tested for a sexually transmitted infection may lead to stigmatisation. On the other hand, women with positive attitudes value the advantages of (potential) detection of earlier disease and a lower test frequency. |
| Limitations of the review | – Study limited to high-income countries, limiting generalisability of findings |
| Implications for future research | – Educational strategies should be targeted towards both men and women when considering STDs including key messaging around HPV infection; "This strategy should further explain changes in the screening procedure, including longer screening intervals and delayed age at first screening, and their consequences for the detection of cervical cancer."<br>– Education strategies should further explore how to communicate the meaning of test results; "information about the meaning of a positive HPV test and the prevalence of HPV among the population should also be included in education strategies." |
| Other notes | N/A |
| Other | |
| Quality appraisal (JBI checklist) | ☒Include<br>☐Exclude |

| Review details | |
|---|---|
| Study ID (First author, year) | Wearn (2022) |
| Title of the review | Determinants of routine cervical screening participation in underserved women: a qualitative systematic review |
| Review objectives/focus of the review | Routine, population-wide cervical screening programmes reduce cervical cancer incidence and mortality. However, socioeconomically deprived communities and ethnic minority groups typically have lower uptake in comparison to the general |

| | |
|---|---|
| | population and thus are described as 'underserved.' A systematic qualitative literature review was conducted to identify relevant determinants of participation for these groups. |
| Outcomes assessed | ☒Barriers *(focus on psychosocial elements of screening uptake)*<br>☒Facilitators |
| Type of review | A review that seeks to include:<br>☒Qualitative studies<br>☐Quantitative studies<br>☐Mixed methods studies<br>☐Not specified |
| Systematic review | Do the authors state this is a systematic review, systematic scoping review, or other form of systematic evaluation?<br>☒Yes<br>☐No |
| Screening programmes targeted by the review | ☐Bowel<br>☐Breast<br>☒Cervical |
| Focus on specific method of screening? | N/A |
| Details of included studies | |
| Population(s) of the included reviews | Included characteristics:<br>☐Age: Not reported for included studies; age range for population-wide screening programmes listed by country in supplemental material<br>☐Age within screening for NHS Scotland (25-64)? N/A<br>☒Gender and/or sex (described by the authors): Women<br>☒Ethnicity: Ethnic minority women, with a focus on migrant women<br>☒Other: 21 studies focused on ethnic minority women exclusively; 2 studies focused on perspectives of women from deprived communities; 1 study incorporated both ethnic minority women and those of low socioeconomic status |
| Number of studies included | 24 |
| Total number of participants across all studies | 763 |
| Type of studies included | ☒Qualitative studies - 24<br>☐Quantitative studies<br>☐Mixed methods studies<br>☐Not specified |
| Geographic scope (high-income countries only) | AUS; FIN; GBR; NLD; NOR; SWE |
| Search strategy and methods | |
| Sources | Web of Science, Scopus, MEDLINE, CINAHL and PsycARTICLES databases |
| Search restrictions (language, years, region etc) | See Supplementary Materials C for an example search strategy |
| Search dates | Initial search conducted June 2018; repeated until January 2021. Exact years searched not provided. |

| | |
|---|---|
| Other criteria | Forward and backward citation searching was conducted on all included studies. The reference lists of full text articles were also hand searched for additional eligible literature. |
| Instrument/tool used for quality appraisal of studies | ☒Yes: Critical Appraisal Skills Programme (CASP) checklist<br>☐Not specified |
| Characteristics of included studies table? | ☒Yes: Supplementary Materials H<br>☐No |
| Method of analysis/synthesis of results | ☐Narrative synthesis<br>☐Thematic analysis<br>☒Other qualitative analysis: Framework Synthesis<br>☐Meta-analysis<br>☐Other quantitative analysis |
| Presentation of results supported by | ☐Tabulation<br>☒Framework/model: Conceptual framework, see Figure 2 and illustrative quotes shown in Table 1<br>☐Forest plot<br>☐Other: |
| Key findings | |
| Key barriers and/or facilitators identified | Individual themes – embarrassment, fear, risk beliefs, religious beliefs, prioritising competing demands (including childcare, forgetting to book appointments), perceived stigma, knowledge.<br><br>Social context – peer and family influence, barriers to communication, unfamiliarity with screening.<br><br>Healthcare environment – past experiences of screening and healthcare, sex of practitioner, interpersonal skills of practitioners, continuity of care, medical mistrust, practitioner endorsement, service accessibility.<br><br>Wider society – culture, media. |
| Limitations of the review | – Under-served populations are diverse, and some of the highlighted sociodemographic characteristics are not mutually exclusive (results not fully representative of wider populations of under-served women) |
| Implications for future research | – Review findings suggest that further action is needed to address structural barriers for underserved populations and the “role of policy makers and healthcare providers in ensuring underserved women feel safe, supported and able to participate in cervical screening services”.<br>– There is also a need to prioritise culturally sensitive communication strategies and tools, particularly in the UK context given evidence within the review that “UK health services often do not meet the needs of culturally diverse groups.”<br>– Future qualitative research should focus on those living in areas of high deprivation as “…developing this body of evidence would allow for further exploration of observed uptake inequalities and encourage identification of suitable targets for intervention.” |

| | |
|---|---|
| | – Screening participation is reliant on a number of differing factors; further research is needed on the importance of social determinant on health behaviours as related to cervical screening participation |
| Other notes | N/A |
| Other | |
| Quality appraisal (JBI checklist) | ☒Include<br>☐Exclude |

Data extraction form (BREAST & CERVICAL; BOWEL, BREAST & CERVICAL)

| Review details | |
|---|---|
| Study ID (First author, year) | Byrnes (2020) |
| Title of the review | Attitudes and perceptions of people with a learning disability, family carers, and paid care workers towards cancer screening programmes in the United Kingdom: A qualitative systematic review and meta-aggregation |
| Review objectives/focus of the review | Evidence suggests that people with a learning disability (PwLD) are less likely to attend cancer screening than the general population in the United Kingdom. The aim of this systematic review was to identify and synthesise qualitative studies reporting the attitudes and opinions of PwLD, family carers, and paid care workers towards national cancer screening programmes. |
| Outcomes assessed | ☒Barriers *(focus on 'attitudes' and 'opinions')*<br>☒Facilitators |
| Type of review | A review that seeks to include:<br>☒Qualitative studies<br>☐Quantitative studies<br>☐Mixed methods studies<br>☐Not specified |
| Systematic review | Do the authors state this is a systematic review, systematic scoping review, or other form of systematic evaluation?<br>☒Yes<br>☐No |
| Screening programmes targeted by the review | ☐Bowel<br>☒Breast<br>☒Cervical |
| Focus on specific method of screening? | N/A |
| Details of included studies | |
| Population(s) of the included reviews | Included characteristics:<br>☒Age: Inconsistent when reported across studies; ranged from lower limit of 20 years of age to upper limit of 69<br>☒Age within screening for NHS Scotland (50-70)?:<br>☒Gender and/or sex (described by the authors): Female and male<br>☐Ethnicity: Not reported<br>☒Other: Three studies focused exclusively on women with a learning disability (WwLD); all other studies incorporate perspectives of carers including nurses, general practitioners, and other specialist health providers, paid and family carers, and residential staff |
| Number of studies included | 11 |
| Total number of participants across all studies | 300 |
| Type of studies included | ☒Qualitative studies<br>☐Quantitative studies<br>☐Mixed methods studies<br>☐Not specified |
| Geographic scope (high-income countries only) | GBR |

Data extraction form (BREAST & CERVICAL; BOWEL, BREAST & CERVICAL)

| Search strategy and methods | |
|---|---|
| Sources | MEDLINE, CINAHL, PubMed, Scopus, and PsycInfo |
| Search restrictions (language, years, region etc) | No language restrictions were applied (assumed all papers would be written in English, the language of the United Kingdom). |
| Search dates | All searches were conducted in April 2018; re-searched in February 2019. Years searched not provided. |
| Other criteria | An unpublished grey literature search of MEDNAR and Google Scholar was conducted with the first 100 listed records being retained. |
| Instrument/tool used for quality appraisal of studies | ☒Yes: JBI Quality Appraisal Review Instrument (QARI) for qualitative studies<br>☐Not specified |
| Characteristics of included studies table? | ☒Yes: See Table S2 Summary of included studies<br>☐No |
| Method of analysis/synthesis of results | ☐Narrative synthesis<br>☐Thematic analysis<br>☒Other qualitative analysis – Meta-aggregation<br>☐Meta-analysis<br>☐Other quantitative analysis |
| Presentation of results supported by | ☒Tabulation – see Supplementary materials<br>☐Framework/model<br>☐Forest plot<br>☐Other: |
| **Key findings** | |
| Key barriers and/or facilitators identified | A key finding from this review is that the cervical and breast cancer screening programmes may be inadvertently excluding WwLD due to having poor health literacy.<br><br>Family and paid care workers should not have unfavourable opinion towards screening; influence the WwLD's subjective norms, which affect the likelihood of WwLD feeling supported to engage in cancer screening.<br><br>Anxiety was a significant emotion affecting the screening experience which was also associated with anticipated pain. This can greatly affect whether WwLD attend any subsequent appointments or screening invitations.<br><br>See: Discussion<br><br>Also see – Abstract (Conclusions): WwLD may not attend cancer screening due to fear, concerns over pain, and the potential influence of family carers and paid care workers. |
| Limitations of the review | – Focus only on UK populations and findings may not be generalisable to other countries.<br>– Level of learning disability severity was not accounted for in the majority of papers, so findings may not be generalisable to all WwLD. |
| Implications for future research | – Highlights a need for a multidisciplinary approach to encouraging screening for WwLD and "should involve WwLD, |

| | |
|---|---|
| | family carers, and paid care workers including screening staff and GPs to ensure all [are] educated on cancer screening for WwLD".<br>– "…the findings from the review suggest that the health care of WwLD needs to be proactive and person-centred throughout the cancer screening pathway. This can include modifications to the invitations process, to not rely on literacy alone and utilise various communication aids including Makaton, or through additional resources such as visual recordings."<br>– "Future research should concentrate on involving WwLD, family carers, and paid care workers to account for all three groups' attitudes and opinions together to obtain an in-depth understanding via qualitative methods." |
| Other notes | N/A |
| Other | |
| Quality appraisal (JBI checklist) | ☒Include<br>☐Exclude |

| Review details | |
|---|---|
| Study ID (First author, year) | Pariser (2022) |
| Title of the review | Barriers to Access for Cervical and Breast Cancer Screenings Among Female Latinx Migrant Farmworkers in the US: A Scoping Literature Review |
| Review objectives/focus of the review | Identification of barriers to cancer screening among female migrant farmworkers is needed to inform intervention development and healthcare policy. Thus, [the purpose of the review] was to systematically review and summarize findings from prior studies of barriers faced by Latinx migrant farmworkers in accessing cervical and breast cancer screenings in the US. |
| Outcomes assessed | ☒Barriers<br>☐Facilitators |
| Type of review | A review that seeks to include:<br>☐Qualitative studies<br>☐Quantitative studies<br>☐Mixed methods studies<br>☒Not specified - noted as 13 observational studies, and 6 interventional studies (no further details provided) |
| Systematic review | Do the authors state this is a systematic review, systematic scoping review, or other form of systematic evaluation?<br>☒Yes<br>☐No |
| Screening programmes targeted by the review | ☐Bowel<br>☒Breast<br>☒Cervical |
| Focus on specific method of screening? | N/A |
| Details of included studies | |

Data extraction form (BREAST & CERVICAL; BOWEL, BREAST & CERVICAL)

| | |
|---|---|
| Population(s) of the included reviews | Included characteristics:<br>☐Age: Not reported<br>☐Age within screening for NHS Scotland (50-70)?: N/A<br>☒Gender and/or sex (described by the authors): Female<br>☒Ethnicity: Latinx<br>☒Other: Migrant farm workers |
| Number of studies included | 19 |
| Total number of participants across all studies | 6,106; participant numbers unknown for one study, and not reported for one study (situational analysis) |
| Type of studies included | ☐Qualitative studies<br>☐Quantitative studies<br>☐Mixed methods studies<br>☒Not specified – noted as 13 observational studies, and 6 interventional studies (no further details provided) |
| Geographic scope (high-income countries only) | USA (rural) |
| Search strategy and methods | |
| Sources | MEDLINE and Embase databases |
| Search restrictions (language, years, region etc) | Publication dates through 24 Oct 2021 using keyword searching. |
| Search dates | Not reported. |
| Other criteria | Additional identification resulted from snowball sampling by reviewing citations in each selected article. |
| Instrument/tool used for quality appraisal of studies | ☐Yes:<br>☒Not specified |
| Characteristics of included studies table? | ☒Yes: See Table 1<br>☐No |
| Method of analysis/synthesis of results | ☐Narrative synthesis<br>☒Thematic analysis<br>☐Other qualitative analysis<br>☐Meta-analysis<br>☐Other quantitative analysis |
| Presentation of results supported by | ☒Tabulation<br>☐Framework/model<br>☐Forest plot<br>☒Other: Geographic map also included (see Fig 2) |
| Key findings | |
| Key barriers and/or facilitators identified | Common themes about barriers to cancer screening included: lack of cancer knowledge, cost/inability to pay, lack of health insurance coverage, cultural perceptions, fear, language difficulties, and transportation and time constraints.<br><br>See: Summary of findings |
| Limitations of the review | – Insights from the included studies may not be representative of the larger migrant farmworker population because results are restricted in selected communities.<br>– There were few interventions identified; future research should explore specific needs and barriers to obtaining breast |

| | |
|---|---|
| | and cervical cancer screening among female migrant farmworkers. |
| Implications for future research | – "Pre-intervention work should focus on resources the community already has in place, as well as additional barriers that may be very specific to that community".<br>– Barriers should be made further specific to unique communities; facilitators were not addressed in this review and may add further significant to reduce barriers and amplify care.<br>– "The results of this review might also contribute to the development of advocacy tools to aid in the development of healthcare and migrant policy, as well as inform the declaration of government fiscal resources, be it local, state, or federal". |
| Other notes | N/A |
| Other | |
| Quality appraisal (JBI checklist) | ☒Include<br>☐Exclude |

| | |
|---|---|
| Review details | |
| Study ID (First author, year) | Jun (2018) |
| Title of the review | Determinants of Cancer Screening Disparities Among Asian Americans: A Systematic Review of Public Health Surveys |
| Review objectives/focus of the review | A systematic analysis examining Asian Americans' breast, cervical, and colon cancer screening, focusing on empirical findings from large-scale public health surveys. |
| Outcomes assessed | ☒Barriers *(focus on 'determinants')*<br>☒Facilitators |
| Type of review | A review that seeks to include:<br>☐Qualitative studies<br>☐Quantitative studies<br>☐Mixed methods studies<br>☒Not specified: National state health survey data from the USA used for all studies, no further details provided |
| Systematic review | Do the authors state this is a systematic review, systematic scoping review, or other form of systematic evaluation?<br>☒Yes<br>☐No |
| Screening programmes targeted by the review | ☒Bowel<br>☒Breast<br>☒Cervical |
| Focus on specific method of screening? | N/A |
| Details of included studies | |
| Population(s) of the included reviews | Included characteristics:<br>☒Age: Inconsistently reported across studies; lowest age limit of 18, upper limit for some studies of 75<br>☒Age within screening for NHS Scotland (50-70)?:<br>☒Gender and/or sex (described by the authors): Women and |

| | |
|---|---|
| | men<br>☒Ethnicity: Asian Americans<br>☒Other: Authors note that overall "relatively higher status in education and household income did not equate to more engagement in cancer screening". |
| Number of studies included | 24 |
| Total number of participants across all studies | 51,631; refers to Asian Americans only, authors note wider datasets may include additional ethnicities, participant numbers not reported for four studies. |
| Type of studies included | ☐Qualitative studies<br>☐Quantitative studies<br>☐Mixed methods studies<br>☒Not specified: National state health survey data from the USA used for all studies, no further details provided |
| Geographic scope (high-income countries only) | USA |
| Search strategy and methods | |
| Sources | PubMed, MEDLINE, CINAHL, PsycINFO, Scopus, Health Source: Nursing/Academic Edition, Academic Search Complete, Health Policy Reference Center, Psychology and Behavioral Sciences Collection, SocINDEX, and Women's Studies International. |
| Search restrictions (language, years, region etc) | Keyword searching. |
| Search dates | Articles published from 2000 to 2016. |
| Other criteria | References of searched articles were used to retrieve more relevant literature. |
| Instrument/tool used for quality appraisal of studies | ☐Yes:<br>☒Not specified |
| Characteristics of included studies table? | ☒Yes: See Table 1<br>☐No |
| Method of analysis/synthesis of results | ☒Narrative synthesis<br>☐Thematic analysis<br>☐Other qualitative analysis<br>☐Meta-analysis<br>☐Other quantitative analysis |
| Presentation of results supported by | ☒Tabulation<br>☐Framework/model<br>☐Forest plot<br>☐Other: |
| Key findings | |
| Key barriers and/or facilitators identified | Acculturation and healthcare access were two significant factors in explaining Asian Americans' cancer screening rates. Health literacy, cancer fatalism and family cancer history emerged as potential factors that may account for more variances.<br><br>See: Conclusions |
| Limitations of the review | – Some studies may have been missed as a result of the search process; data is limited to public health surveys. |

| | |
|---|---|
| Implications for future research | – Campaigns targeting Asian Americans should be "aware of the importance of alternative sources in reaching the population as well as the limited quality of such sources. The dissemination of accurate cancer screening and prevention information, customized to the population's needs through their preferred information sources should be prioritized, as well as offering more opportunities to increase health/cancer literacy skills."<br>– "Additionally, highlighting unique cancer risks to Asian American families and evidence-based messages focus on benefits of cancer screening may be more effective". |
| Other notes | N/A |
| Other | |
| Quality appraisal (JBI checklist) | ☒Include<br>☐Exclude |

| | |
|---|---|
| Review details | |
| Study ID (First author, year) | Bongaerts (2020) |
| Title of the review | Determinants of (non-)attendance at the Dutch cancer screening programmes: A systematic review |
| Review objectives/focus of the review | A systematic overview of the current known determinants of (non-)attendance at the Dutch cancer screening programmes. |
| Outcomes assessed | ☒Barriers *(described initially as 'determinants')*<br>☐Facilitators |
| Type of review | A review that seeks to include:<br>☒Qualitative studies<br>☒Quantitative studies<br>☐Mixed methods studies<br>☐Not specified |
| Systematic review | Do the authors state this is a systematic review, systematic scoping review, or other form of systematic evaluation?<br>☒Yes<br>☐No |
| Screening programmes targeted by the review | ☒Bowel<br>☒Breast<br>☒Cervical |
| Focus on specific method of screening? | N/A |
| Details of included studies | |
| Population(s) of the included reviews | Included characteristics:<br>☐Age:<br>☐Age within screening for NHS Scotland (50-70)?:<br>☒Gender and/or sex (described by the authors): Males and female (also described as women)<br>☒Ethnicity: Variable by study when reported; almost all studies included multiple ethnicities when noted by the authors<br>☒Other: Four studies were noted as including socioeconomic status details which varied from information on marital status, the |

| | |
|---|---|
| | role of immigration, and geographic residence within the Netherlands (including one study with mention of rurality). |
| Number of studies included | 19 |
| Total number of participants across all studies | 5,633,447 |
| Type of studies included | ☒Qualitative studies - 6<br>☒Quantitative studies - 13<br>☐Mixed methods studies<br>☐Not specified |
| Geographic scope (high-income countries only) | NLD (incl some rural) |
| Search strategy and methods | |
| Sources | Databases Academic Search Premier, Cochrane Library, Embase, EMCare, PubMed, PsycINFO, Web of Science. |
| Search restrictions (language, years, region etc) | Articles published before February 2018. |
| Search dates | Not reported. |
| Other criteria | A grey literature search as also conducted. See PROSPERO: CRD42018089444 for further details. |
| Instrument/tool used for quality appraisal of studies | ☒Yes: Crowe Critical Appraisal Tool or the Consolidated criteria for reporting qualitative research developed by the Dutch Cochrane Centre<br>☐Not specified |
| Characteristics of included studies table? | ☒Yes: See Supplementary Table 1 (available for download as a .pdf)<br>☐No |
| Method of analysis/synthesis of results | ☐Narrative synthesis<br>☐Thematic analysis<br>☒Other qualitative analysis: I-Change model used to categorize the identified determinants of cancer screening attendance.<br>☐Meta-analysis<br>☐Other quantitative analysis |
| Presentation of results supported by | ☐Tabulation<br>☒Framework/model: I-Change model<br>☐Forest plot<br>☐Other: |
| Key findings | |
| Key barriers and/or facilitators identified | A lack of tailored strategies was the only information factor noted across all three screening programs (BR/CE/BO)<br><br>Other information factors varying across programs included: non-GP practice-based invitation (CE); misconceptions, lack of knowledge (e.g., screening harm, CE/BO); low priority (CE/BO); perceived lesser risk of cancer (CE/BO); no future testing needed, less moral obligation (CE/BO); negative social influence/negative role models (CE); low self-efficacy (BR); forgot to make an appointment (CE); language barrier/low health literacy (BO)<br><br>Related to testing – test: insecure, anxious (CE/BO); outcome of the test: insecure, anxious (CE); inconvenience: feelings of shame |

| | |
|---|---|
| | (CE/BO); time related: forgot, too busy (CE/BO); other illnesses (BR) and financial barriers (BR)<br><br>See: Results, Table 3 |
| Limitations of the review | Not reported outside of acknowledgement that the review focuses specifically on the Netherlands. |
| Implications for future research | – "…a more prominent GP role in informing and activating people to participate in CSPs could be further explored."<br>– Future studies are needed that address socio-economic and ethnic health differences, given the system of Dutch healthcare and insurance. |
| Other notes | Only study incorporating multiple screening programmes to provide a breakdown of barriers specific to each. |
| Other | |
| Quality appraisal (JBI checklist) | ☒Include<br>☐Exclude |
