# Supplementary material for: What factors influence the uptake of bowel, breast and cervical cancer screening? An overview of international research
Source: Eur J Public Health. 2024 May 3;34(4):818–25. doi: 10.1093/eurpub/ckae073 (PMC11293835; doi:10.1093/eurpub/ckae073)
Supplement: ckae073_Supplementary_Data [file ckae073_supplementary_data.zip › ckae073_Supplementary_Data/ejph-2023-09-om-0509-File004.pdf]

Supplementary Material 3 – JBI checklist

Review ID	Q1	Q2	Q3	Q4	Q5	Q6	Q7	Q8	Q9	Q10	Q11
Ait Ouakrim (2013)	Y	Y	Y	Y	Y	Y	Y	Y	N/A	Y	Y
Alam (2021)	Y	Y	Y	Y	Y	Y	Y	Y	Y	Y	Y
Andreeva (2013)	Y	Y	Y	Y	Y	Y	Y	Y	Y	Y	Y
Baird (2021)	Y	Y	Y	Y	Y	Y	Y	Y	N/A	Y	Y
Biddell (2020)	Y	Y	Y	Y	Y	Y	Y	Y	Y	Y	Y
Bongaerts (2020)	Y	Y	Y	Y	Y	Y	Y	Y	U	Y	Y
Bromley (2015)	Y	Y	Y	Y	Y	Y	Y	Y	U	Y	Y
Byrnes (2020)	Y	Y	Y	Y	Y	Y	Y	Y	N/A	Y	Y
Chan (2017)	Y	Y	Y	Y	Y	Y	Y	Y	Y	Y	Y
Chorley (2017)	Y	Y	Y	Y	Y	Y	Y	Y	N/A	Y	Y
Connolly (2020)	Y	Y	Y	Y	Y	Y	Y	U	U	Y	Y
Cudjoe (2021)	Y	Y	Y	Y	Y	Y	Y	Y	Y	Y	Y
D'Onise (2020)	Y	Y	Y	Y	Y	Y	Y	Y	Y	Y	Y
Decruz (2021)	Y	Y	Y	Y	Y	Y	Y	Y	N/A	Y	Y
Dressler (2021)	Y	Y	Y	Y	Y	Y	Y	Y	N	Y	Y
Ferdous (2018)	Y	Y	Y	Y	N	Y	Y	U	N	Y	Y
Hendry (2012)	Y	Y	Y	Y	Y	Y	Y	Y	N/A	Y	Y
Jerome-D'Emilia (2015)	Y	Y	Y	Y	U	U	U	Y	N	Y	Y
Jerome-D'Emilia (2019)	Y	Y	Y	Y	U	U	U	Y	U	Y	Y
Jillapalli (2022)	Y	Y	Y	Y	Y	U	U	Y	Y	Y	Y
Jun (2018)	Y	Y	Y	Y	U	U	Y	U	U	Y	Y
Kandasamy (2021)	Y	Y	U	Y	U	Y	Y	Y	N/A	Y	Y
Kerrison (2021)	Y	Y	Y	Y	Y	Y	Y	Y	N/A	Y	Y
Kim (2018)	Y	Y	Y	Y	Y	U	Y	Y	N	Y	Y
Majid (2019)	Y	Y	Y	Y	Y	Y	Y	Y	N/A	Y	Y
McLachlan (2012)	Y	Y	Y	U	Y	Y	Y	Y	U	Y	Y
Nagendiram (2020)	Y	Y	Y	Y	Y	Y	Y	Y	Y	Y	Y
Nothacker (2022)	Y	Y	Y	Y	Y	Y	Y	Y	Y	Y	Y
Oh (2017)	Y	Y	Y	Y	Y	U	U	Y	U	Y	Y
Pagliarin (2021)	Y	Y	Y	Y	Y	Y	Y	Y	Y	Y	Y
Pariser (2022)	Y	Y	Y	Y	N	Y	Y	Y	N	Y	Y
Puli (2023)	Y	Y	Y	Y	Y	Y	Y	Y	U	Y	Y
Rogers (2015)	Y	Y	Y	Y	Y	Y	Y	Y	U	Y	Y
Rogers (2017)	Y	Y	Y	Y	Y	Y	Y	Y	Y	Y	Y
Tan (2018)	Y	Y	Y	Y	Y	U	Y	Y	N/A	Y	Y
Travis (2020)	Y	Y	Y	Y	Y	Y	Y	U	N/A	Y	Y
Wang (2019)	Y	Y	Y	Y	U	Y	Y	U	N	Y	Y
Wearn (2022)	Y	Y	Y	Y	Y	Y	Y	Y	N/A	Y	Y
Wortley (2014)	Y	Y	Y	Y	Y	Y	Y	Y	Y	Y	Y

YES (Y); NO (N); UNCLEAR (U); NOT APPLICABLE (N/A)

Full checklist:
© Joanna Briggs Institute 2017 Critical Appraisal Checklist for Systematic Reviews and Research Syntheses (jbi.global)
