# Supplementary material for: What factors influence the uptake of bowel, breast and cervical cancer screening? An overview of international research
Source: Eur J Public Health. 2024 May 3;34(4):818–25. doi: 10.1093/eurpub/ckae073 (PMC11293835; doi:10.1093/eurpub/ckae073)
Supplement: ckae073_Supplementary_Data [file ckae073_supplementary_data.zip › ckae073_Supplementary_Data/ejph-2023-09-om-0509-File006.pdf]

Maximising the trusted role of healthcare providers

Prioritising patient-centred care and patient autonomy

Culturally tailored health promotion

Systematically reducing health inequalities through policy

Informed research design

| KEY RECOMMENDATIONS AND CONCLUSIONS | |
|---|---|
| Bowel | |
| 1. Ait Ouakrim (2013) | Influence of clinicians shown to be key in adherence to health recommendations such as screening; "…any public health initiative intending to improve the level of CRC screening participation among those at increased risk due to their family history of the disease will be more effective if clinicians play a key role"<br><br>There is potential to increase the uptake of screening procedures "drawn from increasing the awareness of family history of CRC, such as reminding people with CRC diagnoses that their relatives may benefit from screening." |
| 2. Bromley (2015) | Ensuring that "providers appropriately recommend CRC screening to African Americans, and evaluate whether patients fully comprehend and recall the recommendation once delivered"<br><br>Confusion among choice of screening options as presented by physician counsel; presenting limited choices requires further investigation<br><br>Culturally tailored interventions to address procedural resistance<br><br>Multi-modal interventions beyond clinic into the community |
| 3. Chin (2020) | "Consistent effort by relevant authorities is needed to encourage the population to embrace the colorectal cancer screening experience"; this includes supplementing the use of FIT with pictorial guides, video, and mail delivery of the kit and results<br><br>Further required are "strong public policies that promote and create awareness about the use of FIT" |
| 4. D'Onise (2020) | Need for culturally competent health service access, including Indigenous health service providers |

| | |
|---|---|
| | Intervention studies should be conducted in partnership with Indigenous communities to improve participation, “ideally featuring and delivered by Indigenous people”<br><br>Health providers require additional training “to improve their knowledge of CRC and CRC screening, cultural training and generally encourage preventative healthcare”<br><br>Future studies may benefit from mixed methods; low-cost interventions (community lab drop-off of FOBT kits, telephone/text interventions) could be further considered<br><br>“Future studies would benefit from a mixed methods approach, including both high quality intervention studies alongside qualitative studies to explore barriers and enablers to screening participation.” |
| 5. Decruz (2021) | “An overall need is identified for a holistic approach to improve colonoscopy adherence rates”; this includes patient comfort, the use of clear instructional aids including graphics, the importance of patient rapport, and individualized options for procedural steps and use of sedation |
| 6. Dressler (2021) | “Involvement of general practitioners, implementation of media campaigns and the creation of a logistical support unit can result in higher participation rates” |
| 7. Kerrison (2021) | Further research is needed on the barriers and facilitators specific to surveillance and follow-up colonoscopy<br><br>A larger global focus is needed, as the majority of studies focused on the United States where screening programme delivery may differ from countries with socialized medicine<br><br>Interventions should focus on targets amenable to change – “e.g., ‘lack of understand that bowel cancer can be an asymptomatic disease” |
| 8. Kim (2018) | A “one-size-fits-all” approach will not be effective and tailored approaches that address cultural, psychological, healthcare-related barriers, and cues to action are needed for differing Asian American ethnic subgroups<br><br>Further research is needed “on the influence of ethnic culture’s behavioral norm”; this may lead to better understanding as to the role of family and friends in promoting screening programmes |
| 9. McLachlan (2012) | “…increased emphasis needs to be placed on efforts to improve the bowel preparation process, enhancing comfort and modesty during the examination and identifying patients with significant anxiety beforehand”.<br><br>Physicians “should be aware of the impact of their endorsement and be encouraged to discuss colon cancer screening and convey its importance to patients”. |

| | |
|---|---|
| 10. Puli (2023) | "When designing interventions to increase screening uptake among immigrants, gaps in physician and screening education, access to care, and trust need to be addressed through culturally sensitive supports."<br><br>"These interventions should be tailored to the specific immigrant group, since a one-size-fits approach fails to consider the heterogeneity within this population." |
| 11. Rogers (2015) | Focus on the role of communication (between health promoters and lay public, providers and patients, scientists and practitioners); "Specifically supported in our findings is the suggestion medical providers capitalize on their influence and join policy makers in efforts to eliminate CRCS disparities among AA men" |
| 12. Rogers (2017) | "It is important to acknowledge the need for both increased rigor and diversity in designing future research studies that will provide the highest quality of data to support practitioners, patients, policymakers, and scientific stakeholders."<br><br>"…future studies might consider a mixed-methods research design to capture the depth and breadth of health care experiences among diverse subsets of African American men, particularly those with fewer socioeconomic resources, to more comprehensively understand the intersection of race and CRC screening access and completion."<br><br>More research is needed in the areas of masculinity and sexuality, to further dispel homophobia associated with CRC screening |
| 13. Tan (2018) | Findings from the review should be further validated outside of the context of Europe and the United States<br><br>"One of our recommendations is to explore the possibility of actively engaging patients to be advocates for CRC screening amongst their family members"<br><br>"The various healthcare providers must also play an active role in identifying opportunities to relate the important message of CRC screening to the patients or directly to FDRs." |
| 14. Travis (2020) | Gap between intention and uptake should be further addressed in future qualitative research<br><br>Improve and enhance comfort and modesty to address 'procedural anxieties' (screening modifications)<br><br>Further investigation and trials needed on the 'nudge technique' to explore the 'decoy effect' in gender preferences for practitioner<br><br>Further appraisal needed of current UK interventions (avoidance due to low health literacy, 'think-aloud' studies may offer more immediate understanding of low uptake groups, community-based participatory research may be relevant to certain |

| | |
|---|---|
| | ethnic groups); "Appraisal of existing UK-wide NHS interventions to increase [flexible sigmoidoscopy screening] uptake, which are largely paper based, require further validation regarding their effectiveness on low uptake groups." |
| 15. Wang (2019) | Additional research needed on rural-urban differences in CRC screening barriers<br><br>Further studies should aim to distinguish barriers specific to FOBT as compared to endoscopies<br><br>"…findings suggest strategies providing public health education, reducing costs, and increasing access to specialists may be most effective in rural areas to promote CRC screening. An evaluation of these strategies and their impact on CRC screening can help inform policy interventions in rural areas". |
| 16. Wortley (2014) | Choice of screening tests does not always improve participation; using value-clarification tools (such as DCEs) can help "participants elucidate the characteristics of screening tests that are most important to them, thus aiding in the development of tailored communication strategies to assist in informed decision making regarding CRC screening options."<br><br>As a "significant proportion preferred no screening to the currently available screening tests for CRC" preference studies will continue to be relevant in shaping new screening programs and assisting with informed consent |
| Breast | |
| 1. Andreeva (2013) | "…well-targeted and culturally tailored efforts to increase screening mammography among recent EE immigrants are needed."<br><br>Critical to "expand the cancer prevention research and intervention agendas and to document the prevalence and determinants of BC-related health behaviors in that [Eastern European immigrant] vulnerable population, which could lead to increased efficacy of future initiatives."<br><br>Patients can become involved in their own healthcare and modify behaviours through the use of technology, including Internet sites and mobile applications |
| 2. Baird (2021) | The influence of barriers/facilitators for screening programme uptake may vary within different ethnic communities<br><br>"The patients' decision to attend screening is directly influenced by their encounters with healthcare staff. There is, therefore, a real need to better educate healthcare staff on the public health implications following their patient interaction." |
| 3. Jerome-D'Emilia (2015) | A physician's recommendation (cue to action) "has been found to be the strongest predictor of mammogram use across all ages and populations of women." |

| | |
|---|---|
| | "In order to encourage and support women in their health-seeking efforts, nurses must be cognizant of the factors that enable women to seek preventive care"; this includes education, poor breast cancer knowledge, insurance coverage and costs, and lack of a physician's recommendation |
| 4. Jerome-D'Emilia (2019) | "The qualitative studies suggest that women may be more responsive to locally supportive, targeted, and culturally appropriate interventions that respect traditionality, yet encourage trust in the medical system."<br><br>Specific to the United States, working with "tribes in the development of interventions framed by community-based participatory research are needed to tackle the disparities in the AI/AN community." |
| 5. Oh (2017) | "Additional research on effective interventions for improving screening among KAs is needed, but a trusted clinician will certainly have a positive impact on encouraging patients to seek screening."<br><br>"Other tools for increasing breast cancer screening among KA women may include culturally-tailored cancer education materials accessible to adults with limited English literacy that provide information about breast cancer and screening guidelines, the importance of regular screening in the absence of symptoms, and the procedures for scheduling and receiving breast screening. Social networks such as churches, cultural organizations, and KA doctors' offices can provide assistance with patient navigation, including supporting transportation and translation services." |
| 6. Pagliarin (2021) | "Due to the key role played by the staff's communication skills, future efforts to improve screening satisfaction should include the improvement of staff´s communication skills training."<br>Minimizing the wait time for screening results is optimal to alleviate potential concerns from participants with clear communication of expected timeframes<br><br>Screening facilities should prioritize privacy and create a welcoming environment within their physical space<br><br>"Implementing CBE as a regular screening method, in addition to a mammography might be helpful to improve the satisfaction and perceived quality of screening." |
| Cervical | |
| 1. Alam (2021) | "…importance should be given to assess the comprehensive understanding that immigrant women have about cervical cancer and its screening test, and develop interventions to improve it. One of the possible ways could be development of informative sources in the native language but steps need to ensure their availability and accessibility to the migrant women." |

| | |
|---|---|
| | Interventions to raise awareness among healthcare providers of a lack of cervical cancer screening among culturally and linguistically diverse communities is needed; “evidence suggests that targeted interventions for GPs involving elements of education and behavioral change can be helpful.”<br><br>The review found preference towards same-gendered healthcare providers and acknowledged that further steps should be taken to provide more access to female GPs for immigrant women, particularly those who have not yet acclimated to the Australian healthcare system (recently arrived immigrants, those living in Australian for a shorter period of time) |
| 2. Biddell (2020) | “Findings suggest that there is significant heterogeneity of patient preferences across populations and individuals, pointing to the importance of assessing preferences among individuals designed to benefit from a given intervention.”<br><br>Synthesis of included studies found a preference for HPV self-tests over Pap tests and preference for providers who “reflect patient gender, language, and life experience. These preferences must be recognized and leveraged by relevant stakeholders in the development of programs and policies to increase cervical cancer screening uptake among individuals most at risk.”<br><br>“Failing to account for the specific preferences of medically underserved individuals will allow the disparities in cervical cancer incidence and mortality to continue widening.” |
| 3. Chan (2017) | Multilevel models are needed to better understand screening behaviour including interpersonal, organizational, community, and the role of government and policy<br><br>“Government and policy makers might revise their strategies to promote screening uptake in other ways, rather than simply supporting testing costs and insurance coverage. Interventions directed at these levels may help to improve the screening uptake by increasing the facilities available and accessible, supporting childcare, having female physicians speaking the minority’s language, and overcoming culture-related influences that deter screening.”<br><br>“Policy makers or service providers may also consider their users’ concerns, such as the issues of trust and the confidentiality of personal information that US black women emphasize. Service providers should state clearly the strategies they use to maintain the confidentiality of personal information and implement the strategies as needed to help in building a trusting and positive relationship between the healthcare providers and black women.<br><br>“It may also be important to revise the public health education currently directed at black people”; this includes raising awareness of risk factors for cervical cancer as a means to change screening behaviour |

| | |
|---|---|
| 4. Chorley (2017) | “The variation in women's understanding and perceptions of cervical screening suggests that interventions tailored to decisional stage may be of value in increasing engagement with the invitation and uptake of screening in those who wish to take part.”<br><br>“There is also a need for further research with women who have never attended screening, especially those who remain unaware or unengaged, as their perspectives are lacking in the existing literature.” |
| 5. Christy (2021) | Experiences of Black women beyond the United States should be further explored as the results from the review are not wholly generalisable<br><br>“Being attuned to the historical and contemporary realities of sexual, physical, and psychological violence experienced by Black women (particularly how different elements of the screening process may trigger responses to earlier experiences for survivors of sexual trauma) can help clinicians to provide care that empowers and centers the preferences of the survivor. Such actions can create a clinical environment that feels compassionate and safe for Black women who are survivors of trauma. This may encourage more Black women to get screened earlier, effectively improving patient outcomes.”<br><br>Lessening the burden placed on Black women in obtaining screening such as providing bus tickets, on-site childcare, and pre-scheduling appointments<br><br>Clinicians must be aware of histories of clinical racism; “Elements of trauma-informed care, such as collaboration and empowerment, can not only help foster trust, but also encourage women to continue putting their health first.” |
| 6. Connolly (2020) | “Research examining the attitudes to cervical cancer screening among UK-based gender minorities AFAB is urgently needed, with a focus on provider and location preferences, and the acceptability of centralised National Health Service (NHS) databases that may be used to generate automatic invitations for screening.”<br><br>There is also an “urgent need for basic education surrounding the healthcare needs of gender minority patients AFAB, so that clinicians are responsive to each individual's needs and skilled in a range of approaches to cervical cancer screening.”<br>Policy documents are still lacking in the UK that further support gender minorities<br><br>Clinicians are encouraged to present a variety of techniques for screening to maximise patient autonomy |
| 7. Cudjoe (2021) | “Our study findings indicate the need for theory-guided, methodologically rigorous studies that use psychometrically tested instruments, utilize various forms of recruitment strategies (i.e. engage ethnic churches and organizations), and include larger samples of diverse groups of African immigrants (i.e. different African nationalities) to address the Pap testing behaviors and cancer health needs of AI women”. |

| | |
|---|---|
| | "..further research is also needed to help clinicians and researchers gain an in-depth understanding of why male healthcare providers may be barriers to Pap testing behaviors among AI women in developed countries." |
| 8. Ferdous (2018) | "…cancer screening-related information needs to be distributed and communicated in a culturally sensitive and linguistically appropriate manner. A proper explanation of the test procedure by physicians can help patients feel more comfortable and can alleviate their fears".<br>"Providing access to female healthcare providers for all immigrant women is challenging, but establishing culturally sensitive screening programs that provide timely access, particularly in immigrant-dense areas, can offer an effective solution."<br><br>There is a need to ensure physician knowledge of guidelines and recommendation for screening is up-to-date with adequate incentives for screening to be completed; "The presence of an effective reminder system for the primary care team and for patients can also be helpful"<br><br>Pathways to familiarize new immigrants with the healthcare system should be further investigated to ensure a preventative health focus<br><br>Future studies should focus on culture and social barriers for defined ethnic groups (no 'one-size-fits-all' approach) |
| 9. Hendry (2012) | Further development of educational materials, particularly around terminology, to inform consent is needed; "Identification of the salient points allowing informed choice to uptake from this complex information remains a challenge to researchers…explanations about causation, risk of cervical abnormality or cancer, persistence/clearance of monogenic HPV and difference from genital wart viruses are needed" |
| 10. Jillapalli (2022) | The studies included for review found that future research should "focus on cultural beliefs that influence cervical cancer screening behaviour. [This] will better inform the development of culturally sensitive educational interventions for both the AI population and for health care providers that provide care to minority populations." |
| 11. Kandasamy (2021) | Emphasis on culturally competent healthcare and trauma-informed care approaches; training approach should be prioritised that encourages "cultural competency training through teachings of colonial legacies, issues related to the intersectionality of race, gender, and social class, and Indigenous understandings of health and wellbeing."<br><br>"Practice changes that increase the building of trust through culturally competent healthcare, engaging in Indigenous-led research, and prioritising Indigenous perspectives in the implementation of policies, can collaboratively improve barriers and reinstate the facilitators of CCS participation, cervical cancer treatment, and survival." |

| | |
|---|---|
| 12. Majid (2019) | Concerns surrounding cervical screening uptake "may be managed with increased access and availability to alternative [healthcare providers] who are perceived by the woman as having the capacity to relate to her situation and appreciate the various factors that complicate CCS participation (gender, race, class, language)" |
| 13. Nagendiram (2020) | "General practitioners should offer opportunistic patient education and screening for women eligible to screening and facilitate conversations with under-screened women to overcome the barriers that prevent their participation[…]clinicians should also be offered education regarding screening guidelines in specific subgroups [including WSW and BMT survivors]."<br><br>Revisions to policy [in the Australian context] should also include further research and strategy development for WSW, older women, and migrant groups in addition to Aboriginal and Torres Strait Island women, immune-deficient women and women who have experienced sexual abuse |
| 14. Nothacker (2022) | Educational strategies should be targeted towards both men and women when considering STDs including key messaging around HPV infection; "This strategy should further explain changes in the screening procedure, including longer screening intervals and delayed age at first screening, and their consequences for the detection of cervical cancer."<br><br>Education strategies should further explore how to communicate the meaning of test results; "information about the meaning of a positive HPV test and the prevalence of HPV among the population should also be included in education strategies." |
| 15. Wearn (2022) | Review findings suggest that further action is needed to address structural barriers for underserved populations and the "role of policy makers and healthcare providers in ensuring underserved women feel safe, supported and able to participate in cervical screening services".<br><br>There is also a need to prioritise culturally sensitive communication strategies and tools, particularly in the UK context given evidence within the review that "UK health services often do not meet the needs of culturally diverse groups."<br><br>Future qualitative research should focus on those living in areas of high deprivation as "…developing this body of evidence would allow for further exploration of observed uptake inequalities and encourage identification of suitable targets for intervention."<br><br>Screening participation is reliant on a number of differing factors; further research is needed on the importance of social determinant on health behaviours as related to cervical screening participation |
| Breast and cervical (combined synthesis of findings presented by the author) | |
| 1. Byrnes (2020) | Highlights a need for a multidisciplinary approach to encouraging screening for WwLD and "should involve WwLD, family carers, and paid care workers including screening staff and GPs to ensure all [are] educated on cancer screening for WwLD". |

| | |
|---|---|
| | "…the findings from the review suggest that the health care of WwLD needs to be proactive and person-centred throughout the cancer screening pathway. This can include modifications to the invitations process, to not rely on literacy alone and utilise various communication aids including Makaton, or through additional resources such as visual recordings."<br><br>"Future research should concentrate on involving WwLD, family carers, and paid care workers to account for all three groups' attitudes and opinions together to obtain an in-depth understanding via qualitative methods." |
| 2. Pariser (2022) | "Pre-intervention work should focus on resources the community already has in place, as well as additional barriers that may be very specific to that community".<br>Barriers should be made further specific to unique communities; facilitators were not addressed in this review and may add further significant to reduce barriers and amplify care.<br><br>"The results of this review might also contribute to the development of advocacy tools to aid in the development of healthcare and migrant policy, as well as inform the declaration of government fiscal resources, be it local, state, or federal." |
| Bowel, breast and cervical (combined synthesis of findings presented by the author) | |
| 1. Bongaerts (2020) | "…a more prominent GP role in informing and activating people to participate in CSPs could be further explored."<br><br>Future studies are needed that address socio-economic and ethnic health differences, given the system of Dutch healthcare and insurance. |
| 2. Jun (2018) | Campaigns targeting Asian Americans should be "aware of the importance of alternative sources in reaching the population as well as the limited quality of such sources. The dissemination of accurate cancer screening and prevention information, customized to the population's needs through their preferred information sources should be prioritized, as well as offering more opportunities to increase health/cancer literacy skills."<br><br>"Additionally, highlighting unique cancer risks to Asian American families and evidence-based messages focus on benefits of cancer screening may be more effective". |
