# Supplementary material for: What factors influence the uptake of bowel, breast and cervical cancer screening? An overview of international research
Source: Eur J Public Health. 2024 May 3;34(4):818–25. doi: 10.1093/eurpub/ckae073 (PMC11293835; doi:10.1093/eurpub/ckae073)
Supplement: ckae073_Supplementary_Data [file ckae073_supplementary_data.zip › ckae073_Supplementary_Data/ejph-2023-09-om-0509-File007.pdf]

Category	Barrier	Key instances			Total
		BO	BR	CE	
1. Attitudes	Prevention activities not considered a priority	6	N/A	3	9
	Low perception that cancer is a risk because of a lack of symptoms	7	2	7	16
2. Communication	Healthcare provider does not discuss the use of all available testing methods	1	N/A	N/A	1
	Poor interpersonal skills of healthcare providers	N/A	2	1	3
	Lack of tailored information strategies surrounding best screening practices	3	1	4	8
	Information is provided in only one or very few languages	1	2	7	10
	Invitations for screening are not received from the healthcare system	1	N/A	1	2
3. Competence	Healthcare providers lack knowledge of the appropriate guidelines for screening	1	N/A	N/A	1
	Healthcare providers lack appropriate counselling skills	1	N/A	N/A	1
4. Discrimination	Experiences of discrimination in the healthcare setting, including racism	1	N/A	4	5
5. Fear	Fear of the unknown including a cancer diagnosis and/or finding something wrong	8	3	12	23
6. Knowledge	Inadequate knowledge about cancer	5	2	7	14
	Inadequate knowledge about cancer screening	9	2	7	18
	Low health literacy levels	3	3	3	9
	Low perceived self-efficacy, or a lack of confidence in the ability to understand and obtain cancer screening	1	N/A	N/A	1
	Relevance of screening as related to current state of health, life stage, and/or family history	1	N/A	1	2
7. Lived experience	Anxiety	N/A	N/A	1	1
	Competing medical issues	2	1	N/A	3

	Competing personal issues	2	N/A	N/A	2
	Embarrassment	2	N/A	1	3
	History of trauma	N/A	N/A	1	1
	No history of having a gynaecological exam	N/A	1	1	2
	Preference for traditional medicine over Western medicine	1	1	N/A	2
	Wider social setting including a lack of perceived privacy within a local community	1	N/A	1	2
8. Personal beliefs	Fatalism	6	3	4	13
	Religion	1	N/A	5	6
9. Procedure	Potential for procedural complications	2	N/A	1	3
	Preference for no screening versus currently available screening options	1	N/A	N/A	1
	Self-testing concerns including completing the test incorrectly and the validity of self-testing results	2	N/A	1	3
9a. Procedure – physical discomfort	Discomfort during the procedure, known from either past experiences or general perceptions of the procedure	5	1	3	9
	Test preparation difficulties, including unwelcome and inconvenient side effects from required medications	3	N/A	N/A	3
9b. Procedure – psychological discomfort	Psychological discomfort during the test itself including feelings such as embarrassment, vulnerability, shame, and/or anxiety	8	3	5	16
	Other perceived emotional or psychological barriers to undertaking the screening procedure (not specified)	1	N/A	2	3
10. Resources	Difficulties navigating the healthcare system including unclear messaging on where to go for screening	N/A	1	N/A	1
	Distance to testing facilities including concerns about transportation	1	4	6	11
	Inadequate access to a regular source of care, including a primary care provider	2	N/A	2	4
	Scheduling issues including forgetting to book/attend appointments, unsuitable appointment times, and time constraints such as work commitment or childcare concerns	4	5	9	18

	Shortage of healthcare providers	1	N/A	N/A	1
11. Social acceptance	Cultural related factors to the social acceptance of screening	4	3	5	12
	Lack of support provided by family members and/or social networks	4	1	4	9
	Social stigma	N/A	1	6	7
12. Trust	Distrust in the individual healthcare provider performing the test including gender and ethnic preferences not being met	1	1	8	10
	Lack of a recommendation from a healthcare provider to attend screening	3	1	N/A	4
	Lack of culturally competent healthcare	N/A	1	N/A	1
	Overall dissatisfaction with the quality of care and health services	3	N/A	5	8

Table 1: Barriers to the uptake of cancer screening services

Gradient colour key

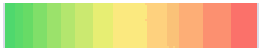

Least → Most observed

*Barriers that were frequently mentioned are coloured red, while those rarely mentioned are coloured green. Barriers that were not mentioned at all are coloured grey. A red square is therefore a hot spot for action.

**BO – Bowel; BR – Breast; CE - Cervical

Category	Facilitator	Key instances			Total
		BO	BR	CE	
1. Attitudes	Prevention activities considered a priority, including a pro-active desire to manage health concerns	1	N/A	N/A	1
2. Communication	Strong interpersonal skills of healthcare providers	1	1	3	5
	Tailored information strategies surrounding best screening practices	1	1	N/A	2
3. Competence	Provider is proficient in exam techniques	N/A	1	1	2
4. Knowledge	Functional health literacy	N/A	1	N/A	1
	Knowledge of cancer	1	2	N/A	3
	Knowledge of cancer screening	1	1	2	4
	Relevance of screening as related to current state of health, life stage, and/or family history	4	3	3	10
5. Lived Experience	History of screening, including positive screening experiences	2	N/A	N/A	2
	Knowing someone with cancer, including the strength of the relationship	1	N/A	N/A	1
6. Procedure	Ability to discuss results further with healthcare staff	1	N/A	N/A	1
	Accuracy or clinical effectiveness of the test	1	N/A	N/A	1
	No associated complications	1	N/A	N/A	1
	Option for alternative testing procedures, including self-testing	2	N/A	5	7
	Quick delivery of results	1	1	N/A	2
	Same-day screening	N/A	1	N/A	1
	Test is short in duration	1	N/A	N/A	1
	Use of pictorial communication in self-testing procedures	1	N/A	N/A	1

6a. Procedure – physical discomfort	No test preparation required	1	N/A	N/A	1
	Option for sedation	1	N/A	N/A	1
7. Resources	Access to a regular source of care, including a primary care provider	1	2	4	7
8. Social acceptance	Acculturation	1	4	5	10
	Acting as, or following, a role model	N/A	N/A	1	1
	Support provided by family members and/or social networks	6	3	4	13
9. Trust	Culturally competent healthcare	1	N/A	2	3
	Healthcare provider recommendation to attend screening	6	2	6	14
	Low perceived discrimination in the healthcare setting	1	N/A	N/A	1
	Overall satisfaction with the quality of care and health services	1	1	2	4
	Providers who reflect the lived experiences of patients including gender, ethnicity, and language preferences	N/A	N/A	5	5

Table 2: Facilitators to the uptake of cancer screening services

Gradient colour key

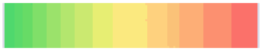

Least → Most observed

*Facilitators that were frequently mentioned are coloured red, while those rarely mentioned are coloured green. Facilitators that were not mentioned at all are coloured grey. A red square is therefore a hot spot for action.

**BO – Bowel; BR – Breast; CE - Cervical
