# Supplementary material for: What factors influence the uptake of bowel, breast and cervical cancer screening? An overview of international research
Source: Eur J Public Health. 2024 May 3;34(4):818–25. doi: 10.1093/eurpub/ckae073 (PMC11293835; doi:10.1093/eurpub/ckae073)
Supplement: ckae073_Supplementary_Data [file ckae073_supplementary_data.zip › ckae073_Supplementary_Data/ejph-2023-09-om-0509-File008.pdf]

Additional references

41. Jull J, Giles A, Graham ID. Community-based participatory research and integrated knowledge translation: advancing the co-creation of knowledge. Implementation Science. 2017;12(1):150.

42. Connolly D, Hughes X, Berner A. Barriers and facilitators to cervical cancer screening among transgender men and non-binary people with a cervix: A systematic narrative review. Prev Med. 2020;135:106071.

43. Ferdous M, Lee S, Goopy S, Yang H, Rumana N, Abedin T, et al. Barriers to cervical cancer screening faced by immigrant women in Canada: a systematic scoping review. BMC Womens Health. 2018;18(1):165.
